# Characterization of the influence of extraction factors on instant Pu-erh tea: Focusing on changes in sensory quality and aroma profile

**DOI:** 10.1016/j.fochx.2024.101925

**Published:** 2024-10-23

**Authors:** Guohe Chen, Yajie Xue, Guangmei Zhu, He Xie, Jing Zhang, Wanling Xiao, Chuyi He, Jianan Huang, Zhonghua Liu, Chao Wang

**Affiliations:** a Key Laboratory of Tea Science of Ministry of Education, Hunan Agricultural University, Changsha 410128, China; b National Research Center of Engineering and Technology for Utilization of Botanical Functional Ingredients, Hunan Agricultural University, Changsha 410128, China; c Co-Innovation Center of Education Ministry for Utilization of Botanical Functional Ingredients, Hunan Agricultural University, Changsha 410128, China; d Key Laboratory for Evaluation and Utilization of Gene Resources of Horticultural Crops, Hunan Agricultural University, Changsha 410128, China; e Ministry of Agriculture and Rural Affairs of China, Hunan Agricultural University, Changsha 410128, China; f Tea Cultivar Innovation Center, Yuelushan Laboratory, Changsha, Hunan 410128, PR China

**Keywords:** Instant Pu-erh tea, Key odor-active compounds, HS-SPME-GC × GC-TOF/MS, Relative odor activity value

## Abstract

The objective of this study is to explore the influence of extraction factors, including extraction temperatures, extraction time, and tea-water ratios, on the sensory quality and aroma characteristics of instant Pu-erh tea (IPET). Sensory evaluation, quantitative descriptive analysis (QDA) and HS-SPME-GC × GC-TOF/MS were utilized for analysis. The result showed that the optimal process condition of IPET was a tea-to-water ratio 1:8, an extraction temperature 75 °C, and an extraction time 60 min. A total of 235 volatile compounds were identified and 65 key odor-active compounds with ROAV>1 in all samples. Based VIP > 1, 19 key differential odor-active compounds were identified, including linalool oxide I, 1-dodecanol, linalool oxide II, etc. Further Pearson correlation analysis of key differential odor-active compounds and aroma characteristics showed that positive correlations between woody and ethyl nonanoate and 1-dodecanol, and between herbal and 1-methylnaphthalene. This research provides theoretical support for the production of high-quality IPET.

## Highlights

• Extraction affecting factors on the aroma quality of instant Pu-erh tea was explored.
• Quantitative descriptive and instrumental analysis were used to examine the factors.
• Nineteen key differential odor-active compounds were identified using ROAV and VIP.
• Correlation analysis was conducted on key odor-active compounds and aroma attributes.
• The optimal extraction conditions of instant Pu-erh tea were determined.

## 1 Introduction

Dark tea represents a distinct category of post-fermented tea in China. Varieties including Fu brick tea, Liupao tea, Pu-erh tea (PET), Qingzhuan tea, and Tibetan tea all fall under the category of dark tea (Chen et al., 2024). PET, a unique microbial post-fermented tea, which is produced in Yunnan Province. It is one of the exemplary representatives of dark tea, known for its “stale flavor” (Wang et al., 2022). It is crafted from sun-dried green tea of Yunnan large-leaf species. In accordance with different processing techniques, it can be subdivided into raw PET and ripened PET. Raw PET is produced by directly pressing and shaping the sun-dried green tea without undergoing post-fermentation and then drying. After a period of natural fermentation process, it can be transformed into aged PET. Ripened PET is produced by subjecting sun-dried green tea to a series of processes including wetting, pile fermentation, aging, and drying under artificially controlled humidity, temperature, and fermentation time (Wang, Li, Wu, et al., 2022). Since the 20th century, PET, with its unique flavor and quality as well as potential health benefits such as reducing blood fat and aiding in weight loss (Deng et al., 2023), exhibiting hypolipidaemic and antioxidatant effects (Ma et al., 2022), have garnered favor among consumers.

Instant Pu-erh tea (IPET) is made from ripened PET by the process of extraction, filtration, concentration, and drying (Wang et al., 2022). IPET exhibits advantages such as convenient brewing, portability, absence of pesticide residues, and retention of the flavor and health attributes of the original materials. As people's living standards continue to improve, traditional brewed tea is unable to meet the market demand. Consequently, an increasing quantity of PET is processed into instant tea products. Aroma and taste serve as significant indicators for assessing the quality of IPET. In recent years, a large number of studies have focused on the quality of IPET. During the preparation process of IPET, the loss rate of hetero‑oxygen compounds reaches as high as 96 %. Additionally, at each processing stage, both aroma components and taste components suffer losses to varying degrees (Liu et al., 2024). In the production of IPET through enzymatic hydrolysis, its content of tea soluble polysaccharides and free amino acids is notably higher than that obtained by water extraction and ethanol extraction methods. Moreover, it exhibits the highest product yield (Geng, Zhou, Guo, & Wang, 2013). The sensory quality of IPET obtained via electrostatic spray drying is similar to that achieved by freeze drying and is significantly superior to that of IPET produced by vacuum drying and conventional spray drying. Considering productivity and sensory quality aspects, spray drying emerges as a potentially viable method for the production of IPET (Wang, Li, Zhang, et al., 2022). The volatile compounds and sensory characteristics of spray-dried IPET were evaluated by employing headspace solid-phase microextraction (HS-SPME) in conjunction with comprehensive two-dimensional gas chromatography-time-of-flight mass spectrometry (GC × GC-TOF/MS). A total of 208 and 204 volatile compounds were respectively identified in IPET and PET. Among them, 153 compounds exhibited significant differences (*p* ≤ 0.05). Partial least squares discriminant analysis (PLS-DA) was utilized to identify 158 compounds that demonstrated differences between PET and IPET (Du et al., 2019). Aroma backfilling is currently an effective method that can effectively improve the aroma characteristics of IPET. Wang, Li, Zhang, et al. (2022) recovered the aroma-active compounds in PET by salting-out redistillation combined with sensory analysis and improved the flavor quality of IPET based on the release behavior of aroma-active compounds. Under optimized conditions, 41 volatile compounds were recovered in the first distillation, with a total recovery rate of 83.94 %. Forty-one odor-active compounds were recovered through salt-induced redistillation, and the total recovery rate reached 72.29 %, which was significantly better than that of the membrane method (33.46 %). This developed method can provide a more effective method for improving the flavor quality of IPET. Obviously, continuous improvement of processes and equipment is an effective approach to enhance the quality of IPET.

Extraction is a process whereby, in accordance with the principle that the active ingredients or functional components within a material are similar in nature and soluble in the extraction medium, solvents (including water) are utilized to extract active ingredients or functional components of diverse solubilities and polarities from the raw materials into the solvent, thereby realizing the separation of the active ingredients or functional components from the material substrate. The main factors that affect the extraction effect primarily include extraction temperature, extraction time, and tea-to-water ratio. In previous studies, it has been found that the yield of solids in the first extraction is four to five times that of the second extraction. The tea soup color, sensory quality, particularly in terms of solubility, of the product from the first extraction are markedly superior to those from the second extraction. Employing one-time extraction technology can increase production efficiency, lower production costs, and obtain high-quality instant products (Huang et al., 2003). Consequently, in this study, IPET is prepared by means of one-time extraction. During the processing of IPET, changes in these factors can give rise to the leaching of certain substances, thereby altering the original flavor and aroma substances of tea. This gives rise to issue such as “low aroma intensity, dark coloration, and inferior taste” in IPET. Preparing high-quality IPET has become a difficult problem that the industry needs to solve.

HS-SPME is widely used in the preparation of volatile and semi-volatile substances in tea due to its advantages such as short operation time, small sample size, and good repeatability. In terms of detection technology, with the rapid development of chromatographic technology, GC × GC-TOFMS technology is widely used in food aroma detection (Gogus, Ozel, Kocak, Hamilton, & Lewis, 2011; Kiefl, Pollner, & Schieberle, 2013; Weldegergis et al., 2011), pesticide residue detection (Jiang et al., 2011), and other fields with its characteristics of high sensitivity, high resolution, and large amount of information. At present, it has also spread to the tea field. For example, Zhang et al. (2013) applied GC × GC-TOF/MS technology to tea detection and conducted a comparative study on the volatile components in green tea, oolong tea, and black tea in combination with multivariate statistical analysis, successfully achieving a clear distinction among the three types of tea. Zhu et al. (2015) performed an analysis and comparison of Longjing tea by employing the combined technology of GC × GC-TOFMS and GC–MS. It was discovered that GC × GC-TOFMS could identify 522 odor-active compounds, which was five times the number identified by GC–MS, thereby demonstrating its formidable separation performance. Relative odor activity value (ROAV) is an important method for calculating the contribution degree of volatile compounds to the formation of tea aroma, which has been widely applied in tea research (Chen et al., 2024; Li et al., 2024).

At present, the influence of extraction factors on the sensory quality and flavor profile of IPET has been rarely studied. Therefore, this study aims to achieve the following objectives: (1) to explore the influence of extraction factors, including extraction temperatures, extraction time, and tea-water ratios, on the sensory quality and aroma characteristics of IPET; (2) to identified the key aroma compounds present in all IPET samples using HS-SPME/GC × GC-TOF/MS combined with ROAV; (3) to explore the key differential aroma compounds by chemometrics approaches.

## 2 Materials and methods

### 2.1 Materials and reagents

#### 2.1.1 Experimental materials

Eight PET samples (Y) for this experiment are selected from Yunnan Tasly Deepure Biological Tea Group Co., Ltd. Considering the actual production situation, a three-factor and three-level L₉(3^3^) orthogonal test (Table S1) is designed with different tea-to-water ratios, extraction temperatures, and extraction time as indicators. After being uniformly mixed, the tea samples are processed into samples through extraction and vacuum freeze drying. All the prepared samples are independently packaged in sealed bags and stored in a − 80 °C refrigerator for testing before the experiment.

#### 2.1.2 Instruments and reagents

Electronic balance (AE240), Mettler, Switzerland; Digital display magnetic heating stirrer, Beijing Kanglin Technology Co., Ltd.; *n*-alkanes (C7-C28, 99 %), Supelco, USA; Ethyl caprate (99.9 %), Sigma, USA. Digital display magnetic heating stirrer, Beijing Kanglin Technology Co., Ltd.; Solid-phase microextraction handle (SPME), Supelco, USA; Solid-phase microextraction fiber (50/30 μm Divinylbenzene/Carboxen/Polydimethylsiloxane, DVB/CAR/PDMS), Supelco, USA; GC × GC-TOFMS, Agilent, USA; CTC Analytics autosampler, Guangzhou ingenious Laboratory Technology Co., Ltd.; Vacuum freeze dryers (Alpha 1–4 LSCplus), Martin Christ Co., Ltd., Germany.

### 2.2 Experimental methods

#### 2.2.1 Sample preparation

300 g of PET mixed sample → sieving with 400-mesh non-woven fabric → one-time water extraction → freezing in a − 4 °C refrigerator for standby → vacuum freeze drying → IPET sample→ freezing in a − 80 °C refrigerator for standby.

#### 2.2.2 Sensory evaluation and quantitative descriptive analysis (QDA)

The sensory evaluation group is composed of 10 professional tea evaluators (6 women and 4 men), aged between 20 and 60 years old. They have a certain degree of acuity for aroma and taste and are proficient in sensory evaluation models. Referring to the evaluation methods of instant tea powder in previous study (Yang, Guo, Wang, Zhou, & Mo, 2016) and GB/T 31740.1-2015 (Tea products-Part 1: Solid instant tea), the prepared IPET sample are brewed with 0.5 g tea powder and 150 mL water. The main evaluation factors are the color, aroma, and taste of the tea soup. Among them, the color accounts for 30 %, the aroma accounts for 30 %, and the taste accounts for 40 %. A hundred-point system is used for scoring.

For QDA, according to the tea sensory evaluation method and evaluation terminology, and referring to the terminology of our previous research (Chen et al., 2023). The aroma intensity is scored on a 10-point scale, where “0” represents no aroma, “1” represents weak aroma, “5” represents moderate aroma, and “10” represents the highest aroma intensity. Finally, the average score of sensory evaluation is used as the intensity of each aroma characteristic in the sample.

#### 2.2.3 HS-SPME conditions

The extraction of volatile compounds of IPET samples were extracted via the HS-SPME method. The HS-SPME procedures were referred to a previous study (Wen et al., 2023). Accurately weighting of 0.2 g of IPET sample was done into a 20 mL headspace vial. And then, a magnetic rotor, 10 μL of ethyl decanoate (8.63 mg/L), and 5 mL of boiling water were sequentially added. Quickly tighten the cap and place it on a magnetic stirrer. After equilibration at 80 °C and 200 r·min^−1^ for 10 min, a 50/30 μm DVB/CAR/PDMS coating fiber (Sigma-Aldrich Trading Co., Ltd., Shanghai) was used to absorb volatile compounds for 30 min. Subsequently, the coating fiber was desorbed in the gas chromatograph inject port at 250 °C for 10 min. Before sampling, the coating fiber was heated and aged at 250 °C for 30 min under the fiber conditioning program, and then 2 blank shots were run to confirm the absence of impurity peaks. HS-SPME was completed using CTC automatic sampling device.

#### 2.2.4 GC × GC-TOF/MS conditions

GC × GC conditions: The volatile compounds adsorbed by the CAR/PDMS-coated fiber were separated using the 8890 GC × GC instrument (Agilent, California, USA). The one-dimensional (1D) and two-dimensional (2D) columns employed were an HP-5MS capillary column (30 m × 250 μm × 0.25 μm, Agilent Technologies) and a DB-17MS capillary column (2.89 m × 180 μm × 0.18 μm, Agilent Technologies), respectively (Wen et al., 2023). The temperature of the injection port was set at 250 °C, and the temperature of the transfer line was 280 °C. Helium (99.999 %) served as the carrier gas, with flow rates of 1 mL/min and 1.5 mL/min for the two columns, respectively. A solid-state modulator SSM1800 (J&X Technologies, Shanghai, China), containing HV series modulation columns (1.3 m × 0.25 mm; C5-C30; J&X Technologies), was placed between the 1D and 2D columns for concentrated aggregation and release, with a modulation period of 4.0 s. The heating program was as follows: initially held at 40 °C for 1 min, then ramped up to 180 °C at a rate of 4 °C/min, and finally ramped up to 250 °C at a rate of 20 °C/min and maintained for 1 min, with a total runtime of 40.5 min. The transfer line temperature was 280 °C, the injection port temperature was 250 °C, and the split ratio was 10:1 (Wen et al., 2023).

TOF/MS conditions: The 7250 TOF/MS (Agilent Scientific, CA, USA) was used to acquire volatile mass spectral information (Wen et al., 2023). In the acquisition process, full scan mode with a range of 45–500 amu, a frequency of 50 spectra/s, and a filament current of 5 μA was utilized. For the ionization process, an electron bombardment ion source (EI) was adopted. The ion source temperature was set at 200 °C, and the electron energy was 70 eV. Additionally, the mass spectrometer interface temperature was set at 280 °C, the quadrupole temperature at 150 °C, and the solvent delay at 3 min.

#### 2.2.5 Identification and quantification of volatile compounds

The GC × GC-QTOFMS data were analyzed using Canvas software (version 1.0.0.25117). N-alkanes (C7-C19) were utilized for calibrating and calculating the retention index (RI) during the qualitative analysis of volatile compounds. The mass spectrum information of each chromatographic peak was then compared to ‘mainlib’, ‘replib’, and ‘nist_ri’ in the NIST20 mass spectral library. And then peak tables of samples were compared using the statistical comparison function in Canvas software. Retentions of volatile compounds with positive match >700, reverse match >800, and RI deviation < 30 were identified. The relative concentration was calculated using the internal standard method and the formula is as follows:

$$C_{i}\left(\mathrm{\mu g}/\mathrm{kg}\right)=\frac{\mathrm{Ratio}\times 10\mathrm{ul}\times 8.63\mathrm{mg}/L}{M.}$$

In the formula, C_*i*_ is the relative concentration (μg/kg) of each odor compound; Ratio is the ratio of the peak area of the compound to the peak area of the internal standard; *M* is the mass of the tea sample, 0.2 g. Each sample is repeatedly detected three times.

#### 2.2.6 Calculation of ROAV

Based on the quantification of each volatile compound, search, consult and compare the minimum thresholds of compounds in currently reported literature. ROAV is equal to the ratio of the content of each compound to its minimum threshold of the reported in teas (Chen et al., 2024).

### 2.3 Statistical analysis

Use Excel 2021 software for volatile component data analysis. The test results are expressed as “mean ± standard deviation”. Use SIMCA-P for principal component analysis (PCA), hierarchical cluster analysis (HCA), and partial least squares discriminant analysis (PLS-DA). Use Origin 2021 software to draw percentage stacked charts and radar charts. Use TBtools software to draw heatmap cluster analysis. Use online tools (www.omicstudio.cn) to draw correlation network diagrams.

## 3 Results and discussion

### 3.1 Analysis of sensory quality of IPET with different extraction processes

In order to determine the optimal extraction process for IPET, on the basis of previous research and in combination with production practice, three factors including extraction temperature, extraction time, and tea-to-water ratio were selected in this study for L9 (3^3^) orthogonal experimental design to prepare IPETs with different extraction processes. The prepared IPETs were evaluated by sensory evaluation using a comprehensive evaluation method. The results showed that there were certain differences in aroma, soup color, and taste among IPETs prepared by different extraction processes (Table 1). Among the IPET samples, IPET-2 has a relatively high total score. In terms of aroma, it presents a pure aged fragrance. In terms of soup color, it is dark reddish-brown. In terms of taste, it is mellow, smooth, and has a sweet aftertaste. IPET-8 ranks second. As the extraction temperature increases, the soup color gradually deepens. At the same time, a high-fire flavor appears in the aroma, which is similar to previous studies (Liu et al., 2023).

**Table 1:** Sensory quality characteristics of IPET produced by different extraction processes.

Samples	Aroma(30 %)		Soup color(30 %)		Taste(40 %)		Total Score	Yield(%)
	Comment	Score	Comment	Score	Comment	Score		
Y	Strong stale aroma with sweet aroma and herbal aroma	91	Reddish-brown	90	Mellow with sweet aftertaste	90	90.3	–
IPET-1	Weak stale aroma	82	Brownish-red-brown	83	Relatively mellow with a slight astringency	85	83.5	12.06
IPET-2	Pure stale aroma	92	Dark reddish-brown	91	Mellow, smooth, and with sweet aftertaste	92	91.7	15.97
IPET-3	Stale aroma with high-fire odor and herbal aroma	88	Dark reddish-brown	91	Mellow with sweet aftertaste and a slight astringency	86	88.1	18.29
IPET-4	Stale aroma and sweet aroma	89	Brownish-red-brown	87	Relatively mellow	84	86.4	14.61
IPET-5	Stale aroma with a little sweet aroma	88	Dark reddish-brown	91	Mellow with sweet aftertaste	90	89.7	18.33
IPET-6	Stale aroma with high-fire odor	86	Dark reddish-brown	91	Mellow with sweet aftertaste	90	89.1	20.39
IPET-7	Stale aroma	85	Brownish-red-brown	87	Relatively mellow	84	85.2	8.71
IPET-8	Pure stale aroma	92	Dark reddish-brown	91	Mellow with sweet aftertaste	90	90.9	19.34
IPET-9	Slight stale aroma, high-fire odor, and smoky and burnt odor	82	Dark reddish-brown	91	Mellow with sweet aftertaste and a sour note	86	86.3	22.48

### 3.2 Orthogonal test analysis of IPET with different extraction processes

In order to determine the optimal extraction process for IPET, orthogonal test analysis was carried out using sensory evaluation indicators to optimize the processing technology of IPET. Based on production practice, this study selected three factors including tea-to-water ratio, extraction temperature, and extraction time for L9(3^3^) orthogonal test design to prepare IPETs respectively. The factors and levels of the orthogonal test are shown in Table S2.

In this experiment, range analysis was used to compare and analyze the magnitudes of K, k, and R values (range) of the extraction temperature, extraction time, and tea-to-water ratio. According to the magnitude of the range, the factors are ranked in order of importance. The larger the range, the greater the influence of the factor on the quality of IPET. As can be seen from Table S3, the order of influence of three factors on the quality of IPET are as follows: extraction temperature > extraction time > tea-to-water ratio. Based on the sensory evaluation score, the level corresponding to the maximum value among k1, k2, and k3 is selected as the optimal processing technology factor level. Through comprehensive analysis, the optimal process for IPET processing is A_2_B_2_C_2_, that is a tea-to-water of 1:8, an extraction temperature of 75 °C, and an extraction time of 60 min. The optimal process combination obtained by range analysis is similar to previous studies (Geng et al., 2013).

Furthermore, IPET-10 was prepared according to the optimal extraction conditions and sensory evaluation was conducted. The results showed the total score of IPET-10 is higher than that of other instant tea samples (Table S4). In terms of aroma, it has a pure aged fragrance with a sweet aroma, and the total score is 92.4. In terms of taste, it is mellow, smooth, sweet, and has a sweet aftertaste. Moreover, the yield of IPET is 18.84 %. This result was consistent with the large-scale production of IPET. Therefore, the optimal process conditions for different IPET samples are a tea-to-water ratio of 1:8, an extraction temperature of 75 °C, and an extraction time of 60 min. This result can be used as a reference for the extraction process of IPET.

### 3.3 Sensory quantitative descriptive analysis of IPET with different extraction processes

**Fig. 1:**
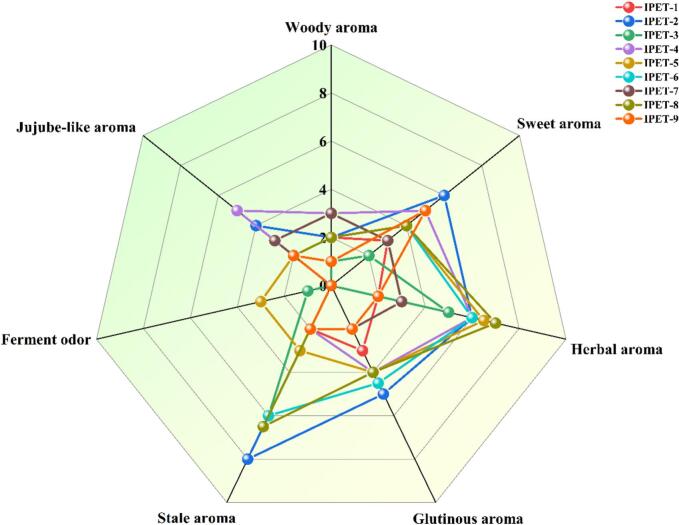
QDA of IPET with samples.

IPET retains the original flavor attributes of PET. Therefore, based on the establishment of the main sensory description words of PET (Chen et al., 2023), this study further uses QDA to score the aroma intensity of IPET with different extraction processes. As shown in Fig. 1, these sensory description words can better describe their sensory flavors. Judging from the shapes shown in the radar charts, the grid shapes surrounded by the aroma attribute intensity values of IPETs with different extraction processes are all different and have obvious characteristics. In terms of aroma, IPET-5 is relatively prominent in herbal aroma; IPET-2 is relatively prominent in sweet aroma and stale aroma; IPET-4 is relatively prominent in jujube-like aroma.

### 3.4 Volatile compounds analysis of IPET samples

IPET samples were detected by HS-SPME-GC × GC–MS and compared with the NIST atlas library and references (Table S5). Two hundred and thirty-five volatile compounds were detected, these compounds could be divided into aldehydes (30), alcohols (29), esters (19), ketones (47), acids (3), phenols (12), hetero‑oxygen compounds (18), pyrroles and their derivatives (20), hydrocarbons (51), and other compounds (6). Compared with the previously reported analysis of the aroma of IPET by HS-SPME combined with GC–MS, the application of HS-SPME combined with GC × GC-TOF/MS significantly increased the number of qualitatively identified compounds from 88 to 218, indicating that GC × GC-TOF/MS with higher sensitivity has certain advantages in the qualitative analysis of aroma compounds in IPET and can detect more compounds related to the formation of these aromas (Wang, Li, Zhang, et al., 2022).

**Fig. 2:**
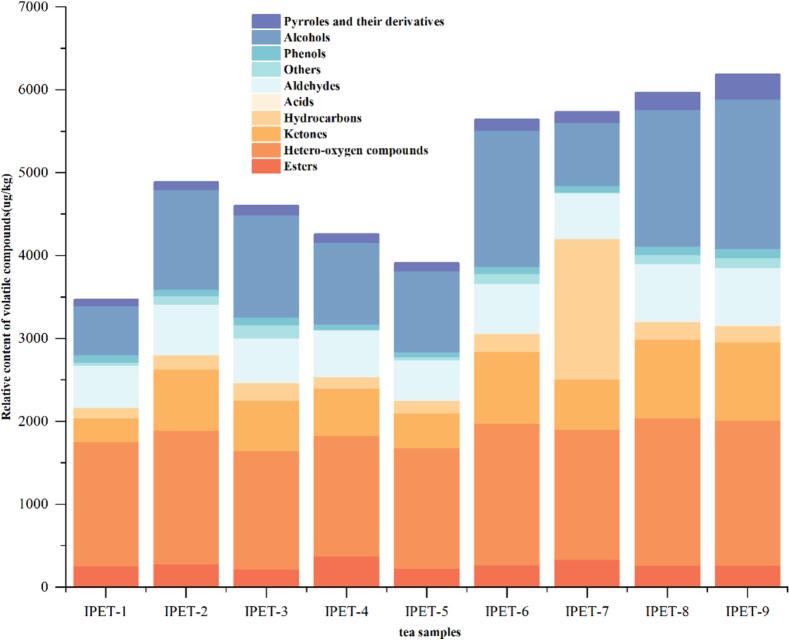
Analysis of volatile compound composition in IPET with different extraction processes.

**Fig. 3:**
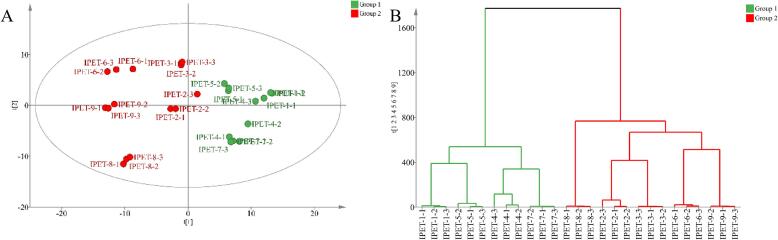
PCA (A) and HCA (B) of volatile compounds in instant IPET samples.

In order to study the differences in the content of volatile compounds in IPET with different extraction processes, a stacked column chart was drawn (Fig. 2). There are certain differences in the content and types of volatile compounds in nine IPETs with different extraction processes, which are closely related to the extraction parameters. Among them, IPET-9 (6188.91 μg/kg), IPET-8 (5971.67 μg/kg), IPET-7 (5733.33 μg/kg), IPET-6 (5644.12 μg/kg), and IPET-2 (4887.21 μg/kg) have relatively high contents compared to other tea powders. The relative contents of hetero‑oxygen compounds, alcohols, aldehydes, and ketones are >10 %. Among hetero‑oxygen compounds, 1,2,3-trimethoxybenzene, 1,2,4-trimethoxybenzene, and 1,2,3-trimethoxy-5-methylbenzene have relatively high contents. These compounds were also found in our previous studies (Wang, Li, Zhang, et al., 2022). Among alcohol compounds, linalool, linalool oxide I, linalool oxide II, and 1-dodecanol have relatively high contents; among aldehyde compounds, nonanal and safranal have relatively high relative contents; among ketone compounds, γ-ionone and (*E*,*E*)-3,5-octadien-2-one have relatively high relative contents. These compounds may be related to the formation of the aroma of IPET. PCA and HCA can better obtain the differences and similarities between the volatile components of IPET with different extraction processes and are an effective method for data dimension reduction and visualization (Chen et al., 2024). The results of PCA and HCA analysis show that (fitting parameters are R^2^X = 0.912, Q^2^ = 0.68) (Fig. 3 AB). There are obvious differences in the volatile compounds of nine IPETs with different extraction processes and can be divided into two groups. IPET-1, IPET-5, IPET-2, and IPET-7 are clustered into one group; IPET-8, IPET-2, IPET-3, IPET-6, and IPET-9 are clustered into one group. The differences in the types and contents of volatile components of IPET with different extraction processes are closely related to the extraction parameters.

### 3.5 ROAV analysis of IPET with different extraction processes

At present, more than ten thousand odor components have been found in the food field. However, only a small part plays a key decisive role in the formation of the overall aroma of food. This part of odor components is called “key odor-active compounds”. Flavor chemistry researchers believe that odor compounds with ROAV>1 are usually considered to contribute to the overall aroma of the analyzed sample, and odor compounds with ROAV>100 are considered to have outstanding contributions to the formation of the overall aroma of the analyzed sample (Chen et al., 2024). In order to further screen the odor compounds that play a major role in the overall aroma formation of IPET under different extraction conditions, the ROAV of each odor compound was calculated based on the quantification of odor compounds combined with the reported thresholds of odor compounds in aqueous solutions. However, at present, the thresholds of odor compounds in water vary. For example, when calculating the OAV of nonanal, Gong et al. (2017) and Yang et al. (2023) used 2530 μg/L and 0.0011 μg/L respectively, a difference of 2,300,000 times. Similarly, when calculating the OAV of linalool oxide I, Wang et al. (2022) and Zhu, Niu, and Xiao (2021) used 0.0038 μg/L and 100 μg/L respectively, a difference of 26,316 times. It can be seen that different choices of compounds thresholds will lead to different magnitudes of calculated ROAV and key odor-active compounds for tea aroma formation will be omitted. Therefore, in this study, the minimum thresholds of compounds reported so far in water are selected to select key odor-active compounds in IPET to the maximum extent. After querying the literature and comparing the magnitudes of thresholds, 124 volatile compounds with relatively small thresholds were found.

**Fig. 4:**
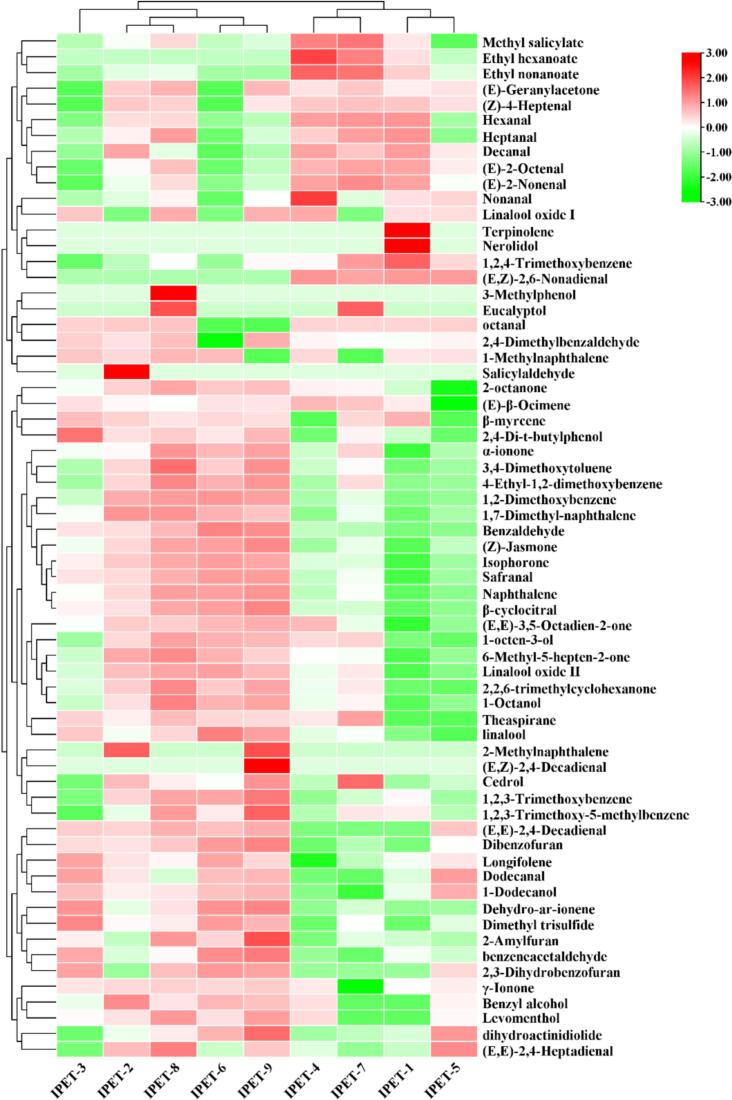
Heatmap cluster analysis of key aroma compounds in IPET with different extraction processes.

There are 65 key odor-active compounds with ROAV>1 in all IPET samples (Table S6). Among them, IPET-1, IPET-2, IPET-3, IPET-4, IPET-5, IPET-6, IPET-7, IPET-8, and IPET-9 have 48, 54, 52, 51, 50, 49, 49, 55, and 52 volatile compounds with ROAV>1 respectively. Among these, 35 compounds are present in all IPETs, 14 compounds with ROAV>100, including 1,2,3-trimethoxybenzene (623.91–745.86), 1,2,4-trimethoxybenzene (101.25–101.25), 6-methyl-5-hepten-2-one (104.12–479.39), (*E*,*E*)-3,5-octadien-2-one (182.47–847.93), (*Z*)-jasmone (229.994–1119.727), *α*-ionone (70,810.01–207,043.77), hexanal (3488.73–7943.95), heptanal (258.84–547.22), (*E*,*E*)-2,4-heptadienal (317.19–997.81), benzeneacetaldehyde (82,055.93–128,332.10), decanal (290.96–695.56), 1-octanol (116.74–499.90), linalool oxide II (312.27–2009.24), and linalool (367.14–1087.62). These compounds have outstanding contributions to the formation of the aroma of IPET. It is worth noting that among these compounds, (*Z*)-jasmone (OT = 0.007 μg/L), *α*-ionone (OT = 0.0004 μg/L), Hexanal (OT = 0.005 μg/L), and benzeneacetaldehyde (OT = 0.0003 μg/L) are found to have extremely low thresholds. These compounds have a higher degree of contribution to the formation of the aroma of IPET compared to others. In addition, heatmap cluster analysis was used to analyze the differences of these key aroma compounds (Fig. 4). Among them, IPET-2 and IPET-8 are clustered into one category. This is consistent with the sensory evaluation results and further verified the reliability of the experimental results. In IPET-1, the content of terpinolene and nerolidol is relatively high; in IPET-2, the content of salicylaldehyde is relatively high; in IPET-3, the contents of 2,4-di-t-butylphenol and dimethyl trisulfide are relatively high; in IPET-4, the contents of ethyl hexanoate, nonanal, and ethyl nonanoate are relatively high; in IPET-6, the content of linalool is relatively high; in IPET-7, the contents of cedrol and methyl salicylate are relatively high; in IPET-8, the contents of 3-methylphenol and eucalyptol are relatively high; in IPET-9, the contents of 2-methylnaphthalene and (*E*,*Z*)-2,4-decadienal are relatively high. It could be seen that there are significant differences in the contents of key aroma compounds in IPET with different extraction processes, which determines the difference in their aroma quality.

The origins of most volatile compounds could be tracked, which were mainly carotenoid and lipid derivatives, and glycoside-derived aromas (Ho, Zheng, & Li, 2015). Methoxybenzene compounds have outstanding contributions to the formation of the aged aroma of dark tea, which has been verified in previous studies (Lv et al., 2012). During the storage process of Pu-erh tea, methoxybenzene compounds usually have an aged flavor, and their content significantly increases in a hot and humid environment. For example, 1,2,3-trimethoxybenzene, 1,2,4-trimethoxybenzene, 4-ethyl-1,2-dimethoxybenzene, 1,2-dimethoxybenzene, 3,4-dimethoxytoluene, and 1,2,3-trimethoxy-5-methylbenzene. Their concentrations considerably increased over time in the wet-hot environment but not in the dry-cold environment. These compounds are identified as potent odorants with an OAV > 1 (Xu et al., 2021). These compounds were described as stale odor and had been discovered and identified to make great contributions to the formation of the dark tea aroma in previous studies (Liu et al., 2022; Ma et al., 2023; Pang et al., 2019; Zhang et al., 2019). Alcohols are important compounds known for their floral and woody fragrances. 1-Octen-3-ol with earthy, green, oily, vegetative-like, and fungal odors was identified as a key aroma compound in oolong tea (Guo, Schwab, Ho, Song, & Wan, 2022). Linalool and its oxides (I, II, and III) were found to contribute to these scents, partly resulting from the oxidation of linalool, a compound that increases during the pile-fermentation process in dark tea (Hu et al., 2021). Cedrol, provided woody odor, which was identified as one of the key aroma compounds in the formation of the unique flavor of PET, and the ‘fungal flower’ aroma of Fu brick tea processing in Hunan province was dominated with ‘floral’, ‘woody’, and ‘green’ attributes (Li et al., 2020; Lv et al., 2012). Aldehydes were thought to be mainly produced via lipid oxidation and decomposition (Barra et al., 2007). Benzaldehyde, described as almond-like odor, generally comes from the hydrolysis of aroma glycosides, which was detected in PET with a high odor intensity. Benzeneacetaldehyde was described as floral and honey odors and detected in IPET with a high FD factor, which had been considered as the production of the Maillard reaction (Ho et al., 2015; Xu et al., 2016; Zhang et al., 2019). The oxidation of linolenic acid form nonanal and decanal that decanal with herbal odor was considered to be the most important component due to its high odor intensity in PET (Lv et al., 2012). Nonanal was considered to be one of the common odor-active compounds in PET (Xu et al., 2016). Heptanal with pungent odor was considered to be key aroma compound in the condensed water of PET. Meanwhile, safranal can be used as the key characteristic volatiles to reflect the formation of aged fragrance of Qingzhuan tea (Zhang et al., 2021). (*E*,*E*)-2,4-Heptadienal with fatty and waxy odors was the basic aroma compound and made huge contributions to the formation of the overall aroma components of Liupao tea (Ma et al., 2023). (*E*,*Z*)-2,6-Nonadienal with cucumber odor and was considered to be extremely potent aroma compounds in PET with a threshold of 0.2 μg/L (Xu et al., 2016). Ketones play an essential role in the formation of dark tea due to its low odor threshold as well as almost each of them emits unique odors. *α*-Ionone provided woody odor underwent remarkable changes during pile fermentation and could be used as potential odor-active markers for ripened PET and raw PET discrimination (Pang et al., 2019), and it was considered as great contributors to QZT aroma in previous studies (Liu et al., 2022). 6-Methyl-5-hepten-2-one with citrus and strawberry odors was identified as key aroma compounds in Sichuan dark tea and Sichuan Fuzhuan brick tea (Nie et al., 2019). (*E*,*E*)-3,5-Octadien-2-one with fruity odor was identified in LPT, and is a potent VCs with relatively low odor thresholds (Ma et al., 2023; Wang, Li, Wu, et al., 2022). (*Z*)-Jasmone has been reported to impart a characteristic floral jasmine odor, and it is widely distributed in many green teas (Lee et al., 2013). Compared with methoxy-phenolic compounds, phenols were found very little in the study. Among them, 2,4-ditert-butylphenol has been detected in previous studies and is usually described as having a woody aroma attribute (Ma et al., 2022). Finally, esters, hydrocarbons, acids, pyrroles and their derivatives were detected in relatively small quantities in this study. Methyl salicylate with minty odor is very crucial to the flavor of tea which is derived from enzymatic hydrolysis (Li et al., 2020). The content of methyl salicylate in tea was also greatly enhanced by the treatment of microbial enzyme. 1-Methylnaphthalene with pungent odor was reported to contribute to the aroma of Liupao tea, which was usually used as a potential indicator to evaluate the degree of fermentation (Wu et al., 2016). Dihydroactinidiolide provided woody and floral odors had a low aroma intensity during the processing of Qingzhuan tea (Liu et al., 2022).

### 3.6 Analysis of key differential compounds in IPET with different extraction processes by PLS-DA and correlation analysis

**Fig. 5:**
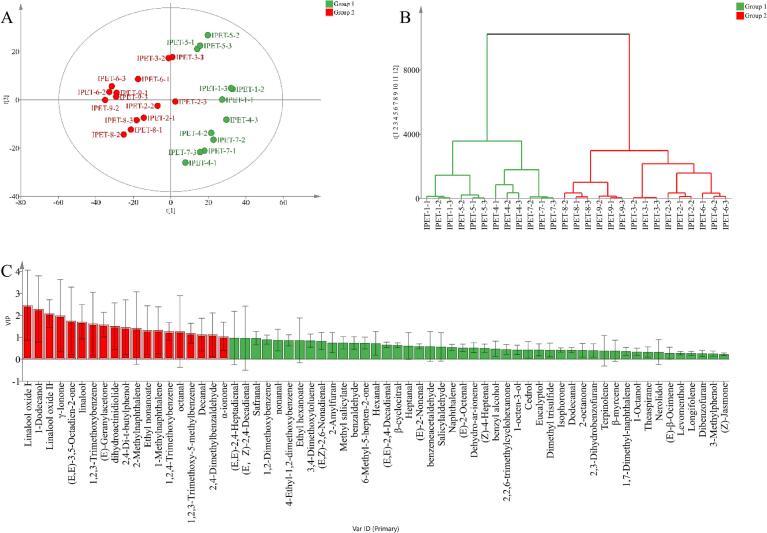
PLS-DA analysis of key differential aroma compounds in IPET with different extraction processes (A: distribution plot; B: Permutation test; C: VIP value).)

PLS-DA is a discriminant analysis method in multivariate data analysis and is often used to handle classification and discrimination problems. This method has been widely used in tea research (Chen et al., 2024). In order to explore the differences in key aroma compounds of IPET with different extraction processes, a PLS-DA model was established. For this study, the Par analysis model was selected. The results showed that the cumulative variances R^2^Y and Q^2^ used for data interpretation were 0.978 and 0.972, respectively. Q^2^ > 0.9 indicates that the model has excellent predictive ability. It can be clearly seen that it is divided into two groups. This result is similar to the PCA result (Fig. 5 A). The permutation test (*n* = 200) revealed intercepts of R^2^ at 0.548 and Q^2^ at −1.01 (Fig. 5 B). A Q^2^ intercept<0 indicated no overfitting in the PLS-DA model.

The VIP value reflects the contribution of a variable to the overall fit and classification ability of the model. The higher the VIP value of a variable, the more important it is in model construction. Generally, variables with VIP values greater than 1 are considered particularly important for the model (Fig. 5 C). In total, there are 19 key odor-active compounds with VIP values greater than 1, in descending order: linalool oxide I (2.47), 1-dodecanol (2.29), linalool oxide II (2.08), γ-ionone (1.98), (*E*,*E*)-3,5-octadien-2-one (1.75), linalool (1.71), 1,2,3-trimethoxybenzene (1.62), (*E*)-geranylacetone (1.57), dihydroactinidiolide (1.51), 2,4-di-t-butylphenol (1.46), 2-methylnaphthalene (1.42), ethyl nonanoate (1.32), 1-methylnaphthalene (1.31), 1,2,4-trimethoxybenzene (1.26), octanal (1.26), 1,2,3-trimethoxy-5-methylbenzene (1.18), decanal (1.13), 2,4-dimethylbenzaldehyde (1.12), and *α*-ionone (1.04). There are significant differences in the content and type of IPET of these compounds in different extraction processes, which is closely related to the extraction rate of odor-active compounds by different extraction factors, resulting in differences in the aroma profile of IPET (Table 2).

**Table 2:** The content of key differential odor-active compounds in IPET.

No.	Compounds	Retention time	CAS	Chemical formula	RI	NIST RI	The contents of aroma compounds (μg/kg)								
							IPET-1	IPET-2	IPET-3	IPET-4	IPET-5	IPET-6	IPET-7	IPET-8	IPET-9
1	linalool oxide I	16.46	5989-33-3	C10H18O2	1063	1074-S	55.73 ± 29.06	#N/A	106.38 ± 4.33	223.06 ± 119.74	58.73 ± 2.61	#N/A	#N/A	202.26 ± 2.73	188.43 ± 13.48
2	1-dodecanol	30.13	112–53-8	C12H26O	1461	1474-S	156.29 ± 7.86	222.17 ± 27.15	356.53 ± 22.74	59 ± 19.29	421.17 ± 19.45	351.7 ± 35.45	28.09 ± 5.19	244.8 ± 7.64	386.06 ± 5.56
3	linalool oxide II	17.06	34,995–77-2	C10H18O2	1079	1086-S	59.33 ± 5.84	310.23 ± 82.18	149.53 ± 5.75	177.38 ± 32.32	87.53 ± 6.11	381.76 ± 111.99	232.84 ± 71.52	368.08 ± 147.53	322.05 ± 100.39
4	γ-ionone	30.59	14,901–07-6	C13H20O	1477	1491-S	83.02 ± 6.48	162.75 ± 24.4	133.34 ± 12.3	123.47 ± 25.61	116.33 ± 15.13	167.97 ± 18.17	#N/A	192.14 ± 9.89	204.23 ± 17.96
5	(*E*,*E*)-3,5-octadien-2-one	16.33	30,086–02-3	C8H12O	1060	1073-S	27.37 ± 3.12	108.59 ± 15.01	81.35 ± 25.32	120.15 ± 99.2	46.34 ± 3.87	120.55 ± 7.94	73.32 ± 2.56	108.17 ± 52.44	127.19 ± 7.87
6	linalool	17.46	78–70-6	C10H18O	1090	1099-S	99.1 ± 10.32	153.42 ± 23.9	202.33 ± 10.13	144.03 ± 32.54	80.77 ± 7.87	273.83 ± 8.05	158.06 ± 5.97	190.66 ± 6.62	239.28 ± 4.54
7	1,2,3-trimethoxybenzene	25.06	634–36-6	C9H12O3	1304	1313-S	512.63 ± 26.13	525.8 ± 20.52	467.93 ± 15.23	474.47 ± 41.22	479.44 ± 8.02	542.86 ± 25.02	494.97 ± 8.53	542.85 ± 14.59	559.4 ± 6.06
8	(*E*)-geranylacetone	29.53	3796-70-1	C13H22O	1442	1453-S	24.37 ± 2.51	43.65 ± 8.76	#N/A	29.62 ± 16.6	30.42 ± 0.22	#N/A	47.91 ± 0.77	70.19 ± 3.49	63.27 ± 3.13
9	dihydroactinidiolide	32.13	17,092–92-1	C11H16O2	1527	1532-S	180.91 ± 7.59	184.94 ± 3.18	161.74 ± 9.57	170.67 ± 13.66	209.96 ± 37.96	203.56 ± 42.06	176.28 ± 8.22	192.17 ± 5.2	219.5 ± 7.94
10	2,4-di-t-butylphenol	31.33	96–76-4	C14H22O	1501	1514-S	49.16 ± 5.26	59.19 ± 17.7	76 ± 7.47	39.84 ± 17.35	39.22 ± 4.73	58.31 ± 3.05	56.86 ± 5.94	62.13 ± 6.35	64.85 ± 2.3
11	2-methylnaphthalene	24.41	91–57-6	C11H10	1286	1297-S	#N/A	13.83 ± 3.84	#N/A	#N/A	#N/A	#N/A	#N/A	#N/A	17.25 ± 0.44
12	ethyl nonanoate	24.39	123–29-5	C11H22O2	1285	1295-S	8.28 ± 0.3	1.65 ± 0.22	#N/A	51.16 ± 24.69	1.54 ± 0.12	#N/A	38.5 ± 3.31	2.13 ± 0.17	#N/A
13	1-methylnaphthalene	24.39	90–12-0	C11H10	1286	1307-S	9.34 ± 0.97	11.08 ± 4	14.17 ± 0.53	11 ± 3.39	9.91 ± 0.43	16.04 ± 1.92	#N/A	17.15 ± 0.01	#N/A
14	1,2,4-trimethoxybenzene	26.99	135–77-3	C9H12O3	1362	1372-S	364.52 ± 14.39	324 ± 16.99	309.83 ± 11.29	336.43 ± 27.89	342.48 ± 3.83	317.98 ± 21.08	353.64 ± 5.29	335.31 ± 9.27	336.48 ± 3.41
15	octanal	13.79	124–13-0	C8H16O	991	1003-S	19.2 ± 1.53	22.21 ± 7.16	19.97 ± 1.1	19.22 ± 5.15	20.37 ± 4.26	#N/A	18.57 ± 3.41	23.89 ± 8.66	#N/A
16	1,2,3-trimethoxy-5-methylbenzene	27.99	6443-69-2	C10H14O3	1393	1407-S	213.99 ± 12.48	206.63 ± 11.65	184.2 ± 5.57	198.23 ± 22.84	198.75 ± 14.61	214.21 ± 13.88	215.08 ± 5.37	229.09 ± 6.82	239.88 ± 4.96
17	decanal	21.26	112–31-2	C10H20O	1195	1206-S	69.56 ± 7.82	67.76 ± 3.51	35.15 ± 7.75	68.43 ± 3.75	53.87 ± 9.01	29.1 ± 2.32	60.92 ± 1.06	45.96 ± 4.91	38.59 ± 2.7
18	2,4-dimethylbenzaldehyde	20.19	15,764–16-6	C9H10O	1166	1182-S	9.04 ± 1.83	13.34 ± 5.36	16.21 ± 0.62	10.78 ± 3.33	10.95 ± 0.63	#N/A	9.72 ± 2.24	18.92 ± 0.25	22.29 ± 0.45
19	*α*-ionone	28.79	127–41-3	C13H20O	1418	1426-S	28.32 ± 1.91	57.63 ± 9.31	55.16 ± 1.45	46.94 ± 10.89	42.97 ± 3.27	73.07 ± 10.19	66.27 ± 6.04	82.82 ± 6.74	79.06 ± 1.24

**Fig. 6:**
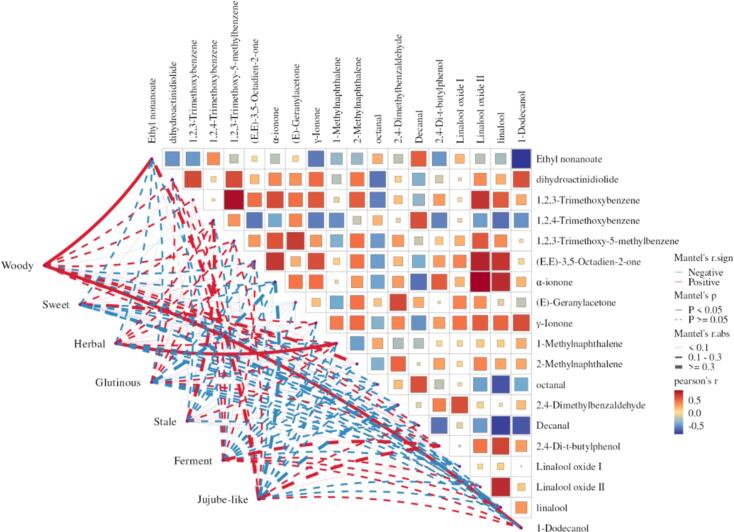
Pearson correlation analysis between key differential compounds and aroma attributes in IPET with different extraction processes.

To further explore the relationship between key differential compounds and aroma characteristics in IPET with different extraction processes. Pearson correlation analysis was conducted between 19 key differential compounds and seven aroma characteristic intensities including woody aroma, sweet aroma, medicinal aroma, glutinous aroma, aged aroma, fermented odor, and jujube aroma (Fig. 6). The results showed that woody aroma was positively correlated with ethyl nonanoate (*r* = 0.63, *p* = 0.004) and 1-dodecanol (*r* = 0.53, *p* = 0.009). Medicinal aroma was positively correlated with 1-methylnaphthalene (*r* = 0.48, *p* = 0.036).

## 4 Conclusion

In this study, different extraction temperatures, extraction time, and tea-to-water ratios were taken as parameters, and PET was used as original material, IPET prepared by vacuum freeze drying was further taken as the research object. Through sensory evaluation, QDA, and HS-SPME-GC × GC-TOF/MS, the effects of extraction factors on the sensory quality and aroma profile of IPET were explored. The sensory evaluation results showed that IPET-2 and IPET-8 had relatively high total scores, indicating their superiority in aroma, soup color, and taste compared to others. The orthogonal test analysis revealed that the factors influencing the quality of IPET in descending order of importance were extraction temperature > extraction time > tea-water ratio. The optimal process conditions for IPETs were determined to be a tea-water ratio of 1:8, an extraction temperature of 75 °C, and an extraction time of 60 min. QDA demonstrated distinct grid shapes surrounded by the intensity values of aroma attributes for IPETs with different extraction processes, each with its own characteristic aroma. For instance, IPET-5 was prominent in herbal aroma, IPET-2 in sweet aroma and stale aroma, and IPET-4 in jujube-like aroma. A total of 235 volatile compounds were detected in IPET samples by HS-SPME-GC × GC-TOF/MS, which were classified into various categories. Further analysis by ROAV identified 65 key odor-active compounds with ROAV>1 in IPET samples. PLS-DA based on the concentrations of key odor-active compounds provided clear discrimination with satisfactory model parameters and 19 key differential odor-active compounds with VIP > 1 were identified. Pearson correlation analysis of key differential odor-active compounds and aroma attributes showed that positive correlations between woody aroma and ethyl nonanoate and 1-dodecanol, and between herbal aroma and 1-methylnaphthalene. In conclusion, this study provides valuable theoretical support for the production of high-quality IPET. The findings on optimal extraction processes and understanding of aroma profiles can guide manufacturers in producing IPET with excellent sensory qualities and distinct aromas.

## Sensory Evaluation Ethics information and Sensory Evaluation Consent Information

Tea belongs to the category of food beverage, and its sensory evaluation does not require ethical permission. Therefore, no human ethics committee or formal documentation process was available, but appropriate protocols for protecting the rights and privacy of all participants were utilized during the study, e.g., no coercion to participate, full disclosure of the study requirements and risks, written or verbal consent of participants, no release of participant data without participants' knowledge, and the ability to withdraw from the study at any time. In addition, the publication of all sensory research data was subject to the consent of all participants and used their information.

## CRediT authorship contribution statement

**Guohe Chen:** Writing – original draft, Project administration, Methodology, Investigation. **Yajie Xue:** Writing – review & editing, Project administration, Methodology. **Guangmei Zhu:** Writing – review & editing, Data curation. **He Xie:** Methodology, Investigation. **Jing Zhang:** Software, Resources. **Chuyi He:** Formal analysis. **Jianan Huang:** Visualization, Validation, Supervision, Funding acquisition. **Zhonghua Liu:** Visualization, Validation, Supervision, Funding acquisition. **Chao Wang:** Visualization, Validation, Supervision, Funding acquisition.

## Declaration of competing interest

The authors declare that they have no known competing financial interests or personal relationships that could have appeared to influence the work reported in this paper.

## Data Availability

The data that has been used is confidential.

## Appendix A Supplementary data

Supplementary material Image 1
