# Supplementary material for: Characterization of the influence of extraction factors on instant Pu-erh tea: Focusing on changes in sensory quality and aroma profile
Source: Food Chem X. 2024 Oct 23;24:101925. doi: 10.1016/j.fochx.2024.101925 (PMC11599999; doi:10.1016/j.fochx.2024.101925)
Supplement: Supplementary file 1 — Supplementary material [file mmc1.docx]

**Supplementary material**

Table S1 Orthogonal test scheme

|  | extraction factors | | |
| --- | --- | --- | --- |
|  | solid-liquid ratio | extraction temperature | extraction time |
| IPET-1 | 1:6 | 50 | 40 |
| IPET-2 | 1:6 | 75 | 60 |
| IPET-3 | 1:6 | 100 | 80 |
| IPET-4 | 1:8 | 50 | 80 |
| IPET-5 | 1:8 | 75 | 40 |
| IPET-6 | 1:8 | 100 | 60 |
| IPET-7 | 1:10 | 50 | 60 |
| IPET-8 | 1:10 | 75 | 80 |
| IPET-9 | 1:10 | 100 | 40 |

Table S2 Orthogonal test factors and level table

| Levels | Extraction factors | | |
| --- | --- | --- | --- |
|  | A  tea-to-water ratio | B  extraction temperature /℃ | C  extraction time /min |
| 1 | 1:6 | 50 | 40 |
| 2 | 1:8 | 75 | 60 |
| 3 | 1:10 | 100 | 80 |

Table S3 Range analysis of orthogonal test of IPET

| Test No. | A | B | C | Sensory evaluation score |
| --- | --- | --- | --- | --- |
| 1 | 1 | 1 | 1 | 83.5 |
| 2 | 1 | 2 | 2 | 91.7 |
| 3 | 1 | 3 | 3 | 88.1 |
| 4 | 2 | 1 | 3 | 86.4 |
| 5 | 2 | 2 | 1 | 89.7 |
| 6 | 2 | 3 | 2 | 89.1 |
| 7 | 3 | 1 | 2 | 85.2 |
| 8 | 3 | 2 | 3 | 90.9 |
| 9 | 3 | 3 | 1 | 86.3 |
| K1 | 263.30 | 255.10 | 259.50 |  |
| K2 | 265.20 | 272.30 | 266.00 |  |
| K3 | 262.40 | 263.50 | 265.40 |  |
| k1 | 87.77 | 85.03 | 86.50 |  |
| k2 | 88.40 | 90.77 | 88.67 |  |
| k3 | 87.47 | 87.63 | 88.47 |  |
| Range | 0.93 | 5.74 | 2.17 |  |
| Primary and secondary factors | B＞C＞A | | |  |
| Optimal combination | A_2_B_2_C_2_ | | |  |

**Table S4 Sensory evaluation of IPET under optimal conditions.**

| Sample | Aroma（30%） | | Soup color（30%） | | Taste（40%） | | Total score | Yield（%） |
| --- | --- | --- | --- | --- | --- | --- | --- | --- |
|  | Comment | Score | Comment | Score | Comment | Score |  |  |
| IPET-10 | Pure stale aroma with sweet aroma | 93 | Dark reddish-brown | 91 | Mellow, smooth, sweet, and with sweet aftertaste | 93 | 92.4 | 18.84 |

Table S5 The contents of volatile compounds in IPETs

| No. | Compounds | Retention time | CAS^a^ | Category | Chemical formula | RI | NIST RI | The contents of volatile compounds in IPETs (ug/kg) ^b^ | | | | | | | | |
| --- | --- | --- | --- | --- | --- | --- | --- | --- | --- | --- | --- | --- | --- | --- | --- | --- |
|  |  |  |  |  |  |  |  | IPET-1 | IPET-2 | IPET-3 | IPET-4 | IPET-5 | IPET-6 | IPET-7 | IPET-8 | IPET-9 |
| 1 | Ethyl hexanoate | 13.66 | 123-66-0 | Esters | C8H16O2 | 988 | 999-S | 2.12±0.18 | #N/A | #N/A | 20.32±9.93 | #N/A | #N/A | 9.02±0.54 | #N/A | #N/A |
| 2 | Octanoic acid,ethyl ester | 20.99 | 106-32-1 | Esters | C10H20O2 | 1187 | 1196-S | 8.09±1.22 | #N/A | #N/A | 34.56±18.91 | 2.33±1.28 | #N/A | 19.46±1.03 | 3.14±0.44 | #N/A |
| 3 | Methyl salicylate | 20.93 | 119-36-8 | Esters | C8H8O3 | 1186 | 1192-S | 33.28±2.7 | 29.28±3.51 | 22.1±0.76 | 51.15±10.95 | 14.96±0.3 | 23.23±2.1 | 53.92±1.64 | 35.54±3.27 | 26.01±1.11 |
| 4 | Nonanoic acid, methyl ester | 21.93 | 1731-84-6 | Esters | C10H20O2 | 1214 | 1223-S | 1.49±0.19 | #N/A | #N/A | 14.46±8.23 | #N/A | #N/A | 9.89±1.21 | #N/A | #N/A |
| 5 | Ethyl nonanoate | 24.39 | 123-29-5 | Esters | C11H22O2 | 1285 | 1295-S | 8.28±0.3 | 1.65±0.22 | #N/A | 51.16±24.69 | 1.54±0.12 | #N/A | 38.5±3.31 | 2.13±0.17 | #N/A |
| 6 | Ethanol, 2-(2-butoxyethoxy)-, acetate | 26.73 | 124-17-4 | Esters | C10H20O4 | 1355 | 1366-S | 8.68±4.77 | 5.14±1.96 | 5.16±1.73 | 4.98±3.33 | #N/A | #N/A | 5.65±0.44 | 5.73±0.48 | 4.11±0.57 |
| 7 | Ethyl trans-4-decenoate | 27.19 | 76649-16-6 | Esters | C12H22O2 | 1368 | 1347-S | 2.73±0.71 | #N/A | 3.49±0.75 | 2.18±0.39 | #N/A | #N/A | #N/A | #N/A | #N/A |
| 8 | dihydroactinidiolide | 32.13 | 17092-92-1 | Esters | C11H16O2 | 1527 | 1532-S | 180.91±7.59 | 184.94±3.18 | 161.74±9.57 | 170.67±13.66 | 209.96±37.96 | 203.56±42.06 | 176.28±8.22 | 192.17±5.2 | 219.5±7.94 |
| 9 | Dodecanoic acid, ethyl ester | 33.73 | 106-33-2 | Esters | C14H28O2 | 1582 | 1594-S | 4.27±0.27 | #N/A | #N/A | 3.73±0.97 | #N/A | #N/A | 3.69±0.35 | #N/A | #N/A |
| 10 | 2,2,4-Trimethyl-1,3-pentanediol diisobutyrate | 33.86 | 6846-50-0 | Esters | C16H30O4 | 1587 | 1588-S | 5.11±0.6 | 6.43±0.73 | 4.36±1.86 | #N/A | #N/A | 3.23±1.42 | 3.61±2.81 | 4.02±2.16 | 6.74±0.92 |
| 11 | Propanoic acid, 2-methyl-, 3-hydroxy-2,2,4-trimethylpentyl ester | 27.07 | 77-68-9 | Esters | C12H24O3 | 1365 | 1374-S | #N/A | 2.61±0.08 | 1.86±0.21 | 1.67±0.1 | #N/A | 2.8±0.44 | 2.42±0.56 | 2.93±0.36 | 2.9±0.33 |
| 12 | 4-(2,6,6-Trimethylcyclohexa-1,3-dienyl)but-3-en-2-one | 27.34 | 1203-08-3 | Esters | C13H18O | 1373 | 1485-S | #N/A | 50.33±32.49 | 25.01±0.89 | #N/A | #N/A | 34.63±4.93 | #N/A | 20.92±15.29 | #N/A |
| 13 | Methyl hexanoate | 10.94 | 106-70-7 | Esters | C7H14O2 | 913 | 925-S | #N/A | #N/A | #N/A | 1.76±0.78 | #N/A | #N/A | 0.87±0 | #N/A | #N/A |
| 14 | Heptanoic acid, methyl ester | 14.61 | 106-73-0 | Esters | C8H16O2 | 1013 | 1023-S | #N/A | #N/A | #N/A | 3.1±1.62 | #N/A | #N/A | 2.26±0.71 | #N/A | #N/A |
| 15 | Octanoic acid, methyl ester | 18.34 | 111-11-5 | Esters | C9H18O2 | 1113 | 1126-S | #N/A | #N/A | #N/A | 8.75±4.03 | #N/A | #N/A | 4.9±0.46 | #N/A | #N/A |
| 16 | Methyl caprate | 25.34 | 110-42-9 | Esters | C11H22O2 | 1312 | 1326-S | #N/A | #N/A | #N/A | 3.87±1.87 | #N/A | #N/A | 3±0.28 | #N/A | #N/A |
| 17 | Dodecanoic acid, methyl ester | 31.67 | 111-82-0 | Esters | C13H26O2 | 1512 | 1526-S | #N/A | #N/A | #N/A | 1.38±0.4 | #N/A | #N/A | #N/A | #N/A | #N/A |
| 18 | 2,6-Octadienoic acid, 3,7-dimethyl-, methyl ester, (Z)- | 25.33 | 1862-61-9 | Esters | C11H18O2 | 1313 | 1298-N | #N/A | #N/A | #N/A | #N/A | #N/A | 1.87±0.01 | #N/A | #N/A | #N/A |
| 19 | trans-Geranic acid methyl ester | 25.33 | 1189-09-9 | Esters | C11H18O2 | 1313 | 1322-S | #N/A | #N/A | #N/A | #N/A | #N/A | #N/A | #N/A | #N/A | 2.12±0.13 |
| 20 | 1,2-Dimethoxybenzene | 19.13 | 91-16-7 | Heterooxygen compounds | C8H10O2 | 1137 | 1148-S | 144.5±11.33 | 205.87±10.19 | 162.93±4 | 154.92±20.8 | 149.3±6.64 | 212.99±15.32 | 170.78±5.1 | 211.58±0.97 | 209.89±1.52 |
| 21 | Benzene, 1-ethenyl-4-methoxy- | 19.39 | 637-69-4 | Heterooxygen compounds | C9H10O | 1144 | 1156-S | 1.79±0.11 | 7.01±1.64 | 17.6±0.93 | 1.68±0.06 | 4.6±0.49 | 21.32±1.57 | 1.81±0.89 | 8.27±0.69 | 21.13±0.57 |
| 22 | 3,4-Dimethoxytoluene | 22.46 | 494-99-5 | Heterooxygen compounds | C9H12O2 | 1230 | 1233-S | 106.95±9.17 | 142.01±13.27 | 117.21±2.16 | 122.4±21.08 | 114.6±9.95 | 143.85±10.68 | 133.77±3.93 | 167.46±3.32 | 158.55±2.34 |
| 23 | 3,5-Dimethoxytoluene | 23.46 | 4179-19-5 | Heterooxygen compounds | C9H12O2 | 1259 | 1274-S | 3.32±0.73 | 7.55±0.66 | 5.91±2.54 | 5.85±1.83 | 5.66±0.75 | 9.31±2.62 | 6.95±0.21 | 11.13±1.76 | 12.22±1.6 |
| 24 | 1,2,3-Trimethoxybenzene | 25.06 | 634-36-6 | Heterooxygen compounds | C9H12O3 | 1304 | 1313-S | 512.63±26.13 | 525.8±20.52 | 467.93±15.23 | 474.47±41.22 | 479.44±8.02 | 542.86±25.02 | 494.97±8.53 | 542.85±14.59 | 559.4±6.06 |
| 25 | 4-Ethyl-1,2-dimethoxybenzene | 25.33 | 5888-51-7 | Heterooxygen compounds | C10H14O2 | 1313 | 1320-S | 133.96±9.3 | 168.56±18.02 | 138.12±4 | 139.59±23.33 | 136.63±10.06 | 178.37±14.93 | 166.91±7.07 | 189.73±7.12 | 185.57±2.95 |
| 26 | 1,2,4-Trimethoxybenzene | 26.99 | 135-77-3 | Heterooxygen compounds | C9H12O3 | 1362 | 1372-S | 364.52±14.39 | 324±16.99 | 309.83±11.29 | 336.43±27.89 | 342.48±3.83 | 317.98±21.08 | 353.64±5.29 | 335.31±9.27 | 336.48±3.41 |
| 27 | 1,2,3-Trimethoxy-5-methylbenzene | 27.99 | 6443-69-2 | Heterooxygen compounds | C10H14O3 | 1393 | 1407-S | 213.99±12.48 | 206.63±11.65 | 184.2±5.57 | 198.23±22.84 | 198.75±14.61 | 214.21±13.88 | 215.08±5.37 | 229.09±6.82 | 239.88±4.96 |
| 28 | Methyl isoeugenol | 30.86 | 93-16-3 | Heterooxygen compounds | C11H14O2 | 1486 | 1492-S | 5.02±0.54 | 3.37±0.29 | 4.36±2.04 | 3.68±0.83 | 3.99±1.01 | 56.48±81.73 | 7.56±2.98 | 5.16±2.49 | 6.12±2.29 |
| 29 | Elemicin | 32.66 | 487-11-6 | Heterooxygen compounds | C12H16O3 | 1547 | 1554-S | 10.65±0.44 | 8.84±4.01 | 9.76±0.65 | 11.9±0.51 | 10.78±3.52 | 8.82±4.53 | 9.61±0.93 | 11.33±0.58 | 15.42±1.5 |
| 30 | 4-Acetyl-1-methylcyclohexene | 15.61 | 1530612 | Heterooxygen compounds | C9H14O | 1040 | 1137-S | #N/A | 4.59±0.62 | #N/A | #N/A | #N/A | #N/A | 1.75±0.89 | 5.34±2.54 | #N/A |
| 31 | 1,3-Dimethoxybenzene | 19.94 | 151-10-0 | Heterooxygen compounds | C8H10O2 | 1159 | 1168-S | #N/A | 3.94±0.97 | 3.05±0.15 | 3.42±2.2 | 4.77±2.25 | #N/A | #N/A | 4.67±0.04 | 4.09±1.2 |
| 32 | Theaspirane | 25.21 | 36431-72-8 | Heterooxygen compounds | C13H22O | 1309 | 1302-S | #N/A | 1.73±0.07 | 2.2±0.62 | 1.84±0.03 | #N/A | 2.11±0.82 | 3.19±0.92 | 2.61±0.92 | 2.05±0.05 |
| 33 | Bicyclo[2.2.1]heptan-2-one, 1,7,7-trimethyl-, (1S)- | 19.19 | 464-48-2 | Heterooxygen compounds | C10H16O | 1138 | 1145-S | #N/A | #N/A | #N/A | #N/A | #N/A | #N/A | 9.17±0.03 | 14.32±0.51 | #N/A |
| 34 | Bicyclo[3.1.0]hexan-2-ol, 2-methyl-5-(1-methylethyl)-, (1α,2β,5α)- | 17.46 | 15537-55-0 | Heterooxygen compounds | C10H18O | 1090 | 1070-S | #N/A | #N/A | #N/A | #N/A | #N/A | #N/A | #N/A | 6.14±0.29 | #N/A |
| 35 | L-Borneol | 19.93 | 464-45-9 | Heterooxygen compounds | C10H18O | 1158 | 1170-S | #N/A | #N/A | #N/A | #N/A | #N/A | #N/A | #N/A | 14.33±0.66 | #N/A |
| 36 | Estragole | 21.06 | 140-67-0 | Heterooxygen compounds | C10H12O | 1190 | 1196-S | #N/A | #N/A | #N/A | #N/A | #N/A | #N/A | #N/A | 5.32±0.47 | #N/A |
| 37 | Anethole | 24.13 | 104-46-1 | Heterooxygen compounds | C10H12O | 1278 | 1287-S | #N/A | #N/A | #N/A | #N/A | #N/A | #N/A | #N/A | 19.37±1.77 | #N/A |
| 38 | 2-Heptanone | 9.73 | 110-43-0 | Ketones | C7H14O | 877 | 891-S | 3.98±0.48 | 8.73±3.96 | 6.22±2.06 | 5.27±1.59 | 3.84±1.02 | 8.32±1.97 | 5.76±1.4 | 9.28±0.71 | 7.16±0.2 |
| 39 | 2,3-Octanedione | 131259 | 585-25-1 | Ketones | C8H14O2 | 973 | 984-S | 4.94±0.49 | 7.76±2.73 | 12.3±0.66 | 3.83±0.83 | 5.06±0.46 | 11.82±1.3 | 4.46±0.52 | 8.7±0.98 | 12.86±1.23 |
| 40 | 6-Methyl-5-hepten-2-one | 13.19 | 110-93-0 | Ketones | C8H14O | 975 | 986-S | 7.08±0.79 | 28.12±0.44 | 13.94±0.79 | 18.01±3.71 | 10.43±1.71 | 26.75±5.31 | 17.55±0.62 | 32.6±1.24 | 22.71±0.92 |
| 41 | 2-octanone | 13.39 | 111-13-7 | Ketones | C8H16O | 980 | 991-S | 3.12±0.12 | 7.18±3.25 | 4.43±1.42 | 5.42±4.2 | #N/A | 7.72±2.72 | 5.25±1 | 10.3±0.15 | 8.38±1.07 |
| 42 | 2,2,6-trimethylcyclohexanone | 15.06 | 2408-37-9 | Ketones | C9H16O | 1026 | 1036-S | 7.45±0.86 | 18.61±6.33 | 12.59±1.21 | 13.81±4.48 | 7.14±1.38 | 18.9±3.61 | 16.43±0.47 | 25.49±1.13 | 22.47±2.09 |
| 43 | 3,6,6-TRIMETHYL-CYCLOHEX-2-ENONE | 15.99 | 23438-77-9 | Ketones | C9H14O | 1051 | 1152-S | 7.27±0.96 | 25.41±0.26 | 15.06±0.44 | 15.67±4.73 | 9.38±0.2 | #N/A | 18.65±0.59 | #N/A | 23.32±1.15 |
| 44 | Acetophenone | 16.19 | 98-86-2 | Ketones | C8H8O | 1057 | 1066-S | 9.84±0.13 | 31.69±1.51 | 24.44±1.42 | 20.06±2.68 | 16.27±2.31 | 38.59±1.08 | 19.43±0.31 | 35.61±1.42 | 37.81±1.74 |
| 45 | (E,E)-3,5-Octadien-2-one | 16.33 | 30086-02-3 | Ketones | C8H12O | 1060 | 1073-S | 27.37±3.12 | 108.59±15.01 | 81.35±25.32 | 120.15±99.2 | 46.34±3.87 | 120.55±7.94 | 73.32±2.56 | 108.17±52.44 | 127.19±7.87 |
| 46 | 6-Methyl-3,5-heptadiene-2-one | 17.59 | 1604-28-0 | Ketones | C8H12O | 1094 | 1107-S | 5.7±1.14 | 17.67±1.85 | 13.37±1.69 | 11.3±4.96 | 9.46±1.76 | 20.46±1.78 | 11.93±2.03 | 23.96±4.77 | 24.34±1.8 |
| 47 | 2H-Pyran-3(4H)-one, 6-ethenyldihydro-2,2,6-trimethyl- | 17.73 | 33933-72-1 | Ketones | C10H16O2 | 1097 | 1108-S | 18.28±1.81 | 67.15±6.62 | 43.74±1.41 | 50.17±11.83 | 29.13±4.68 | 69.71±3.78 | 52.82±1.3 | 84.28±1.81 | 70.29±2.2 |
| 48 | Isophorone | 18.26 | 78-59-1 | Ketones | C9H14O | 1112 | 1123-S | 5.07±0.4 | 21.63±1.81 | 17.42±0.65 | 12.81±3.46 | 8.9±0.4 | 27.8±1.12 | 12.88±1.11 | 25.99±1.33 | 26.57±1.39 |
| 49 | 3-Nonen-2-one | 18.86 | 14309-57-0 | Ketones | C9H16O | 1128 | 1142-S | 3.15±0.31 | 11.1±2.57 | #N/A | 6.25±3.21 | 5.4±0.63 | #N/A | 6.8±1.16 | 14.65±0.37 | 19.12±1.86 |
| 50 | (R,S)-5-Ethyl-6-methyl-3E-hepten-2-one | 19.13 | 57283-79-1 | Ketones | C10H18O | 1136 | 1144-S | 5.63±0.55 | 16.29±3.58 | 12.63±0.94 | 10.97±4.02 | 8.46±0.67 | 20.07±1.16 | 13.35±0.26 | 24.14±0.84 | 22.12±0.75 |
| 51 | Camphor | 19.19 | 76-22-2 | Ketones | C10H16O | 1138 | 1145-S | 3.68±0.42 | 10.15±1.57 | 7.32±0.51 | #N/A | 5.15±0.57 | 11.89±1.4 | #N/A | #N/A | 11.57±0.2 |
| 52 | Dehydromevalonic lactone | 19.79 | 2381-87-5 | Ketones | C6H8O2 | 1154 | 1169-S | 5.23±0.47 | 4.94±0.36 | 3.23±0.42 | 4.41±0.82 | 3.92±1.04 | 5.9±4.01 | 3.78±1.14 | 3.74±1.1 | 3.77±0.57 |
| 53 | 1-Propanone, 1-phenyl- | 19.86 | 93-55-0 | Ketones | C9H10O | 1157 | 1176-S | 8.69±1.12 | 24.82±0.32 | 21.41±2.22 | 14.66±1.93 | 13.68±1.44 | 35.19±2.07 | 14.11±1.31 | 30.06±2.27 | 31.02±2.52 |
| 54 | 4-Methyleneisophorone | 21.86 | 20548-00-9 | Ketones | C10H14O | 1213 | 1242-S | 7±0.01 | 17.93±2.19 | 15.35±0.3 | 12.96±2.92 | 10.65±0.46 | 22.83±2.18 | 13.65±0.29 | 29.23±4.07 | 24.89±3.3 |
| 55 | 1H-Pyrrole-2,5-dione, 3-ethyl-4-methyl- | 22.13 | 20189-42-8 | Ketones | C7H9NO2 | 1221 | 1239-S | 6.22±0.15 | 4.35±1.22 | 3.35±1.17 | 5.48±1.14 | 5.62±1.65 | #N/A | 4.11±1.45 | 5.03±0.56 | 5.3±0.32 |
| 56 | 2-Undecanone | 24.33 | 112-12-9 | Ketones | C11H22O | 1283 | 1294-S | 4.09±0.4 | 4.39±1.57 | 5.38±0.51 | #N/A | 3.82±0.26 | 6.16±0.35 | #N/A | 7.41±0.97 | 7.97±1.48 |
| 57 | (Z)-Jasmone | 27.86 | 488-10-8 | Ketones | C11H16O | 1389 | 1395-S | 1.61±0.11 | 5.35±0.62 | 4.04±0.99 | 2.56±0.37 | 3.15±0.61 | 7.05±1.47 | 3.85±1.1 | 6.9±0.48 | 7.84±0.06 |
| 58 | 2-Undecanone, 6,10-dimethyl- | 27.99 | 1604-34-8 | Ketones | C13H26O | 1392 | 1408-S | 2.37±0.07 | 2.59±1.02 | 4.09±0.08 | 1.67±0.07 | 2.39±0.08 | 4.34±0.6 | 1.58±0.06 | 3.08±0.23 | 4.32±0.28 |
| 59 | 6-Methyl-6-(5-methylfuran-2-yl)heptan-2-one | 28.66 | 50464-95-4 | Ketones | C13H20O2 | 1414 | 1433-S | 4.48±0.37 | 10.18±1.37 | 11.58±1.65 | 7.96±1.88 | 9.9±2.12 | 19.13±4.64 | #N/A | 20.38±2.37 | 22.65±0.51 |
| 60 | α-ionone | 28.79 | 127-41-3 | Ketones | C13H20O | 1418 | 1426-S | 28.32±1.91 | 57.63±9.31 | 55.16±1.45 | 46.94±10.89 | 42.97±3.27 | 73.07±10.19 | 66.27±6.04 | 82.82±6.74 | 79.06±1.24 |
| 61 | (E)-Geranylacetone | 29.53 | 3796-70-1 | Ketones | C13H22O | 1442 | 1453-S | 24.37±2.51 | 43.65±8.76 | #N/A | 29.62±16.6 | 30.42±0.22 | #N/A | 47.91±0.77 | 70.19±3.49 | 63.27±3.13 |
| 62 | γ-Ionone | 30.59 | 14901-07-6 | Ketones | C13H20O | 1477 | 1491-S | 83.02±6.48 | 162.75±24.4 | 133.34±12.3 | 123.47±25.61 | 116.33±15.13 | 167.97±18.17 | #N/A | 192.14±9.89 | 204.23±17.96 |
| 63 | 2-Heptanone, 6-methyl- | 12.07 | 928-68-7 | Ketones | C8H16O | 944 | 956-S | #N/A | 1.55±0.03 | #N/A | #N/A | #N/A | #N/A | #N/A | 1.44±0.15 | #N/A |
| 64 | 3-Cyclohexen-1-one, 3,5,5-trimethyl- | 16.74 | 471-01-2 | Ketones | C9H14O | 1070 | 1044-S | #N/A | 5.14±0.31 | 3±0.18 | 3.42±0.71 | 1.69±0.7 | 4.53±0.27 | 3.5±0.15 | 6.29±0.63 | 4.55±0.3 |
| 65 | Bicyclo[3.1.0]hexan-2-one, 5-(1-methylethyl)- | 19.67 | 513-20-2 | Ketones | C9H14O | 1151 | 1156-S | #N/A | 2.12±0.06 | #N/A | #N/A | #N/A | 1.89±0.17 | #N/A | 3.96±1.56 | #N/A |
| 66 | 1,4-Cyclohexanedione, 2,2,6-trimethyl- | 19.94 | 20547-99-3 | Ketones | C9H14O2 | 1159 | 1183-S | #N/A | 3.19±0.5 | 2.23±0.14 | 2.3±0.85 | #N/A | 3.67±1.41 | 1.95±0.01 | 4.3±0.23 | 4.38±0.49 |
| 67 | 1-(4-Methylphenyl)ethanone | 20.14 | 122-00-9 | Ketones | C9H10O | 1164 | 1183-S | #N/A | 3.33±0.85 | #N/A | #N/A | #N/A | #N/A | #N/A | 4.77±0.26 | 5.45±0.17 |
| 68 | 2-Cyclohexen-1-one, 5-methyl-2-(1-methylethyl)- | 22.87 | 5113-66-6 | Ketones | C10H16O | 1241 | 1251-S | #N/A | 4.7±0.32 | 2.41±0.1 | 3.52±1.73 | 2.37±0.63 | 3.47±0.25 | 3.75±0.33 | 5.77±1.52 | 5.61±2.15 |
| 69 | 2-Butanone, 4-(2,6,6-trimethyl-2-cyclohexen-1-yl)- | 28.47 | 31499-72-6 | Ketones | C13H22O | 1407 | 1406-S | #N/A | 1.93±0.01 | 1.83±0.07 | 1.5±0.46 | 1.45±0.23 | 2.51±0.4 | 2.35±0.38 | 2.54±0.18 | 2.28±0.04 |
| 70 | 3-Octen-2-one, (E)- | 15.22 | 18402-82-9 | Ketones | C8H14O | 1029 | 1035-S | #N/A | #N/A | 3.25±0.4 | #N/A | #N/A | #N/A | #N/A | #N/A | #N/A |
| 71 | trans-3-Nonen-2-one | 18.88 | 18402-83-0 | Ketones | C9H16O | 1128 | 1144-S | #N/A | #N/A | 12.73±0.95 | #N/A | #N/A | 21.15±0.72 | #N/A | #N/A | #N/A |
| 72 | Oxopholone | 19.08 | 1125-21-9 | Ketones | C9H12O2 | 1135 | 1145-S | #N/A | #N/A | 9.4±0.53 | 7.11±1.94 | 5.22±0.49 | 16.53±0.34 | 6.81±0.15 | 14.4±0.28 | 15.9±0.49 |
| 73 | 1-(2-methylphenyl)ethanone | 20.14 | 577-16-2 | Ketones | C9H10O | 1164 | 1173-S | #N/A | #N/A | 4.26±0.03 | #N/A | #N/A | #N/A | #N/A | #N/A | #N/A |
| 74 | 2-Decanone | 20.82 | 693-54-9 | Ketones | C10H20O | 1182 | 1193-S | #N/A | #N/A | 6.94±0.58 | #N/A | #N/A | 7.63±2.04 | 2.97±0.09 | #N/A | 9.67±0.63 |
| 75 | D-Verbenone | 21.55 | 18309-32-5 | Ketones | C10H14O | 1203 | 1228-S | #N/A | #N/A | 2.59±0.29 | #N/A | #N/A | #N/A | #N/A | 4.48±0.62 | #N/A |
| 76 | β-Damascenone | 27.48 | 23726-93-4 | Ketones | C13H18O | 1377 | 1386-S | #N/A | #N/A | 2.89±0.95 | #N/A | #N/A | 3.36±1.26 | #N/A | 1.33±0.09 | 3.02±1.45 |
| 77 | Geranyl acetone | 29.55 | 689-67-8 | Ketones | C13H22O | 1442 | 1452-S | #N/A | #N/A | 35.38±2.22 | 10±5.96 | #N/A | 48.11±7.3 | #N/A | #N/A | #N/A |
| 78 | 3-Methylacetophenone | 20.14 | 585-74-0 | Ketones | C9H10O | 1164 | 1182-S | #N/A | #N/A | #N/A | 3.01±0.25 | 2.73±0.16 | #N/A | 2.35±0.28 | #N/A | #N/A |
| 79 | Benzophenone | 34.87 | 119-61-9 | Ketones | C13H10O | 1625 | 1635-S | #N/A | #N/A | #N/A | #N/A | 2.16±0.23 | 2.29±0.24 | 1.63±0.11 | 1.86±0.2 | 2.25±0.02 |
| 80 | 3-Hepten-2-one | 11.39 | 1119-44-4 | Ketones | C7H12O | 926 | 923-N | #N/A | #N/A | #N/A | #N/A | #N/A | 1.52±0.04 | #N/A | #N/A | #N/A |
| 81 | 2-Cyclohexen-1-one, 3-methyl- | 15.99 | 1193-18-6 | Ketones | C7H10O | 1051 | 1075-S | #N/A | #N/A | #N/A | #N/A | #N/A | 23.75±2.42 | #N/A | 29.67±0.86 | #N/A |
| 82 | Bicyclo[3.1.1]hept-3-en-2-one, 4,6,6-trimethyl- | 21.53 | 80-57-9 | Ketones | C10H14O | 1203 | 1184-N | #N/A | #N/A | #N/A | #N/A | #N/A | 4±0.44 | 1.81±0.06 | 5.09±0.45 | 3.58±0.45 |
| 83 | β-ionone | 30.59 | 79-77-6 | Ketones | C13H20O | 1477 | 1486-S | #N/A | #N/A | #N/A | #N/A | #N/A | #N/A | 158.78±3.15 | #N/A | #N/A |
| 84 | 1H-Inden-1-one, 2,3-dihydro-3-methyl- | 25.26 | 6072-57-7 | Ketones | C10H10O | 1310 | 1332-S | #N/A | #N/A | #N/A | #N/A | #N/A | #N/A | #N/A | 5.18±0.14 | #N/A |
| 85 | 1,3-Cyclopentadiene, 5,5-dimethyl-1-ethyl- | 8.19 | 94167-80-3 | Hydrocarbons | C9H14 | 828 | 856-S | 6.3±0.47 | 4.11±1.66 | #N/A | 4.68±1.7 | 4.23±0.16 | 4.94±0.23 | 5.75±0.28 | 4.62±0.16 | 5.16±0.25 |
| 86 | 1,3-cis,5-cis-Octatriene | 9.33 | 40087-62-5 | Hydrocarbons | C8H12 | 864 | 879-S | 23.91±2.06 | 3.66±0.06 | 1.86±0.43 | 20.23±9.45 | #N/A | 1.46±0.01 | 29.63±1.45 | 2.89±0.3 | 1.75±0.11 |
| 87 | Styrene | 9.73 | 100-42-5 | Hydrocarbons | C8H8 | 877 | 893-S | 1.62±0.14 | 4.72±3.57 | 2.57±0.17 | 6.44±3.93 | 4.1±2.07 | 8.94±4.12 | 7.56±1.17 | 9.09±2.09 | 3.58±0.19 |
| 88 | β-myrcene | 13.39 | 123-35-3 | Hydrocarbons | C10H16 | 980 | 991-S | 1.43±0.09 | 1.16±0.01 | 1.33±0.07 | #N/A | #N/A | 1.1±0.18 | 1.12±0.17 | 1.01±0.05 | 1.07±0.04 |
| 89 | Benzene, 1-ethyl-3-methyl- | 13.46 | 620-14-4 | Hydrocarbons | C9H12 | 982 | 957-S | 1.82±0.22 | #N/A | 4.07±2.77 | 2.44±0.06 | #N/A | 2.9±0.22 | #N/A | 3.3±0.03 | 3.21±0.31 |
| 90 | (E)-β-Ocimene | 15.13 | 3779-61-1 | Hydrocarbons | C10H16 | 1027 | 1049-S | 1.61±0.17 | 1.49±0.08 | 1.71±0.05 | 2.11±0.83 | #N/A | 1.68±0.07 | 1.98±0.96 | 1.41±0.07 | 1.67±0.08 |
| 91 | (Z)-β-Ocimene | 15.13 | 3338-55-4 | Hydrocarbons | C10H16 | 1027 | 1038-S | 2.36±0.88 | 1.76±0.19 | 2.23±0.05 | #N/A | #N/A | #N/A | #N/A | #N/A | #N/A |
| 92 | γ-Terpinene | 15.93 | 99-85-4 | Hydrocarbons | C10H16 | 1049 | 1060-S | 1.67±0.04 | 1.58±0.04 | 2.42±0.05 | 1.65±0.1 | #N/A | 2.73±0.01 | #N/A | #N/A | #N/A |
| 93 | Terpinolene | 17.06 | 586-62-9 | Hydrocarbons | C10H16 | 1079 | 1088-S | 1.78±0.37 | #N/A | #N/A | #N/A | #N/A | #N/A | #N/A | #N/A | #N/A |
| 94 | Undecane | 17.39 | 1120-21-4 | Hydrocarbons | C11H24 | 1088 | 1100-A | 1.3±0.08 | 0.93±0.17 | 0.85±0.08 | #N/A | #N/A | #N/A | #N/A | #N/A | #N/A |
| 95 | (3Z,5E)-1,3,5-Undecatriene | 20.46 | 19883-27-3 | Hydrocarbons | C11H18 | 1173 | 1182-S | 0.99±0.06 | #N/A | #N/A | #N/A | #N/A | #N/A | 1.39±0.56 | #N/A | 2.62±0.28 |
| 96 | Naphthalene | 20.53 | 91-20-3 | Hydrocarbons | C10H8 | 1175 | 1182-S | 31.57±2.78 | 52.28±15.27 | 46.85±2.15 | 39.78±7.91 | 35.27±2.44 | 60.4±0.13 | 46.86±1.24 | 60.69±2.12 | 62.28±0.95 |
| 97 | Dodecane | 21.06 | 112-40-3 | Hydrocarbons | C12H26 | 1189 | 1200-A | 3.39±0.28 | 2.02±0.14 | 2.77±0.43 | 2.12±1.36 | 1.71±1.24 | 1.47±0.16 | 1±0.05 | 1.4±0.09 | 1.54±0.69 |
| 98 | 1-Methylnaphthalene | 24.39 | 90-12-0 | Hydrocarbons | C11H10 | 1286 | 1307-S | 9.34±0.97 | 11.08±4 | 14.17±0.53 | 11±3.39 | 9.91±0.43 | 16.04±1.92 | #N/A | 17.15±0.01 | #N/A |
| 99 | Tridecane | 24.53 | 629-50-5 | Hydrocarbons | C13H28 | 1289 | 1300-A | 2.54±0.09 | 3.39±0.41 | 4.73±2.39 | 3.02±2.6 | 3.38±3.61 | 1.82±0.07 | 1.12±0.08 | 1.42±0.15 | 2.16±1.22 |
| 100 | Dehydro-ar-ionene | 26.46 | 30364-38-6 | Hydrocarbons | C13H16 | 1347 | 1354-S | 4.91±0.88 | 7.59±2.81 | 14.7±0.47 | 4.84±1.94 | 5.3±2.04 | 14.69±0.77 | 6.93±0.85 | 10.02±0.97 | 15.55±1.39 |
| 101 | 3-Methyltridecane | 26.86 | 6418-41-3 | Hydrocarbons | C14H30 | 1358 | 1371-S | 1.94±1.15 | #N/A | 3.67±0.09 | 3.27±0.26 | #N/A | #N/A | 1.34±0.27 | 2.42±1.51 | 2.25±1.41 |
| 102 | Biphenyl | 27.26 | 92-52-4 | Hydrocarbons | C12H10 | 1371 | 1381-S | 2.51±0.23 | 5.28±1.78 | 6.08±1.75 | 2.34±0.45 | 5.24±2.26 | 6.49±1.98 | 3.59±0.33 | 4.65±0.13 | 6.96±2.99 |
| 103 | 1,2,4-Metheno-1H-indene, octahydro-1,7a-dimethyl-5-(1-methylethyl)-, [1S-(1α,2α,3aβ,4α,5α,7aβ,8S*)]- | 27.53 | 22469-52-9 | Hydrocarbons | C15H24 | 1378 | 1368-S | 6.9±3.5 | 4.76±1.61 | #N/A | #N/A | #N/A | 16.12±4.9 | #N/A | #N/A | 10.96±0.15 |
| 104 | Tetradecane | 27.79 | 629-59-4 | Hydrocarbons | C14H30 | 1386 | 1400-A | 3.33±0.18 | 7.06±1.98 | 6.27±2.2 | 5.14±2.93 | 4.94±1.86 | 4.36±0.73 | 3.14±0.35 | 4.53±0.31 | 5.97±2.21 |
| 105 | Longifolene | 28.26 | 475-20-7 | Hydrocarbons | C15H24 | 1401 | 1406-S | 1.65±0.18 | 2.19±0.09 | 3.28±0.08 | #N/A | 2.12±0.55 | 3.23±0.29 | 1.08±0.08 | 1.9±0.07 | 2.36±0.07 |
| 106 | 1,7-Dimethyl-naphthalene | 28.53 | 575-37-1 | Hydrocarbons | C12H12 | 1410 | 1419-S | 2.78±0.44 | 8.45±0.01 | 5.26±0.88 | 3.27±1.14 | 3.68±0.84 | 7.56±2.63 | 5.07±1.07 | 8.51±1.09 | 6.98±0.14 |
| 107 | Pentadecane, 3-methyl- | 32.99 | 2882-96-4 | Hydrocarbons | C16H34 | 1557 | 1570-S | 2.64±0.24 | #N/A | #N/A | #N/A | #N/A | #N/A | #N/A | #N/A | #N/A |
| 108 | Fluorene | 33.59 | 86-73-7 | Hydrocarbons | C13H10 | 1577 | 1583-S | 2.38±0.49 | 3.88±1.07 | 4.04±1.18 | 3.88±0.28 | 4.84±1.22 | 4.67±1.96 | 2.85±1.42 | 5.02±0.35 | 5.51±0.15 |
| 109 | Hexadecane | 33.86 | 544-76-3 | Hydrocarbons | C16H34 | 1586 | 1600-A | 4.07±0.27 | 6.13±2.29 | 5.85±1.33 | 6.28±3.14 | 8.24±4.83 | 4.79±0.76 | 7.2±5.29 | 6.49±4.28 | 7.61±6.83 |
| 110 | 1,1'-Biphenyl, 2,2',5,5'-tetramethyl- | 36.26 | 3075-84-1 | Hydrocarbons | C16H18 | 1676 | 1663-S | 3.59±0.22 | 5.47±1.12 | 7.25±1.73 | 3.59±0.53 | 5.57±1.07 | 6.85±1.27 | 3.94±2.32 | 5.22±2.87 | 7.28±0.32 |
| 111 | 1,1'-Biphenyl, 3,4-diethyl- | 36.46 | 61141-66-0 | Hydrocarbons | C16H18 | 1684 | 1692-S | 3.82±2.33 | 3.39±0.62 | 4.5±2.15 | 2.04±0.46 | 2.83±0.57 | 5.76±2.37 | 1540.58±2663.32 | 5.05±3.51 | 5.42±1.42 |
| 112 | Heptadecane | 36.59 | 629-78-7 | Hydrocarbons | C17H36 | 1688 | 1700-A | 2.24±0.82 | 3.02±1.13 | 2.78±0.9 | 2.3±0.35 | 1.81±0.48 | 3.23±1.98 | 1.93±0.45 | 1.35±0.1 | 1.67±0.35 |
| 113 | Mesitylene | 13.47 | 108-67-8 | Hydrocarbons | C9H12 | 982 | 972-S | #N/A | 3.27±0.2 | #N/A | 2.03±0.46 | #N/A | #N/A | 2.62±0.1 | #N/A | #N/A |
| 114 | 1,2,3-Trimethylbenzene | 14.54 | 526-73-8 | Hydrocarbons | C9H12 | 1011 | 1013-S | #N/A | 5.27±0.32 | 4.65±0.28 | #N/A | 1.72±0.08 | 7.16±0.93 | #N/A | #N/A | 5.94±0.36 |
| 115 | Dodecane, 2,6,11-trimethyl- | 23.87 | 31295-56-4 | Hydrocarbons | C15H32 | 1269 | 1275-S | #N/A | 0.92±0.01 | #N/A | #N/A | #N/A | #N/A | #N/A | #N/A | #N/A |
| 116 | 2-Methylnaphthalene | 24.41 | 91-57-6 | Hydrocarbons | C11H10 | 1286 | 1297-S | #N/A | 13.83±3.84 | #N/A | #N/A | #N/A | #N/A | #N/A | #N/A | 17.25±0.44 |
| 117 | Naphthalene, 2,3,6-trimethyl- | 32.54 | 829-26-5 | Hydrocarbons | C13H14 | 1542 | 1550-S | #N/A | 1.69±0.03 | #N/A | #N/A | #N/A | #N/A | #N/A | #N/A | #N/A |
| 118 | Naphthalene, 1,6-dimethyl-4-(1-methylethyl)- | 36.21 | 483-78-3 | Hydrocarbons | C15H18 | 1674 | 1674-S | #N/A | 1.72±0 | 2.06±0.14 | 1.74±0.4 | 3.19±1.08 | 3.07±0.75 | 3.3±1.09 | 2.19±0.18 | 3.66±0.98 |
| 119 | p-Xylene | 9.08 | 106-42-3 | Hydrocarbons | C8H10 | 856 | 865-S | #N/A | #N/A | 1.78±0.77 | 5.33±2.11 | #N/A | #N/A | 7.1±1.05 | 7.98±0.41 | 5.04±0.08 |
| 120 | 1-Dodecene | 21.55 | 112-41-4 | Hydrocarbons | C12H24 | 1202 | 1190-S | #N/A | #N/A | 52.05±1.29 | 5.59±0 | 44.71±0.03 | #N/A | #N/A | 31.03±2.22 | #N/A |
| 121 | 1H-3a,7-Methanoazulene, 2,3,4,7,8,8a-hexahydro-3,6,8,8-tetramethyl-, [3R-(3α,3aβ,7β,8aα)]- | 28.48 | 469-61-4 | Hydrocarbons | C15H24 | 1407 | 1411-S | #N/A | #N/A | 1.7±0.05 | #N/A | #N/A | 1.58±0.06 | 0.97±0.06 | 1.25±0.06 | 1.5±0.03 |
| 122 | Naphthalene, 1,2,4a,5,8,8a-hexahydro-4,7-dimethyl-1-(1-methylethyl)-, [1S-(1α,4aβ,8aα)]- | 31.82 | 523-47-7 | Hydrocarbons | C15H24 | 1517 | 1518-S | #N/A | #N/A | 1.29±0.01 | #N/A | #N/A | #N/A | #N/A | #N/A | #N/A |
| 123 | o-Cymene | 14.68 | 527-84-4 | Hydrocarbons | C10H14 | 1015 | 1022-S | #N/A | #N/A | #N/A | #N/A | 1.07±0.02 | #N/A | #N/A | #N/A | #N/A |
| 124 | 3-Carene | 15.55 | 13466-78-9 | Hydrocarbons | C10H16 | 1038 | 1011-S | #N/A | #N/A | #N/A | #N/A | 1.37±0.12 | 8.98±6.97 | #N/A | #N/A | 2.11±0.08 |
| 125 | Toluene | 6.06 | 108-88-3 | Hydrocarbons | C7H8 | 741 | 763-S | #N/A | #N/A | #N/A | #N/A | #N/A | 1.36±0.02 | #N/A | #N/A | #N/A |
| 126 | Ethylbenzene | 8.79 | 100-41-4 | Hydrocarbons | C8H10 | 847 | 850-N | #N/A | #N/A | #N/A | #N/A | #N/A | 1.49±0.03 | 0.98±0.02 | 6.7±1.45 | #N/A |
| 127 | o-Xylene | 9.06 | 95-47-6 | Hydrocarbons | C8H10 | 856 | 888-S | #N/A | #N/A | #N/A | #N/A | #N/A | 7.57±1.31 | #N/A | #N/A | #N/A |
| 128 | Benzene, 1-ethyl-4-(1-methylethyl)- | 17.13 | 4218-48-8 | Hydrocarbons | C11H16 | 1081 | 1096-S | #N/A | #N/A | #N/A | #N/A | #N/A | 3.14±0.04 | #N/A | #N/A | 4.14±0.26 |
| 129 | β-Bisabolene | 31.33 | 495-61-4 | Hydrocarbons | C15H24 | 1500 | 1509-S | #N/A | #N/A | #N/A | #N/A | #N/A | 2.66±0.62 | #N/A | #N/A | #N/A |
| 130 | 1,2,4-Trimethylbenzene | 14.59 | 95-63-6 | Hydrocarbons | C9H12 | 1013 | 990-S | #N/A | #N/A | #N/A | #N/A | #N/A | #N/A | 4.53±0.57 | 6.43±0.01 | #N/A |
| 131 | Safrole | 24.26 | 94-59-7 | Hydrocarbons | C10H10O2 | 1282 | 1287-S | #N/A | #N/A | #N/A | #N/A | #N/A | #N/A | 1.23±0.07 | #N/A | #N/A |
| 132 | Naphthalene, 1,4-dimethyl- | 28.53 | 571-58-4 | Hydrocarbons | C12H12 | 1410 | 1436-S | #N/A | #N/A | #N/A | #N/A | #N/A | #N/A | 5.93±0.42 | #N/A | #N/A |
| 133 | Cyclohexene, 5-methyl-3-(1-methylethenyl)-, trans-(-)- | 21.33 | 56816-08-1 | Hydrocarbons | C10H16 | 1197 | 1192-S | #N/A | #N/A | #N/A | #N/A | #N/A | #N/A | #N/A | 2.28±0.79 | #N/A |
| 134 | Dodecane, 5,8-diethyl- | 32.99 | 24251-86-3 | Hydrocarbons | C16H34 | 1557 | 1572-S | #N/A | #N/A | #N/A | #N/A | #N/A | #N/A | #N/A | 1.66±0.13 | #N/A |
| 135 | Heptadecane, 2,6,10,15-tetramethyl- | 36.59 | 54833-48-6 | Hydrocarbons | C21H44 | 1688 | 1889-S | #N/A | #N/A | #N/A | #N/A | #N/A | #N/A | #N/A | 1.34±0.06 | #N/A |
| 136 | Hexanoic acid | 12.73 | 142-62-1 | Acids | C6H12O2 | 962 | 990-S | 1.58±0.34 | 4.13±0.08 | 2.38±0 | #N/A | 2.47±0.86 | 4.7±0.11 | #N/A | 6.87±1.82 | 5.73±0.96 |
| 137 | Nonanoic acid | 23.33 | 112-05-0 | Acids | C9H18O2 | 1254 | 1273-S | 2.1±0.79 | 2.19±0.35 | 2.09±0.65 | #N/A | 2.53±1.13 | 3.93±0.21 | 2.34±0.54 | 2.84±0.73 | 4.05±0.55 |
| 138 | Naphthalene, 2-methoxy- | 29.59 | 34068 | Acids | C11H10O | 1443 | 1458-S | 15.81±2.11 | 21±1.92 | 20.21±0.2 | 16.56±4.3 | 18.71±0.23 | 26.01±4.55 | 19.47±1.21 | 26.54±1.11 | 31.88±3.62 |
| 139 | Hexanal | 6.86 | 66-25-1 | Aldehydes | C6H12O | 779 | 801-S | 39.66±2.17 | 30.64±10.42 | 17.44±0.92 | 38.49±7.72 | 19.74±3.95 | 18.61±3.53 | 39.72±2.11 | 31.07±3.27 | 21.22±0.51 |
| 140 | (E)-2-Hexenal | 8.53 | 6728-26-3 | Aldehydes | C6H10O | 839 | 854-S | 7.58±0.69 | 3.13±1.29 | 1.81±0.05 | 5.52±0.67 | 3.36±0.8 | 1.52±0.02 | 5.16±0.28 | 3.21±1.18 | 1.72±0.06 |
| 141 | (Z)-4-Heptenal | 10.06 | 6728-31-0 | Aldehydes | C7H12O | 888 | 900-S | 8.68±0.44 | 8.31±0.48 | #N/A | 8.49±1.35 | 6.49±0.65 | #N/A | 8.98±0.88 | 7.59±0.69 | 5.91±0.26 |
| 142 | Heptanal | 10.13 | 111-71-7 | Aldehydes | C7H14O | 890 | 901-S | 18.06±1.46 | 13.77±6.53 | 10.52±4.14 | 15.18±4.41 | 9.45±3.59 | 8.54±1.87 | 17.46±1.24 | 17.57±3.93 | 11.65±0.73 |
| 143 | benzaldehyde | 12.26 | 100-52-7 | Aldehydes | C7H6O | 950 | 962-S | 33.74±2.96 | 64.58±7.15 | 63.52±2.9 | 44.33±6.73 | 35.92±2.94 | 91.58±5.89 | 42.66±0.63 | 75.24±2.23 | 87.93±3.38 |
| 144 | octanal | 13.79 | 124-13-0 | Aldehydes | C8H16O | 991 | 1003-S | 19.2±1.53 | 22.21±7.16 | 19.97±1.1 | 19.22±5.15 | 20.37±4.26 | #N/A | 18.57±3.41 | 23.89±8.66 | #N/A |
| 145 | (E,E)-2,4-Heptadienal | 14.13 | 881395 | Aldehydes | C7H10O | 1001 | 1012-S | 14.53±0.53 | 24.46±9.45 | 10.15±0.62 | 16.13±4.75 | 30.36±4.35 | 14.72±3.1 | 11.58±8.04 | 31.93±24.49 | 23.43±0.91 |
| 146 | benzeneacetaldehyde | 15.39 | 122-78-1 | Aldehydes | C8H8O | 1035 | 1045-S | 30.64±3.5 | 29.48±1.49 | 35.82±2.14 | 26.55±3.57 | 28.97±3.49 | 37.43±1.96 | 24.62±1.64 | 31.54±2.35 | 38.5±5.64 |
| 147 | (E)-2-Octenal | 15.86 | 2548-87-0 | Aldehydes | C8H14O | 1047 | 1060-S | 15.26±1.32 | 9.17±3.61 | 3.42±0.19 | 13.75±3.98 | 9.89±1.06 | 3.48±0.37 | 15.22±0.82 | 12.89±1.55 | 6.1±0.55 |
| 148 | nonanal | 17.59 | 124-19-6 | Aldehydes | C9H18O | 1093 | 1104-S | 109.08±9.85 | 101.83±5.21 | 96.62±14.63 | 130.66±4.68 | 110.39±18.93 | 88.76±7.85 | 101.69±3.87 | 106.71±5.39 | 105.13±4.6 |
| 149 | (E,Z)-2,6-Nonadienal | 19.39 | 557-48-2 | Aldehydes | C9H14O | 1143 | 1155-S | 17.93±1.16 | #N/A | #N/A | 19.48±5.44 | 17.32±7.06 | #N/A | 15.22±1.63 | #N/A | #N/A |
| 150 | (E)-2-Nonenal | 19.59 | 18829-56-6 | Aldehydes | C9H16O | 1149 | 1162-S | 25.58±2.48 | 15±3.73 | 7.18±0.57 | 25.63±5.52 | 15.89±1.16 | 9.08±1.62 | 28.68±1.28 | 19.39±1.07 | 12.73±1.88 |
| 151 | 2,4-Dimethylbenzaldehyde | 20.19 | 15764-16-6 | Aldehydes | C9H10O | 1166 | 1182-S | 9.04±1.83 | 13.34±5.36 | 16.21±0.62 | 10.78±3.33 | 10.95±0.63 | #N/A | 9.72±2.24 | 18.92±0.25 | 22.29±0.45 |
| 152 | Benzaldehyde, 3-methoxy- | 20.93 | 591-31-1 | Aldehydes | C8H8O2 | 1187 | 1196-S | 6.66±0.5 | 6.97±0.63 | #N/A | #N/A | #N/A | 7.29±0.93 | #N/A | 8.65±1.4 | #N/A |
| 153 | Safranal | 21.13 | 116-26-7 | Aldehydes | C10H14O | 1192 | 1201-S | 44.66±4.1 | 118.31±16.29 | 113.64±4.36 | 76.97±20.4 | 65.71±9.26 | 154.18±5.99 | 95.8±2.23 | 141.9±4.74 | 154.27±0.85 |
| 154 | Decanal | 21.26 | 112-31-2 | Aldehydes | C10H20O | 1195 | 1206-S | 69.56±7.82 | 67.76±3.51 | 35.15±7.75 | 68.43±3.75 | 53.87±9.01 | 29.1±2.32 | 60.92±1.06 | 45.96±4.91 | 38.59±2.7 |
| 155 | (E,E)-2,4-Nonadienal | 21.59 | 5910-87-2 | Aldehydes | C9H14O | 1205 | 1216-S | 5.02±1.24 | #N/A | #N/A | #N/A | #N/A | #N/A | 6.56±0.99 | #N/A | #N/A |
| 156 | β-cyclocitral | 21.93 | 432-25-7 | Aldehydes | C10H16O | 1214 | 1220-S | 24.38±2.2 | 55.7±9.81 | 51.33±2.37 | 39.36±13.87 | 29.66±5.39 | 74.11±3.08 | 40.28±11.46 | 71.08±4.1 | 82.25±3.69 |
| 157 | Benzaldehyde, 2-methoxy- | 22.66 | 135-02-4 | Aldehydes | C8H8O2 | 1234 | 1222-S | 4.53±2.29 | 5.51±0.31 | 4.87±1.12 | 3.99±0.77 | 4.96±0.9 | 4.95±1.33 | 4.49±0.88 | 6.46±1 | 6.34±2.46 |
| 158 | 1-Cyclohexene-1-acetaldehyde, 2,6,6-trimethyl- | 23.19 | 472-66-2 | Aldehydes | C11H18O | 1251 | 1254-S | 1.69±0.09 | 3.71±0.04 | 5.21±0.24 | 3.14±1.12 | 2.72±1.12 | 6.6±0.2 | 2.72±0.38 | 4.65±0.36 | 6.38±0.63 |
| 159 | Undecanal | 24.79 | 112-44-7 | Aldehydes | C11H22O | 1297 | 1307-S | 4.93±0.2 | 5.18±0.2 | 3.66±0.44 | 4.8±0.15 | 4.38±0.81 | 3.1±0.26 | 4.2±0.41 | 3.66±0.31 | 3.62±0.55 |
| 160 | Dodecanal | 28.13 | 112-54-9 | Aldehydes | C12H24O | 1396 | 1409-S | 8.36±0.32 | 10.58±2.34 | 13.79±0.68 | 5.59±0.3 | 14±0.64 | 12.28±1.18 | 5.22±0.85 | 8.12±0.43 | 12.64±0.38 |
| 161 | Salicylaldehyde | 15.34 | 32912 | Aldehydes | C7H6O2 | 1033 | 1047-S | #N/A | 3.51±0.3 | #N/A | #N/A | #N/A | #N/A | 2.43±0.09 | 3.72±0.37 | #N/A |
| 162 | 2-Cyclohexene-1-carboxaldehyde, 2,6,6-trimethyl- | 18.14 | 432-24-6 | Aldehydes | C10H16O | 1108 | 1116-S | #N/A | 3.52±1.31 | 4.62±0.32 | 2.46±0.59 | #N/A | 6.75±0.56 | 2.91±0.23 | 5.19±0.22 | 6.86±0.33 |
| 163 | Benzaldehyde, 2-amino- | 21.74 | 529-23-7 | Aldehydes | C7H7NO | 1208 | 1222-S | #N/A | 2.29±0.04 | 3.94±0.82 | #N/A | 2.09±0.13 | 4.66±1.65 | #N/A | 2.68±0.33 | #N/A |
| 164 | (E,E)-2,4-Decadienal | 25.14 | 25152-84-5 | Aldehydes | C10H16O | 1307 | 1317-S | #N/A | 13.62±4.07 | 15.28±2.52 | #N/A | 17.41±4.24 | 19.07±2.72 | #N/A | 24.49±2.01 | 26.53±0.03 |
| 165 | Benzaldehyde, 2,4,6-trimethyl- | 25.22 | 487-68-3 | Aldehydes | C10H12O | 1309 | 1339-S | #N/A | #N/A | 10.39±0.38 | #N/A | #N/A | 15.79±1.01 | #N/A | 16.1±0.43 | 19.11±2.46 |
| 166 | 3-Cyclohexene-1-carboxaldehyde, 4-methyl- | 14.93 | 7560-64-7 | Aldehydes | C8H12O | 1022 | 1024-S | #N/A | #N/A | #N/A | #N/A | #N/A | #N/A | 1.02±0.01 | 1.75±0.3 | #N/A |
| 167 | 4-(t-Butyl)benzaldehyde | 26.46 | 939-97-9 | Aldehydes | C11H14O | 1347 | 1329-S | #N/A | #N/A | #N/A | #N/A | #N/A | #N/A | #N/A | 1.89±0.38 | 1.93±0.21 |
| 168 | (E，Z)-2,4-Decadienal | 25.13 | 25152-83-4 | Aldehydes | C10H16O | 1307 | 1295-S | #N/A | #N/A | #N/A | #N/A | #N/A | #N/A | #N/A | #N/A | 16.14±11.74 |
| 169 | Tetrachloroethylene | 7.19 | 127-18-4 | Others | C2Cl4 | 794 | 815-S | 34.47±4.15 | 87.92±46.45 | 142.11±8.66 | 11.6±6.26 | 33.44±17.8 | 98.64±20.06 | 4.05±0.4 | 99.83±1.94 | 98.14±5.36 |
| 170 | Dimethyl trisulfide | 12.61 | 3658-80-8 | Others | C2H6S3 | 959 | 971-S | #N/A | 2.19±0.23 | 6.66±1.62 | #N/A | 1.43±0.21 | 5.6±0.65 | 2.03±0.32 | 2.52±0.52 | 4.35±0.68 |
| 171 | Indole | 24.47 | 120-72-9 | Others | C8H7N | 1286 | 1295-S | #N/A | 4.58±0.25 | 9.11±0.34 | #N/A | 4.01±1.33 | 10.58±0.54 | #N/A | 4.66±0.34 | 11.22±0.75 |
| 172 | .tau.-Muurolol | 35.62 | 19912-62-0 | Others | C15H26O | 1651 | 1642-S | #N/A | #N/A | 1.1±0.14 | #N/A | #N/A | 4.11±0.38 | 5.62±0.92 | #N/A | 5.81±1.05 |
| 173 | Dimethyl disulfide | 5.53 | 624-92-0 | Others | C2H6S2 | 716 | 730-N | #N/A | #N/A | #N/A | #N/A | #N/A | 1.7±0.12 | #N/A | #N/A | #N/A |
| 174 | Formamide, N-phenyl- | 21.74 | 103-70-8 | Others | C7H7NO | 1208 | 1221-S | #N/A | #N/A | #N/A | #N/A | #N/A | #N/A | #N/A | #N/A | 4.15±0.05 |
| 175 | Phenol | 12.93 | 108-95-2 | Phenols | C6H6O | 968 | 981-S | 1.78±0.45 | 3.9±1.18 | 2.58±1.64 | 2.16±0.5 | 1.95±1.02 | 3.72±0.14 | 1.79±0.82 | 4.19±0.63 | 5.3±0.1 |
| 176 | Phenol, 2-methoxy- | 17.06 | 32994 | Phenols | C7H8O2 | 1080 | 1090-S | 1.5±0.1 | 3.29±2.2 | 1.77±0.23 | #N/A | 2.14±0.76 | 2.71±0.32 | 1.41±0.01 | 3.28±0.98 | 4.43±1.46 |
| 177 | Phenol, 2,3-dimethyl- | 19.86 | 526-75-0 | Phenols | C8H10O | 1157 | 1180-S | 2.05±0.45 | #N/A | #N/A | 2.55±1.06 | #N/A | #N/A | #N/A | 4.62±0.35 | #N/A |
| 178 | Benzene, 1,2-dimethoxy-4-propenyl-, (Z)- | 29.66 | 6380-24-1 | Phenols | C11H14O2 | 1447 | 1456-S | 3.8±0.08 | 5.04±0.44 | 3.53±0.19 | 3.07±0.66 | 3.61±0.31 | #N/A | 5.86±1.18 | 5.46±1.45 | 4.35±0.21 |
| 179 | 2,4-Di-t-butylphenol | 31.33 | 96-76-4 | Phenols | C14H22O | 1501 | 1514-S | 49.16±5.26 | 59.19±17.7 | 76±7.47 | 39.84±17.35 | 39.22±4.73 | 58.31±3.05 | 56.86±5.94 | 62.13±6.35 | 64.85±2.3 |
| 180 | Butylated hydroxytoluene | 31.39 | 128-37-0 | Phenols | C15H24O | 1503 | 1513-S | 27.54±6.59 | #N/A | #N/A | 8.25±2.72 | #N/A | #N/A | #N/A | #N/A | #N/A |
| 181 | Cedrol | 34.19 | 77-53-2 | Phenols | C15H26O | 1599 | 1600-S | 6.73±0.12 | 10.11±1 | 6.01±2.05 | 7.23±1.12 | 7.56±0.95 | 8.47±1.27 | 12.32±9.38 | 8.87±0.96 | 11.12±1.32 |
| 182 | Carvacrol | 24.61 | 499-75-2 | Phenols | C10H14O | 1291 | 1299-S | #N/A | 3.27±1.4 | #N/A | #N/A | #N/A | 3.69±0.27 | #N/A | 11.3±5.07 | 5.22±0.37 |
| 183 | Thymol | 24.62 | 89-83-8 | Phenols | C10H14O | 1291 | 1291-S | #N/A | #N/A | 3±1.35 | #N/A | #N/A | 6.4±0.68 | #N/A | 4.33±1.75 | 9.49±3.54 |
| 184 | Phenol, 3,5-dimethyl- | 19.88 | 108-68-9 | Phenols | C8H10O | 1157 | 1171-S | #N/A | #N/A | #N/A | #N/A | 2.91±0.14 | #N/A | #N/A | #N/A | 4.96±0.37 |
| 185 | 2-Ethylphenol | 19.86 | 90-00-6 | Phenols | C8H10O | 1157 | 1140-S | #N/A | #N/A | #N/A | #N/A | #N/A | 3.11±1.26 | #N/A | #N/A | #N/A |
| 186 | 3-Methylphenol | 15.66 | 108-39-4 | Phenols | C7H8O | 1042 | 1075-S | #N/A | #N/A | #N/A | #N/A | #N/A | #N/A | #N/A | 1.68±0.16 | 1.81±0.17 |
| 187 | 1-octen-3-ol | 12.93 | 3391-86-4 | Alcohols | C8H16O | 968 | 980-S | 3.82±0.32 | 10.9±2 | 4.75±0.06 | 10.76±3.1 | 3.26±0.27 | 13.71±1.77 | 11.33±0.77 | 15.14±0.64 | 13.17±1.69 |
| 188 | 2-Ethyl-1-hexanol | 14.73 | 104-76-7 | Alcohols | C8H18O | 1016 | 1030-S | 25.45±13.79 | 103.91±18.32 | 115±5.01 | 61.38±6.71 | 81.27±4.9 | 150.82±8.68 | 49.32±13.2 | 137.85±7.56 | 161.02±5.17 |
| 189 | 1-Octanol | 16.33 | 111-87-5 | Alcohols | C8H18O | 1059 | 1070-S | 2.57±0.27 | 7.1±0.92 | 4.66±0.38 | 5.6±1.16 | 3.53±0.4 | 8.74±0.23 | 6.42±0.39 | 11±0.46 | 9.42±0.33 |
| 190 | Linalool oxide I | 16.46 | 5989-33-3 | Alcohols | C10H18O2 | 1063 | 1074-S | 55.73±29.06 | #N/A | 106.38±4.33 | 223.06±119.74 | 58.73±2.61 | #N/A | #N/A | 202.26±2.73 | 188.43±13.48 |
| 191 | Linalool oxide II | 17.06 | 34995-77-2 | Alcohols | C10H18O2 | 1079 | 1086-S | 59.33±5.84 | 310.23±82.18 | 149.53±5.75 | 177.38±32.32 | 87.53±6.11 | 381.76±111.99 | 232.84±71.52 | 368.08±147.53 | 322.05±100.39 |
| 192 | linalool | 17.46 | 78-70-6 | Alcohols | C10H18O | 1090 | 1099-S | 99.1±10.32 | 153.42±23.9 | 202.33±10.13 | 144.03±32.54 | 80.77±7.87 | 273.83±8.05 | 158.06±5.97 | 190.66±6.62 | 239.28±4.54 |
| 193 | Benzeneethanol | 17.93 | 22258 | Alcohols | C8H10O | 1103 | 1116-S | 12.04±1 | 16.91±0.44 | 14.21±0.43 | 13.29±2.36 | 12.32±1.65 | 21.43±1.69 | 11.45±0.46 | 20.2±2.47 | 20.86±1.39 |
| 194 | Linalool oxide III | 19.99 | 39028-58-5 | Alcohols | C10H18O2 | 1160 | 1173-S | 94.56±5.63 | 166.6±12.67 | 126.26±1.37 | 126.49±32.01 | 98.38±6.71 | 201.15±10.83 | 89.19±50.99 | 204.83±18.56 | 208.29±2.12 |
| 195 | 4-Terpineol | 20.33 | 562-74-3 | Alcohols | C10H18O | 1169 | 1177-S | 3.95±0.09 | 14.21±0.2 | 8.5±0.39 | 9.82±3.68 | 5.66±0.42 | 15.07±0.64 | 11.09±0.92 | #N/A | 13.25±0.5 |
| 196 | α-Terpineol | 20.79 | 98-55-5 | Alcohols | C10H18O | 1182 | 1189-S | 62.89±5.24 | 140.15±11.09 | 123.31±2.84 | 114.17±22.67 | 88.43±5.93 | 177.57±14.69 | 127.45±3.5 | 179.15±8.11 | 180.38±6.21 |
| 197 | Nerol | 22.06 | 106-25-2 | Alcohols | C10H18O | 1218 | 1228-S | 9.86±3.16 | 14.23±0.6 | 13.1±0.39 | 15.71±5.6 | 11.62±3.8 | 14.95±7.95 | 10.49±6.74 | 19±2.45 | 26.59±3.11 |
| 198 | 1-Cyclohexene-1-ethanol, 2,6,6-trimethyl- | 25.73 | 472-65-1 | Alcohols | C11H20O | 1325 | 1337-S | 2.78±0.29 | 5.43±0.68 | 6.93±0.65 | 5.56±2.69 | 5.28±1.06 | 9.4±2.59 | 7.83±1 | 11.64±1.31 | 11.75±0.63 |
| 199 | 1-Dodecanol | 30.13 | 112-53-8 | Alcohols | C12H26O | 1461 | 1474-S | 156.29±7.86 | 222.17±27.15 | 356.53±22.74 | 59±19.29 | 421.17±19.45 | 351.7±35.45 | 28.09±5.19 | 244.8±7.64 | 386.06±5.56 |
| 200 | Nerolidol | 32.86 | 142-50-7 | Alcohols | C15H26O | 1553 | 1544-S | 1.21±0.02 | #N/A | #N/A | #N/A | #N/A | #N/A | #N/A | #N/A | #N/A |
| 201 | .tau.-Cadinol | 35.66 | 1474790 | Alcohols | C15H26O | 1654 | 1640-S | 1.69±0.35 | 3.28±1.18 | 3.37±1.32 | 3.99±2.12 | 3.7±3.29 | 3.6±2.44 | 2.33±0.06 | 4.35±2.16 | 4.77±3.36 |
| 202 | benzyl alcohol | 14.94 | 100-51-6 | Alcohols | C7H8O | 1023 | 1036-S | #N/A | 4.66±2.6 | 1.41±0.52 | 2.33±0.16 | 1.92±0.68 | 3.33±0.99 | #N/A | 2.22±0.13 | 3.07±0.44 |
| 203 | 3,5-Octadien-2-ol | 15.21 | 69668-82-2 | Alcohols | C8H14O | 1029 | 1038-S | #N/A | 2.53±0.44 | #N/A | #N/A | #N/A | 5.65±1.37 | #N/A | 4.51±0.03 | 3.77±1.2 |
| 204 | 2-Cyclohexen-1-ol, 2,4,4-trimethyl- | 15.67 | 73741-61-4 | Alcohols | C9H16O | 1042 | 1054-S | #N/A | 7.42±2.03 | 3.66±1.12 | 3.37±1.28 | 2.6±0.45 | 9.41±2.41 | 5.41±0.84 | 10.82±0.59 | 8.98±1.31 |
| 205 | 5-Isopropyl-2-methylbicyclo[3.1.0]hexan-2-ol # | 17.47 | 546-79-2 | Alcohols | C10H18O | 1090 | 1075-S | #N/A | 6.29±0.01 | #N/A | #N/A | 3.23±1.3 | #N/A | #N/A | #N/A | #N/A |
| 206 | Levomenthol | 20.14 | 2216-51-5 | Alcohols | C10H20O | 1164 | 1175-S | #N/A | 2.22±0.18 | 1.75±0.14 | 2.34±0.18 | 1.81±0.94 | 2.25±0.15 | #N/A | 4.07±0.54 | 3.93±0.07 |
| 207 | α,α-4-Trimethylbenzenemethanol | 20.61 | 1197-01-9 | Alcohols | C10H14O | 1177 | 1183-S | #N/A | 2.39±0.19 | 1.61±0.05 | 2.24±1.68 | 1.57±0.34 | 3.02±0.15 | 1.73±0.11 | 2.51±0.12 | 2.97±0.32 |
| 208 | Lilac alcohol A | 21.54 | 33081-34-4 | Alcohols | C10H18O2 | 1203 | 1211-S | #N/A | 10.61±4.79 | 7.4±0.17 | 7.88±3.07 | 10.81±2.96 | 10.67±0.36 | #N/A | 13.08±1.66 | 13.16±6.66 |
| 209 | (E)-Nerolidol | 32.87 | 40716-66-3 | Alcohols | C15H26O | 1553 | 1564-S | #N/A | 2.23±0.05 | 4.03±0.33 | 1.96±0.64 | 2.28±0.72 | 3.6±0.95 | 2.14±0.28 | #N/A | 5.01±0.23 |
| 210 | 3,4-Dimethylcyclohexanol | 17.81 | 5715-23-1 | Alcohols | C8H16O | 1099 | 1126-S | #N/A | #N/A | #N/A | 12.16±3.85 | 8.53±0.67 | #N/A | 12.41±0.47 | #N/A | #N/A |
| 211 | (R)-3,5,5-Trimethylcyclohex-3-en-1-ol | 16.06 | 211107-48-1 | Alcohols | C9H16O | 1052 | 1067-S | #N/A | #N/A | #N/A | #N/A | #N/A | 2.26±0.56 | 0.99±0 | 2.6±0.32 | 2.57±0.03 |
| 212 | 1-Heptanol | 12.59 | 111-70-6 | Alcohols | C7H16O | 959 | 970-S | #N/A | #N/A | #N/A | #N/A | #N/A | #N/A | 0.97±0.02 | 1.5±0.05 | #N/A |
| 213 | Eucalyptol | 14.93 | 470-82-6 | Alcohols | C10H18O | 1022 | 1032-S | #N/A | #N/A | #N/A | #N/A | #N/A | #N/A | 1.64±0.51 | 1.84±0.18 | #N/A |
| 214 | dl-Menthol | 20.13 | 89-78-1 | Alcohols | C10H20O | 1164 | 1173-S | #N/A | #N/A | #N/A | #N/A | #N/A | #N/A | 2.7±0.45 | #N/A | #N/A |
| 215 | 3-Cyclohexen-1-ol, 4-methyl-1-(1-methylethyl)-, (R)- | 20.33 | 20126-76-5 | Alcohols | C10H18O | 1169 | 1182-S | #N/A | #N/A | #N/A | #N/A | #N/A | #N/A | #N/A | 17.47±0.67 | #N/A |
| 216 | Furan, 2,3,5-trimethyl- | 7.33 | 10504-04-8 | Pyrroles and their derivatives | C7H10O | 800 | 816-S | 5.55±0.42 | 2.74±1.06 | 2.8±0.09 | 3.78±1.28 | 2.39±0.63 | 2.65±0.52 | 4.71±0.24 | 2.99±0.09 | 2.62±0.09 |
| 217 | 1-Ethyl-1H-pyrrole | 7.33 | 617-92-5 | Pyrroles and their derivatives | C6H9N | 801 | 821-S | 2.29±0.27 | 1.89±0.18 | 4.82±0.7 | 1.46±0.2 | 2.04±0.48 | 4.12±0.8 | 2.51±0.76 | 2.72±0.99 | 3.15±0.91 |
| 218 | 2-Amylfuran | 13.39 | 3777-69-3 | Pyrroles and their derivatives | C9H14O | 980 | 993-S | 28.21±2.63 | 27.46±12.46 | 31.82±1.7 | 24.09±8.16 | 26.58±10 | 33.49±2.52 | 29.29±1.97 | 37.65±1.32 | 43.14±1.65 |
| 219 | tea pyrrole | 15.59 | 2167-14-8 | Pyrroles and their derivatives | C7H9NO | 1040 | 1046-S | 23.75±0.55 | #N/A | #N/A | 51.74±13.08 | 34.33±4.53 | #N/A | 50.05±1.08 | 97.04±0.68 | 99.3±0.82 |
| 220 | Benzothiazole | 22.06 | 95-16-9 | Pyrroles and their derivatives | C7H5NS | 1217 | 1228-S | 1.94±0.16 | 3.15±0.38 | 2.87±0.16 | 3.41±2.15 | 4.03±2.6 | 3.72±0.23 | 2.12±0.1 | 3.5±0.24 | 3.96±0.08 |
| 221 | Benzofuran, 7-methoxy- | 23.39 | 7168-85-6 | Pyrroles and their derivatives | C9H8O2 | 1255 | 1259-S | 2.97±0.38 | 10.96±5.69 | 11.89±0.46 | 7.74±5.81 | 12.14±3.43 | 12.93±4.76 | 11.13±0.03 | 13±4.34 | 15.95±5.77 |
| 222 | 2,5-Cyclohexadiene-1,4-dione, 2,6-bis(1,1-dimethylethyl)- | 30.06 | 719-22-2 | Pyrroles and their derivatives | C14H20O2 | 1459 | 1472-S | 5.95±1.15 | 16.21±3.65 | 30.16±2.29 | #N/A | #N/A | 40.87±4.29 | 7.92±1.74 | 20.43±1.8 | 49.59±0.48 |
| 223 | Dibenzofuran | 31.59 | 132-64-9 | Pyrroles and their derivatives | C12H8O | 1509 | 1515-S | 5.68±0.54 | 7.96±0.61 | 8.09±0.5 | 5.5±0.88 | 7.49±2.03 | 9.24±1.33 | 6.46±0.3 | 8.42±0.37 | 9.65±0.45 |
| 224 | 2-Ethylpyridine | 10.34 | 100-71-0 | Pyrroles and their derivatives | C7H9N | 897 | 906-S | #N/A | 2.65±0.34 | 2.64±0.42 | 1.56±0.37 | 1.49±0.08 | 5.86±0.62 | 1.29±0.22 | 3.9±0.59 | 5.9±0.23 |
| 225 | (2R,5S)-2-Methyl-5-(prop-1-en-2-yl)-2-vinyltetrahydrofuran | 14.01 | 54750-69-5 | Pyrroles and their derivatives | C10H16O | 997 | 1008-S | #N/A | 2.34±0.04 | 1.98±0.11 | 2.06±0.16 | #N/A | 2.77±0.29 | 2.12±0.04 | 2.61±0.01 | 2.76±0.17 |
| 226 | 2,5-Furandicarboxaldehyde | 16.87 | 823-82-5 | Pyrroles and their derivatives | C6H4O3 | 1074 | 1076-S | #N/A | 2.27±0.44 | 2.8±0.13 | #N/A | #N/A | 5.71±1.05 | #N/A | 2.57±0.24 | 4±0.33 |
| 227 | 3-Acetyl-2,5-dimethyl furan | 18.41 | 10599-70-9 | Pyrroles and their derivatives | C8H10O2 | 1116 | 1099-S | #N/A | 2.02±0.12 | 1.67±0.13 | #N/A | #N/A | 2.88±0.32 | #N/A | 2.31±0.21 | 2.42±0.06 |
| 228 | Pyridine, 2-pentyl- | 21.01 | 2294-76-0 | Pyrroles and their derivatives | C10H15N | 1188 | 1202-S | #N/A | 4.03±0.62 | 4.44±0.4 | #N/A | 3.19±0.67 | 6.33±0.83 | #N/A | 6.85±0.64 | 7.29±0.64 |
| 229 | Benzofuran, 5-methoxy-6,7-dimethyl- | 30.14 | 35355-35-2 | Pyrroles and their derivatives | C11H12O2 | 1462 | 1468-S | #N/A | 6.29±4.18 | #N/A | #N/A | #N/A | #N/A | 10.5±0.1 | #N/A | 13.83±3.73 |
| 230 | 2,3-Dihydrobenzofuran | 21.68 | 496-16-2 | Pyrroles and their derivatives | C8H8O | 1207 | 1224-S | #N/A | #N/A | 4.69±1.38 | #N/A | 2.51±0.77 | 5.24±1.3 | #N/A | 3.39±0.04 | 4.7±0.08 |
| 231 | Edulan II | 23.22 | 41678-30-2 | Pyrroles and their derivatives | C13H20O | 1251 | 1247-S | #N/A | #N/A | 1.41±0.04 | #N/A | #N/A | #N/A | #N/A | #N/A | #N/A |
| 232 | 2-n-Butyl furan | 9.79 | 4466-24-4 | Pyrroles and their derivatives | C8H12O | 879 | 893-S | #N/A | #N/A | #N/A | #N/A | #N/A | #N/A | 0.82±0.01 | #N/A | #N/A |
| 233 | 2,3,5,6-tetramethylpyrazine | 16.93 | 1124-11-4 | Pyrroles and their derivatives | C8H12N2 | 1076 | 1088-S | #N/A | #N/A | #N/A | #N/A | #N/A | #N/A | #N/A | 4.5±0.44 | 3.39±1.6 |
| 234 | trans-2-(2-Pentenyl)furan | 13.73 | 70424-14-5 | Pyrroles and their derivatives | C9H12O | 990 | 1002-S | #N/A | #N/A | #N/A | #N/A | #N/A | #N/A | #N/A | #N/A | 28.94±0.59 |
| 235 | 2H-Pyran, 3,6-dihydro-4-methyl-2-(2-methyl-1-propenyl)- | 19.47 | 1786-08-9 | Pyrroles and their derivatives | C10H16O | 1145 | 1154-S | #N/A | #N/A | #N/A | #N/A | #N/A | #N/A | #N/A | #N/A | 1.48±0.03 |

a CAS: the published chemical abstracts service (CAS) of compounds in NIST 17 library. b: The data of concentrations is “mean ± standard” deviation of the volatile compound content; “#N/A” means the missing concentration.

Table S6 The contents and ROAVs of aroma compounds of IPETs

| NO. | Compounds | Retention time | CAS ^a^ | OTs | Aroma description | Chemical formula | RI | NIST RI | The contents of aroma compounds (ug/kg) ^b^ | | | | | | | | | ROAV ^b^ | | | | | | | | | Reference^c^ |
| --- | --- | --- | --- | --- | --- | --- | --- | --- | --- | --- | --- | --- | --- | --- | --- | --- | --- | --- | --- | --- | --- | --- | --- | --- | --- | --- | --- |
|  |  |  |  |  |  |  |  |  | IPET-1 | IPET-2 | IPET-3 | IPET-4 | IPET-5 | IPET-6 | IPET-7 | IPET-8 | IPET-9 | IPET-1 | IPET-2 | IPET-3 | IPET-4 | IPET-5 | IPET-6 | IPET-7 | IPET-8 | IPET-9 |  |
| 1 | Ethyl hexanoate | 13.66 | 123-66-0 | 0.005 | Fruity, pineapple-like, sweet, green, waxy | C8H16O2 | 988 | 999-S | 2.12±0.18 | #N/A | #N/A | 20.32±9.93 | #N/A | #N/A | 9.02±0.54 | #N/A | #N/A | 423.89 | ＜1 | ＜1 | 4063.29 | ＜1 | ＜1 | 1804.45 | ＜1 | ＜1 | Yang et al., 2022 |
| 2 | Octanoic acid,ethyl ester | 20.99 | 106-32-1 | 650 | Fruity | C10H20O2 | 1187 | 1196-S | 8.09±1.22 | #N/A | #N/A | 34.56±18.91 | 2.33±1.28 | #N/A | 19.46±1.03 | 3.14±0.44 | #N/A | ＜1 | ＜1 | ＜1 | ＜1 | ＜1 | ＜1 | ＜1 | ＜1 | ＜1 | Huang et al., 2022 |
| 3 | Methyl salicylate | 20.93 | 119-36-8 | 40 | Green | C8H8O3 | 1186 | 1192-S | 33.28±2.7 | 29.28±3.51 | 22.1±0.76 | 51.15±10.95 | 14.96±0.3 | 23.23±2.1 | 53.92±1.64 | 35.54±3.27 | 26.01±1.11 | ＜1 | ＜1 | ＜1 | 1.28 | ＜1 | ＜1 | 1.35 | ＜1 | ＜1 | Guo, Ho, Schwab, & Wan, 2021a |
| 4 | Ethyl nonanoate | 24.39 | 123-29-5 | 0.377 | Fruity, apple, wine | C11H22O2 | 1285 | 1295-S | 8.28±0.3 | 1.65±0.22 | #N/A | 51.16±24.69 | 1.54±0.12 | #N/A | 38.5±3.31 | 2.13±0.17 | #N/A | 21.97 | 4.37 | ＜1 | 135.71 | 4.08 | ＜1 | 102.11 | 5.64 | ＜1 | Jiang et al., 2021a |
| 5 | dihydroactinidiolide | 32.13 | 17092-92-1 | 2.1 | Woody | C11H16O2 | 1527 | 1532-S | 180.91±7.59 | 184.94±3.18 | 161.74±9.57 | 170.67±13.66 | 209.96±37.96 | 203.56±42.06 | 176.28±8.22 | 192.17±5.2 | 219.5±7.94 | 86.15 | 88.07 | 77.02 | 81.27 | 99.98 | 96.93 | 83.94 | 91.51 | 104.52 | Zhu, Niu, &Xiao, 2021 |
| 6 | Methyl hexanoate | 10.94 | 106-70-7 | 4 | Pineapple | C7H14O2 | 913 | 925-S | #N/A | #N/A | #N/A | 1.76±0.78 | #N/A | #N/A | 0.87±0 | #N/A | #N/A | ＜1 | ＜1 | ＜1 | ＜1 | ＜1 | ＜1 | ＜1 | ＜1 | ＜1 | Sun et al., 2024 |
| 7 | Methyl caprate | 25.34 | 110-42-9 | 4.3 | Nicotian | C11H22O2 | 1312 | 1326-S | #N/A | #N/A | #N/A | 3.87±1.87 | #N/A | #N/A | 3±0.28 | #N/A | #N/A | ＜1 | ＜1 | ＜1 | ＜1 | ＜1 | ＜1 | ＜1 | ＜1 | ＜1 | Qi et al., 2020 |
| 8 | 1,2-Dimethoxybenzene | 19.13 | 91-16-7 | 3.17 | Stale | C8H10O2 | 1137 | 1148-S | 144.5±11.33 | 205.87±10.19 | 162.93±4 | 154.92±20.8 | 149.3±6.64 | 212.99±15.32 | 170.78±5.1 | 211.58±0.97 | 209.89±1.52 | 45.58 | 64.94 | 51.4 | 48.87 | 47.1 | 67.19 | 53.87 | 66.74 | 66.21 | Deng et al., 2021 |
| 9 | 3,4-Dimethoxytoluene | 22.46 | 494-99-5 | 5 | Stale | C9H12O2 | 1230 | 1233-S | 106.95±9.17 | 142.01±13.27 | 117.21±2.16 | 122.4±21.08 | 114.6±9.95 | 143.85±10.68 | 133.77±3.93 | 167.46±3.32 | 158.55±2.34 | 21.39 | 28.4 | 23.44 | 24.48 | 22.92 | 28.77 | 26.75 | 33.49 | 31.71 | Ma et al., 2022 |
| 10 | 1,2,3-Trimethoxybenzene | 25.06 | 634-36-6 | 0.75 | Stale | C9H12O3 | 1304 | 1313-S | 512.63±26.13 | 525.8±20.52 | 467.93±15.23 | 474.47±41.22 | 479.44±8.02 | 542.86±25.02 | 494.97±8.53 | 542.85±14.59 | 559.4±6.06 | 683.5 | 701.07 | 623.91 | 632.62 | 639.26 | 723.81 | 659.96 | 723.8 | 745.86 | Xu et al., 2021 |
| 11 | 4-Ethyl-1,2-dimethoxybenzene | 25.33 | 5888-51-7 | 3.4 | Stale | C10H14O2 | 1313 | 1320-S | 133.96±9.3 | 168.56±18.02 | 138.12±4 | 139.59±23.33 | 136.63±10.06 | 178.37±14.93 | 166.91±7.07 | 189.73±7.12 | 185.57±2.95 | 39.4 | 49.58 | 40.62 | 41.05 | 40.18 | 52.46 | 49.09 | 55.8 | 54.58 | Xu et al., 2021 |
| 12 | 1,2,4-Trimethoxybenzene | 26.99 | 135-77-3 | 3.06 | Stale, musty, herbal | C9H12O3 | 1362 | 1372-S | 364.52±14.39 | 324±16.99 | 309.83±11.29 | 336.43±27.89 | 342.48±3.83 | 317.98±21.08 | 353.64±5.29 | 335.31±9.27 | 336.48±3.41 | 119.13 | 105.88 | 101.25 | 109.95 | 111.92 | 103.91 | 115.57 | 109.58 | 109.96 | Xu et al., 2021 |
| 13 | 1,2,3-Trimethoxy-5-methylbenzene | 27.99 | 6443-69-2 | 4.45 | Stale | C10H14O3 | 1393 | 1407-S | 213.99±12.48 | 206.63±11.65 | 184.2±5.57 | 198.23±22.84 | 198.75±14.61 | 214.21±13.88 | 215.08±5.37 | 229.09±6.82 | 239.88±4.96 | 48.09 | 46.43 | 41.39 | 44.55 | 44.66 | 48.14 | 48.33 | 51.48 | 53.91 | Wang et al., 2022a |
| 14 | Methyl isoeugenol | 30.86 | 93-16-3 | 1600 | Blossom, carnation, woody | C11H14O2 | 1486 | 1492-S | 5.02±0.54 | 3.37±0.29 | 4.36±2.04 | 3.68±0.83 | 3.99±1.01 | 56.48±81.73 | 7.56±2.98 | 5.16±2.49 | 6.12±2.29 | ＜1 | ＜1 | ＜1 | ＜1 | ＜1 | ＜1 | ＜1 | ＜1 | ＜1 | Guo et al., 2021c |
| 15 | Elemicin | 32.66 | 487-11-6 | 22000 | Spice, flower | C12H16O3 | 1547 | 1554-S | 10.65±0.44 | 8.84±4.01 | 9.76±0.65 | 11.9±0.51 | 10.78±3.52 | 8.82±4.53 | 9.61±0.93 | 11.33±0.58 | 15.42±1.5 | ＜1 | ＜1 | ＜1 | ＜1 | ＜1 | ＜1 | ＜1 | ＜1 | ＜1 | Huang et al., 2022 |
| 16 | 1,3-Dimethoxybenzene | 19.94 | 151-10-0 | 100 | Stale | C8H10O2 | 1159 | 1168-S | #N/A | 3.94±0.97 | 3.05±0.15 | 3.42±2.2 | 4.77±2.25 | #N/A | #N/A | 4.67±0.04 | 4.09±1.2 | ＜1 | ＜1 | ＜1 | ＜1 | ＜1 | ＜1 | ＜1 | ＜1 | ＜1 | Ma et al., 2022 |
| 17 | Theaspirane | 25.21 | 36431-72-8 | 0.0002 | Honey | C13H22O | 1309 | 1302-S | #N/A | 1.73±0.07 | 2.2±0.62 | 1.84±0.03 | #N/A | 2.11±0.82 | 3.19±0.92 | 2.61±0.92 | 2.05±0.05 | ＜1 | 8640.75 | 11007.91 | 9185.68 | ＜1 | 10544.41 | 15937.14 | 13061.83 | 10274.48 | Guo, Ho, Schwab, & Wan, 2021a |
| 18 | Estragole | 21.06 | 140-67-0 | 7.5 | Anise | C10H12O | 1190 | 1196-S | #N/A | #N/A | #N/A | #N/A | #N/A | #N/A | #N/A | 5.32±0.47 | #N/A | ＜1 | ＜1 | ＜1 | ＜1 | ＜1 | ＜1 | ＜1 | ＜1 | ＜1 | Jin et al., 2021 |
| 19 | 2-Heptanone | 9.73 | 110-43-0 | 65 | Woody, fruity | C7H14O | 877 | 891-S | 3.98±0.48 | 8.73±3.96 | 6.22±2.06 | 5.27±1.59 | 3.84±1.02 | 8.32±1.97 | 5.76±1.4 | 9.28±0.71 | 7.16±0.2 | ＜1 | ＜1 | ＜1 | ＜1 | ＜1 | ＜1 | ＜1 | ＜1 | ＜1 | Xiao et al., 2023 |
| 20 | 6-Methyl-5-hepten-2-one | 13.19 | 110-93-0 | 0.068 | Fruity, apple-like, musty | C8H14O | 975 | 986-S | 7.08±0.79 | 28.12±0.44 | 13.94±0.79 | 18.01±3.71 | 10.43±1.71 | 26.75±5.31 | 17.55±0.62 | 32.6±1.24 | 22.71±0.92 | 104.12 | 413.49 | 204.94 | 264.84 | 153.4 | 393.33 | 258.08 | 479.39 | 333.94 | Yang et al., 2023 |
| 21 | 2-octanone | 13.39 | 111-13-7 | 0.005 | Fat, Fragrant, Mold | C8H16O | 980 | 991-S | 3.12±0.12 | 7.18±3.25 | 4.43±1.42 | 5.42±4.2 | #N/A | 7.72±2.72 | 5.25±1 | 10.3±0.15 | 8.38±1.07 | 623 | 1435.64 | 885.93 | 1083.94 | ＜1 | 1544.27 | 1050.18 | 2059.38 | 1675.25 | Yang et al., 2023 |
| 22 | 2,2,6-trimethylcyclohexanone | 15.06 | 2408-37-9 | 0.1 | Pungent, honey-like, citrus | C9H16O | 1026 | 1036-S | 7.45±0.86 | 18.61±6.33 | 12.59±1.21 | 13.81±4.48 | 7.14±1.38 | 18.9±3.61 | 16.43±0.47 | 25.49±1.13 | 22.47±2.09 | 74.52 | 186.14 | 125.89 | 138.05 | 71.4 | 188.96 | 164.33 | 254.88 | 224.74 | Guo et al., 2022 |
| 23 | Acetophenone | 16.19 | 98-86-2 | 65 | Sweet, cherry-like, vanilla-like | C8H8O | 1057 | 1066-S | 9.84±0.13 | 31.69±1.51 | 24.44±1.42 | 20.06±2.68 | 16.27±2.31 | 38.59±1.08 | 19.43±0.31 | 35.61±1.42 | 37.81±1.74 | ＜1 | ＜1 | ＜1 | ＜1 | ＜1 | ＜1 | ＜1 | ＜1 | ＜1 | Guo, Ho, Schwab, & Wan, 2021a |
| 24 | (E,E)-3,5-Octadien-2-one | 16.33 | 30086-02-3 | 0.15 | Creamy, fruity | C8H12O | 1060 | 1073-S | 27.37±3.12 | 108.59±15.01 | 81.35±25.32 | 120.15±99.2 | 46.34±3.87 | 120.55±7.94 | 73.32±2.56 | 108.17±52.44 | 127.19±7.87 | 182.47 | 723.93 | 542.3 | 800.98 | 308.93 | 803.7 | 488.83 | 721.13 | 847.93 | Xiao et al., 2018 |
| 25 | 6-Methyl-3,5-heptadiene-2-one | 17.59 | 1604-28-0 | 68 | Spice | C8H12O | 1094 | 1107-S | 5.7±1.14 | 17.67±1.85 | 13.37±1.69 | 11.3±4.96 | 9.46±1.76 | 20.46±1.78 | 11.93±2.03 | 23.96±4.77 | 24.34±1.8 | ＜1 | ＜1 | ＜1 | ＜1 | ＜1 | ＜1 | ＜1 | ＜1 | ＜1 | Ma et al., 2023 |
| 26 | Isophorone | 18.26 | 78-59-1 | 11 | Cooling, woody, sweet, green, fruity | C9H14O | 1112 | 1123-S | 5.07±0.4 | 21.63±1.81 | 17.42±0.65 | 12.81±3.46 | 8.9±0.4 | 27.8±1.12 | 12.88±1.11 | 25.99±1.33 | 26.57±1.39 | ＜1 | 1.97 | 1.58 | 1.16 | ＜1 | 2.53 | 1.17 | 2.36 | 2.42 | Guo, Ho, Schwab, & Wan, 2021a |
| 27 | 3-Nonen-2-one | 18.86 | 14309-57-0 | 800 | Fruity | C9H16O | 1128 | 1142-S | 3.15±0.31 | 11.1±2.57 | #N/A | 6.25±3.21 | 5.4±0.63 | #N/A | 6.8±1.16 | 14.65±0.37 | 19.12±1.86 | ＜1 | ＜1 | ＜1 | ＜1 | ＜1 | ＜1 | ＜1 | ＜1 | ＜1 | Wang et al., 2022b |
| 28 | Camphor | 19.19 | 76-22-2 | 460 | Camphor | C10H16O | 1138 | 1145-S | 3.68±0.42 | 10.15±1.57 | 7.32±0.51 | #N/A | 5.15±0.57 | 11.89±1.4 | #N/A | #N/A | 11.57±0.2 | ＜1 | ＜1 | ＜1 | ＜1 | ＜1 | ＜1 | ＜1 | ＜1 | ＜1 | Zhu et al., 2015 |
| 29 | 2-Undecanone | 24.33 | 112-12-9 | 450 | Waxy, fruity, creamy, fatty, orris, floral | C11H22O | 1283 | 1294-S | 4.09±0.4 | 4.39±1.57 | 5.38±0.51 | #N/A | 3.82±0.26 | 6.16±0.35 | #N/A | 7.41±0.97 | 7.97±1.48 | ＜1 | ＜1 | ＜1 | ＜1 | ＜1 | ＜1 | ＜1 | ＜1 | ＜1 | Jiang et al., 2021b |
| 30 | (Z)-Jasmone | 27.86 | 488-10-8 | 0.007 | Floral, woody, herbal, citrus, jasmine-like | C11H16O | 1389 | 1395-S | 1.61±0.11 | 5.35±0.62 | 4.04±0.99 | 2.56±0.37 | 3.15±0.61 | 7.05±1.47 | 3.85±1.1 | 6.9±0.48 | 7.84±0.06 | 229.99 | 763.65 | 577.81 | 366.3 | 450.24 | 1007.18 | 550.53 | 985.53 | 1119.73 | Yang et al., 2021 |
| 31 | α-ionone | 28.79 | 127-41-3 | 0.0004 | Floral, violet-like, powdery, berry-like | C13H20O | 1418 | 1426-S | 28.32±1.91 | 57.63±9.31 | 55.16±1.45 | 46.94±10.89 | 42.97±3.27 | 73.07±10.19 | 66.27±6.04 | 82.82±6.74 | 79.06±1.24 | 70810.01 | 144073.94 | 137909.03 | 117347.39 | 107421.2 | 182670.66 | 165668.08 | 207043.77 | 197649.73 | Yang et al., 2021 |
| 32 | (E)-Geranylacetone | 29.53 | 3796-70-1 | 60 | Fresh, rose-like, floral, green, fruity | C13H22O | 1442 | 1453-S | 24.37±2.51 | 43.65±8.76 | #N/A | 29.62±16.6 | 30.42±0.22 | #N/A | 47.91±0.77 | 70.19±3.49 | 63.27±3.13 | ＜1 | ＜1 | ＜1 | ＜1 | ＜1 | ＜1 | ＜1 | 1.17 | 1.05 | Guo et al., 2022 |
| 33 | γ-Ionone | 30.59 | 14901-07-6 | 100 | Woody | C13H20O | 1477 | 1491-S | 83.02±6.48 | 162.75±24.4 | 133.34±12.3 | 123.47±25.61 | 116.33±15.13 | 167.97±18.17 | #N/A | 192.14±9.89 | 204.23±17.96 | ＜1 | 1.63 | 1.33 | 1.23 | 1.16 | 1.68 | ＜1 | 1.92 | 2.04 | Li et al., 2024 |
| 34 | 1-(4-Methylphenyl)ethanone | 20.14 | 122-00-9 | 21 | Powdery, vanilla-like, floral | C9H10O | 1164 | 1183-S | #N/A | 3.33±0.85 | #N/A | #N/A | #N/A | #N/A | #N/A | 4.77±0.26 | 5.45±0.17 | ＜1 | ＜1 | ＜1 | ＜1 | ＜1 | ＜1 | ＜1 | ＜1 | ＜1 | Guo et al., 2021c |
| 35 | Oxopholone | 19.08 | 1125-21-9 | 25 | Musty, woody, sweet, tea-like, citrus | C9H12O2 | 1135 | 1145-S | #N/A | #N/A | 9.4±0.53 | 7.11±1.94 | 5.22±0.49 | 16.53±0.34 | 6.81±0.15 | 14.4±0.28 | 15.9±0.49 | ＜1 | ＜1 | ＜1 | ＜1 | ＜1 | ＜1 | ＜1 | ＜1 | ＜1 | Guo et al., 2021c |
| 36 | 1-(2-methylphenyl)ethanone | 20.14 | 577-16-2 | 200 | Floral | C9H10O | 1164 | 1173-S | #N/A | #N/A | 4.26±0.03 | #N/A | #N/A | #N/A | #N/A | #N/A | #N/A | ＜1 | ＜1 | ＜1 | ＜1 | ＜1 | ＜1 | ＜1 | ＜1 | ＜1 | Jiang et al., 2023 |
| 37 | β-Damascenone | 27.48 | 23726-93-4 | 10 | Floral, sweet | C13H18O | 1377 | 1386-S | #N/A | #N/A | 2.89±0.95 | #N/A | #N/A | 3.36±1.26 | #N/A | 1.33±0.09 | 3.02±1.45 | ＜1 | ＜1 | ＜1 | ＜1 | ＜1 | ＜1 | ＜1 | ＜1 | ＜1 | Xiao et al., 2018 |
| 38 | Geranyl acetone | 29.55 | 689-67-8 | 60 | Fresh, rose-like, floral, green, fruit | C13H22O | 1442 | 1452-S | #N/A | #N/A | 35.38±2.22 | 10±5.96 | #N/A | 48.11±7.3 | #N/A | #N/A | #N/A | ＜1 | ＜1 | ＜1 | ＜1 | ＜1 | ＜1 | ＜1 | ＜1 | ＜1 | Liu et al., 2021b |
| 39 | 3-Methylacetophenone | 20.14 | 585-74-0 | 1990 | Powdery, sweet, pungent, fruity nuances | C9H10O | 1164 | 1182-S | #N/A | #N/A | #N/A | 3.01±0.25 | 2.73±0.16 | #N/A | 2.35±0.28 | #N/A | #N/A | ＜1 | ＜1 | ＜1 | ＜1 | ＜1 | ＜1 | ＜1 | ＜1 | ＜1 | Guo et al., 2022 |
| 40 | Styrene | 9.73 | 100-42-5 | 65 | Sweet | C8H8 | 877 | 893-S | 1.62±0.14 | 4.72±3.57 | 2.57±0.17 | 6.44±3.93 | 4.1±2.07 | 8.94±4.12 | 7.56±1.17 | 9.09±2.09 | 3.58±0.19 | ＜1 | ＜1 | ＜1 | ＜1 | ＜1 | ＜1 | ＜1 | ＜1 | ＜1 | Xu et al., 2021 |
| 41 | β-myrcene | 13.39 | 123-35-3 | 0.0012 | Lemon, woody, floral | C10H16 | 980 | 991-S | 1.43±0.09 | 1.16±0.01 | 1.33±0.07 | #N/A | #N/A | 1.1±0.18 | 1.12±0.17 | 1.01±0.05 | 1.07±0.04 | 1189.62 | 965.12 | 1105.17 | ＜1 | ＜1 | 918.47 | 930.13 | 845.12 | 888.8 | Yang et al., 2023 |
| 42 | (E)-β-Ocimene | 15.13 | 3779-61-1 | 0.0187 | Warm, floral, herbal, sweet | C10H16 | 1027 | 1049-S | 1.61±0.17 | 1.49±0.08 | 1.71±0.05 | 2.11±0.83 | #N/A | 1.68±0.07 | 1.98±0.96 | 1.41±0.07 | 1.67±0.08 | 85.85 | 79.5 | 91.26 | 113.06 | ＜1 | 89.82 | 105.74 | 75.47 | 89.43 | Guo, Ho, Schwab, & Wan, 2021a |
| 43 | (Z)-β-Ocimene | 15.13 | 3338-55-4 | 10 | Citrus, herbal, spicy, sweet | C10H16 | 1027 | 1038-S | 2.36±0.88 | 1.76±0.19 | 2.23±0.05 | #N/A | #N/A | #N/A | #N/A | #N/A | #N/A | ＜1 | ＜1 | ＜1 | ＜1 | ＜1 | ＜1 | ＜1 | ＜1 | ＜1 | Guo et al., 2022 |
| 44 | γ-Terpinene | 15.93 | 99-85-4 | 55 | Citrus, lemon-like, woody, spicy, juicy | C10H16 | 1049 | 1060-S | 1.67±0.04 | 1.58±0.04 | 2.42±0.05 | 1.65±0.1 | #N/A | 2.73±0.01 | #N/A | #N/A | #N/A | ＜1 | ＜1 | ＜1 | ＜1 | ＜1 | ＜1 | ＜1 | ＜1 | ＜1 | Guo et al., 2022 |
| 45 | Terpinolene | 17.06 | 586-62-9 | 0.2 | Fresh, woody, sweet, piney, citrus | C10H16 | 1079 | 1088-S | 1.78±0.37 | #N/A | #N/A | #N/A | #N/A | #N/A | #N/A | #N/A | #N/A | 8.88 | ＜1 | ＜1 | ＜1 | ＜1 | ＜1 | ＜1 | ＜1 | ＜1 | Guo, Ho, Schwab, & Wan, 2021a |
| 46 | Undecane | 17.39 | 1120-21-4 | 10 | Alkane | C11H24 | 1088 | 1100-A | 1.3±0.08 | 0.93±0.17 | 0.85±0.08 | #N/A | #N/A | #N/A | #N/A | #N/A | #N/A | ＜1 | ＜1 | ＜1 | ＜1 | ＜1 | ＜1 | ＜1 | ＜1 | ＜1 | Guo, Ho, Schwab, & Wan, 2021b |
| 47 | Naphthalene | 20.53 | 91-20-3 | 6 | Pungent, tarry-like | C10H8 | 1175 | 1182-S | 31.57±2.78 | 52.28±15.27 | 46.85±2.15 | 39.78±7.91 | 35.27±2.44 | 60.4±0.13 | 46.86±1.24 | 60.69±2.12 | 62.28±0.95 | 5.26 | 8.71 | 7.81 | 6.63 | 5.88 | 10.07 | 7.81 | 10.11 | 10.38 | Guo et al., 2022 |
| 48 | Dodecane | 21.06 | 112-40-3 | 10000 | Alkane-like | C12H26 | 1189 | 1200-A | 3.39±0.28 | 2.02±0.14 | 2.77±0.43 | 2.12±1.36 | 1.71±1.24 | 1.47±0.16 | 1±0.05 | 1.4±0.09 | 1.54±0.69 | ＜1 | ＜1 | ＜1 | ＜1 | ＜1 | ＜1 | ＜1 | ＜1 | ＜1 | Guo, Ho, Schwab, & Wan, 2021a |
| 49 | 1-Methylnaphthalene | 24.39 | 90-12-0 | 8 | Naphthyl, chemical, medicinal, camphoreous | C11H10 | 1286 | 1307-S | 9.34±0.97 | 11.08±4 | 14.17±0.53 | 11±3.39 | 9.91±0.43 | 16.04±1.92 | #N/A | 17.15±0.01 | #N/A | 1.17 | 1.38 | 1.77 | 1.38 | 1.24 | 2 | ＜1 | 2.14 | ＜1 | Guo et al., 2022 |
| 50 | Tridecane | 24.53 | 629-50-5 | 42 | Alkane | C13H28 | 1289 | 1300-A | 2.54±0.09 | 3.39±0.41 | 4.73±2.39 | 3.02±2.6 | 3.38±3.61 | 1.82±0.07 | 1.12±0.08 | 1.42±0.15 | 2.16±1.22 | ＜1 | ＜1 | ＜1 | ＜1 | ＜1 | ＜1 | ＜1 | ＜1 | ＜1 | Guo, Ho, Schwab, & Wan, 2021a |
| 51 | Dehydro-ar-ionene | 26.46 | 30364-38-6 | 2.5 | Licorice-like | C13H16 | 1347 | 1354-S | 4.91±0.88 | 7.59±2.81 | 14.7±0.47 | 4.84±1.94 | 5.3±2.04 | 14.69±0.77 | 6.93±0.85 | 10.02±0.97 | 15.55±1.39 | 1.96 | 3.04 | 5.88 | 1.94 | 2.12 | 5.88 | 2.77 | 4.01 | 6.22 | Guo, Ho, Schwab, & Wan, 2021a |
| 52 | 3-Methyltridecane | 26.86 | 6418-41-3 | 42 | Alkane | C14H30 | 1358 | 1371-S | 1.94±1.15 | #N/A | 3.67±0.09 | 3.27±0.26 | #N/A | #N/A | 1.34±0.27 | 2.42±1.51 | 2.25±1.41 | ＜1 | ＜1 | ＜1 | ＜1 | ＜1 | ＜1 | ＜1 | ＜1 | ＜1 | Fang et al., 2023 |
| 53 | Tetradecane | 27.79 | 629-59-4 | 10000 | Alkane | C14H30 | 1386 | 1400-A | 3.33±0.18 | 7.06±1.98 | 6.27±2.2 | 5.14±2.93 | 4.94±1.86 | 4.36±0.73 | 3.14±0.35 | 4.53±0.31 | 5.97±2.21 | ＜1 | ＜1 | ＜1 | ＜1 | ＜1 | ＜1 | ＜1 | ＜1 | ＜1 | Guo, Ho, Schwab, & Wan, 2021a |
| 54 | Longifolene | 28.26 | 475-20-7 | 2 | Woody | C15H24 | 1401 | 1406-S | 1.65±0.18 | 2.19±0.09 | 3.28±0.08 | #N/A | 2.12±0.55 | 3.23±0.29 | 1.08±0.08 | 1.9±0.07 | 2.36±0.07 | ＜1 | 1.1 | 1.64 | ＜1 | 1.06 | 1.62 | ＜1 | ＜1 | 1.18 | Fang et al., 2023 |
| 55 | 1,7-Dimethyl-naphthalene | 28.53 | 575-37-1 | 0.04 | Aromatic | C12H12 | 1410 | 1419-S | 2.78±0.44 | 8.45±0.01 | 5.26±0.88 | 3.27±1.14 | 3.68±0.84 | 7.56±2.63 | 5.07±1.07 | 8.51±1.09 | 6.98±0.14 | 69.55 | 211.18 | 131.48 | 81.78 | 92.07 | 189.11 | 126.68 | 212.73 | 174.44 | Guo, Ho, Schwab, & Wan, 2021b |
| 56 | Hexadecane | 33.86 | 544-76-3 | 13000000 | Alkane | C16H34 | 1586 | 1600-A | 4.07±0.27 | 6.13±2.29 | 5.85±1.33 | 6.28±3.14 | 8.24±4.83 | 4.79±0.76 | 7.2±5.29 | 6.49±4.28 | 7.61±6.83 | ＜1 | ＜1 | ＜1 | ＜1 | ＜1 | ＜1 | ＜1 | ＜1 | ＜1 | Guo, Ho, Schwab, & Wan, 2021a |
| 57 | Heptadecane | 36.59 | 629-78-7 | 10000000 | Alkane | C17H36 | 1688 | 1700-A | 2.24±0.82 | 3.02±1.13 | 2.78±0.9 | 2.3±0.35 | 1.81±0.48 | 3.23±1.98 | 1.93±0.45 | 1.35±0.1 | 1.67±0.35 | ＜1 | ＜1 | ＜1 | ＜1 | ＜1 | ＜1 | ＜1 | ＜1 | ＜1 | Guo, Ho, Schwab, & Wan, 2021a |
| 58 | 1,2,3-Trimethylbenzene | 14.54 | 526-73-8 | 260 | Plastic-like | C9H12 | 1011 | 1013-S | #N/A | 5.27±0.32 | 4.65±0.28 | #N/A | 1.72±0.08 | 7.16±0.93 | #N/A | #N/A | 5.94±0.36 | ＜1 | ＜1 | ＜1 | ＜1 | ＜1 | ＜1 | ＜1 | ＜1 | ＜1 | Guo et al., 2022 |
| 59 | 2-Methylnaphthalene | 24.41 | 91-57-6 | 3 | Tar, spicy | C11H10 | 1286 | 1297-S | #N/A | 13.83±3.84 | #N/A | #N/A | #N/A | #N/A | #N/A | #N/A | 17.25±0.44 | ＜1 | 4.61 | ＜1 | ＜1 | ＜1 | ＜1 | ＜1 | ＜1 | 5.75 | Wen et al., 2023 |
| 60 | 3-Carene | 15.55 | 13466-78-9 | 50 | Woody | C10H16 | 1038 | 1011-S | #N/A | #N/A | #N/A | #N/A | 1.37±0.12 | 8.98±6.97 | #N/A | #N/A | 2.11±0.08 | ＜1 | ＜1 | ＜1 | ＜1 | ＜1 | ＜1 | ＜1 | ＜1 | ＜1 | Fang et al., 2023 |
| 61 | Toluene | 6.06 | 108-88-3 | 527 | Sweet, aromatic | C7H8 | 741 | 763-S | #N/A | #N/A | #N/A | #N/A | #N/A | 1.36±0.02 | #N/A | #N/A | #N/A | ＜1 | ＜1 | ＜1 | ＜1 | ＜1 | ＜1 | ＜1 | ＜1 | ＜1 | Guo, Ho, Schwab, & Wan, 2021a |
| 62 | Ethylbenzene | 8.79 | 100-41-4 | 2205.25 | Aromatic odor | C8H10 | 847 | 850-N | #N/A | #N/A | #N/A | #N/A | #N/A | 1.49±0.03 | 0.98±0.02 | 6.7±1.45 | #N/A | ＜1 | ＜1 | ＜1 | ＜1 | ＜1 | ＜1 | ＜1 | ＜1 | ＜1 | Guo, Ho, Schwab, & Wan, 2021a |
| 63 | 1,2,4-Trimethylbenzene | 14.59 | 95-63-6 | 260 | Plastic | C9H12 | 1013 | 990-S | #N/A | #N/A | #N/A | #N/A | #N/A | #N/A | 4.53±0.57 | 6.43±0.01 | #N/A | ＜1 | ＜1 | ＜1 | ＜1 | ＜1 | ＜1 | ＜1 | ＜1 | ＜1 | Guo et al., 2022 |
| 64 | Hexanoic acid | 12.73 | 142-62-1 | 1840 | Sweat, pungent, cheese | C6H12O2 | 962 | 990-S | 1.58±0.34 | 4.13±0.08 | 2.38±0 | #N/A | 2.47±0.86 | 4.7±0.11 | #N/A | 6.87±1.82 | 5.73±0.96 | ＜1 | ＜1 | ＜1 | ＜1 | ＜1 | ＜1 | ＜1 | ＜1 | ＜1 | Yang et al., 2022 |
| 65 | Nonanoic acid | 23.33 | 112-05-0 | 3000 | Green, spicy | C9H18O2 | 1254 | 1273-S | 2.1±0.79 | 2.19±0.35 | 2.09±0.65 | #N/A | 2.53±1.13 | 3.93±0.21 | 2.34±0.54 | 2.84±0.73 | 4.05±0.55 | ＜1 | ＜1 | ＜1 | ＜1 | ＜1 | ＜1 | ＜1 | ＜1 | ＜1 | Rigling et al., 2021 |
| 66 | Hexanal | 6.86 | 66-25-1 | 0.005 | Grassy, green, fresh, fatty | C6H12O | 779 | 801-S | 39.66±2.17 | 30.64±10.42 | 17.44±0.92 | 38.49±7.72 | 19.74±3.95 | 18.61±3.53 | 39.72±2.11 | 31.07±3.27 | 21.22±0.51 | 7932.04 | 6128.58 | 3488.73 | 7697.64 | 3948.35 | 3721.27 | 7943.95 | 6214.34 | 4243.91 | Xiao, Cao, Zhu, Chen, & Niu, 2022 |
| 67 | (E)-2-Hexenal | 8.53 | 6728-26-3 | 17 | Green, leafy, fruity | C6H10O | 839 | 854-S | 7.58±0.69 | 3.13±1.29 | 1.81±0.05 | 5.52±0.67 | 3.36±0.8 | 1.52±0.02 | 5.16±0.28 | 3.21±1.18 | 1.72±0.06 | ＜1 | ＜1 | ＜1 | ＜1 | ＜1 | ＜1 | ＜1 | ＜1 | ＜1 | Guo et al., 2022 |
| 68 | (Z)-4-Heptenal | 10.06 | 6728-31-0 | 0.025 | Fatty, oily, green, creamy | C7H12O | 888 | 900-S | 8.68±0.44 | 8.31±0.48 | #N/A | 8.49±1.35 | 6.49±0.65 | #N/A | 8.98±0.88 | 7.59±0.69 | 5.91±0.26 | 347.04 | 332.21 | ＜1 | 339.41 | 259.56 | ＜1 | 359.06 | 303.77 | 236.33 | Pang et al., 2019 |
| 69 | Heptanal | 10.13 | 111-71-7 | 0.033 | Heavy, plant green odor, apricot-like, and nutty aroma | C7H14O | 890 | 901-S | 18.06±1.46 | 13.77±6.53 | 10.52±4.14 | 15.18±4.41 | 9.45±3.59 | 8.54±1.87 | 17.46±1.24 | 17.57±3.93 | 11.65±0.73 | 547.22 | 417.2 | 318.93 | 460.08 | 286.48 | 258.84 | 529.03 | 532.49 | 353 | Guo et al., 2021c |
| 70 | benzaldehyde | 12.26 | 100-52-7 | 3 | Floral, almond-like | C7H6O | 950 | 962-S | 33.74±2.96 | 64.58±7.15 | 63.52±2.9 | 44.33±6.73 | 35.92±2.94 | 91.58±5.89 | 42.66±0.63 | 75.24±2.23 | 87.93±3.38 | 11.25 | 21.53 | 21.17 | 14.78 | 11.97 | 30.53 | 14.22 | 25.08 | 29.31 | Yang et al., 2022 |
| 71 | octanal | 13.79 | 124-13-0 | 0.00059 | Pungent, fruity and floral odor | C8H16O | 991 | 1003-S | 19.2±1.53 | 22.21±7.16 | 19.97±1.1 | 19.22±5.15 | 20.37±4.26 | #N/A | 18.57±3.41 | 23.89±8.66 | #N/A | 32540.8 | 37651.49 | 33855.21 | 32580.87 | 34524.46 | ＜1 | 31481.19 | 40499.56 | ＜1 | Yang et al., 2023 |
| 72 | (E,E)-2,4-Heptadienal | 14.13 | 4313/3/5 | 0.032 | Fatty, green, oily, cinnamon-like | C7H10O | 1001 | 1012-S | 14.53±0.53 | 24.46±9.45 | 10.15±0.62 | 16.13±4.75 | 30.36±4.35 | 14.72±3.1 | 11.58±8.04 | 31.93±24.49 | 23.43±0.91 | 454.06 | 764.38 | 317.19 | 504.06 | 948.75 | 460 | 361.88 | 997.81 | 732.19 | Flaig et al., 2020 |
| 73 | benzeneacetaldehyde | 15.39 | 122-78-1 | 0.0003 | Sweet and fragrant honey | C8H8O | 1035 | 1045-S | 30.64±3.5 | 29.48±1.49 | 35.82±2.14 | 26.55±3.57 | 28.97±3.49 | 37.43±1.96 | 24.62±1.64 | 31.54±2.35 | 38.5±5.64 | 102124.06 | 98275.47 | 119396.11 | 88483.55 | 96558.12 | 124766.44 | 82055.93 | 105143.78 | 128332.14 | Yang et al., 2023 |
| 74 | (E)-2-Octenal | 15.86 | 2548-87-0 | 3 | Fresh, cucumber-like, fatty, green, herbal, leafy | C8H14O | 1047 | 1060-S | 15.26±1.32 | 9.17±3.61 | 3.42±0.19 | 13.75±3.98 | 9.89±1.06 | 3.48±0.37 | 15.22±0.82 | 12.89±1.55 | 6.1±0.55 | 5.09 | 3.06 | 1.14 | 4.58 | 3.3 | 1.16 | 5.07 | 4.3 | 2.03 | Yang et al., 2022 |
| 75 | nonanal | 17.59 | 124-19-6 | 1.1 | Floral, fatty, green, lemon-like | C9H18O | 1093 | 1104-S | 109.08±9.85 | 101.83±5.21 | 96.62±14.63 | 130.66±4.68 | 110.39±18.93 | 88.76±7.85 | 101.69±3.87 | 106.71±5.39 | 105.13±4.6 | 99.17 | 92.57 | 87.84 | 118.78 | 100.35 | 80.69 | 92.44 | 97.01 | 95.58 | Guo et al., 2021c |
| 76 | (E,Z)-2,6-Nonadienal | 19.39 | 557-48-2 | 0.0045 | Green | C9H14O | 1143 | 1155-S | 17.93±1.16 | #N/A | #N/A | 19.48±5.44 | 17.32±7.06 | #N/A | 15.22±1.63 | #N/A | #N/A | 3983.73 | ＜1 | ＜1 | 4329.05 | 3848.28 | ＜1 | 3381.87 | ＜1 | ＜1 | Flaig et al., 2020 |
| 77 | (E)-2-Nonenal | 19.59 | 18829-56-6 | 0.08 | Green and tallow-like odor | C9H16O | 1149 | 1162-S | 25.58±2.48 | 15±3.73 | 7.18±0.57 | 25.63±5.52 | 15.89±1.16 | 9.08±1.62 | 28.68±1.28 | 19.39±1.07 | 12.73±1.88 | 319.78 | 187.45 | 89.75 | 320.37 | 198.58 | 113.44 | 358.48 | 242.42 | 159.1 | Yang et al., 2022 |
| 78 | 2,4-Dimethylbenzaldehyde | 20.19 | 15764-16-6 | 0.2 | Sweet, almond | C9H10O | 1166 | 1182-S | 9.04±1.83 | 13.34±5.36 | 16.21±0.62 | 10.78±3.33 | 10.95±0.63 | #N/A | 9.72±2.24 | 18.92±0.25 | 22.29±0.45 | 45.22 | 66.68 | 81.06 | 53.92 | 54.77 | ＜1 | 48.62 | 94.59 | 111.47 | Xiao et al., 2018 |
| 79 | Safranal | 21.13 | 116-26-7 | 0.7 | Woody, spicy, medicinal, powdery, and herbal | C10H14O | 1192 | 1201-S | 44.66±4.1 | 118.31±16.29 | 113.64±4.36 | 76.97±20.4 | 65.71±9.26 | 154.18±5.99 | 95.8±2.23 | 141.9±4.74 | 154.27±0.85 | 63.8 | 169.01 | 162.34 | 109.95 | 93.87 | 220.26 | 136.85 | 202.72 | 220.38 | Ni et al., 2021 |
| 80 | Decanal | 21.26 | 112-31-2 | 0.1 | Aldehyde-like, candle-like, fatty and citrus-like aroma | C10H20O | 1195 | 1206-S | 69.56±7.82 | 67.76±3.51 | 35.15±7.75 | 68.43±3.75 | 53.87±9.01 | 29.1±2.32 | 60.92±1.06 | 45.96±4.91 | 38.59±2.7 | 695.56 | 677.59 | 351.5 | 684.33 | 538.68 | 290.96 | 609.22 | 459.56 | 385.87 | Zhu et al., 2018 |
| 81 | (E,E)-2,4-Nonadienal | 21.59 | 5910-87-2 | 8 | Fatty, wax, green | C9H14O | 1205 | 1216-S | 5.02±1.24 | #N/A | #N/A | #N/A | #N/A | #N/A | 6.56±0.99 | #N/A | #N/A | ＜1 | ＜1 | ＜1 | ＜1 | ＜1 | ＜1 | ＜1 | ＜1 | ＜1 | Liu et al., 2021b |
| 82 | β-cyclocitral | 21.93 | 432-25-7 | 3 | Herbal, rose-like, fruity | C10H16O | 1214 | 1220-S | 24.38±2.2 | 55.7±9.81 | 51.33±2.37 | 39.36±13.87 | 29.66±5.39 | 74.11±3.08 | 40.28±11.46 | 71.08±4.1 | 82.25±3.69 | 8.13 | 18.57 | 17.11 | 13.12 | 9.89 | 24.7 | 13.43 | 23.69 | 27.42 | Guo et al., 2021c |
| 83 | Undecanal | 24.79 | 112-44-7 | 12.5 | Wax, floral | C11H22O | 1297 | 1307-S | 4.93±0.2 | 5.18±0.2 | 3.66±0.44 | 4.8±0.15 | 4.38±0.81 | 3.1±0.26 | 4.2±0.41 | 3.66±0.31 | 3.62±0.55 | ＜1 | ＜1 | ＜1 | ＜1 | ＜1 | ＜1 | ＜1 | ＜1 | ＜1 | Qi et al., 2020 |
| 84 | Dodecanal | 28.13 | 112-54-9 | 10 | Lilac, violet | C12H24O | 1396 | 1409-S | 8.36±0.32 | 10.58±2.34 | 13.79±0.68 | 5.59±0.3 | 14±0.64 | 12.28±1.18 | 5.22±0.85 | 8.12±0.43 | 12.64±0.38 | ＜1 | 1.06 | 1.38 | ＜1 | 1.4 | 1.23 | ＜1 | ＜1 | 1.26 | Qi et al., 2020 |
| 85 | Salicylaldehyde | 15.34 | 1990/2/8 | 0.03 | Almond | C7H6O2 | 1033 | 1047-S | #N/A | 3.51±0.3 | #N/A | #N/A | #N/A | #N/A | 2.43±0.09 | 3.72±0.37 | #N/A | ＜1 | 117.05 | ＜1 | ＜1 | ＜1 | ＜1 | ＜1 | ＜1 | ＜1 | Xiao, Cao, Zhu, Chen, & Niu, 2022 |
| 86 | (E,E)-2,4-Decadienal | 25.14 | 25152-84-5 | 0.01 | Oily, cucumber, fatty, fried | C10H16O | 1307 | 1317-S | #N/A | 13.62±4.07 | 15.28±2.52 | #N/A | 17.41±4.24 | 19.07±2.72 | #N/A | 24.49±2.01 | 26.53±0.03 | ＜1 | 1361.93 | 1528.02 | ＜1 | 1741.4 | 1907.49 | ＜1 | 2449.46 | 2653.26 | Xiao et al., 2018 |
| 87 | (E，Z)-2,4-Decadienal | 25.13 | 25152-83-4 | 0.07 | Fat, fish, flower, fried, green | C10H16O | 1307 | 1295-S | #N/A | #N/A | #N/A | #N/A | #N/A | #N/A | #N/A | #N/A | 16.14±11.74 | ＜1 | ＜1 | ＜1 | ＜1 | ＜1 | ＜1 | ＜1 | ＜1 | 230.61 | Yin et al., 2023 |
| 88 | Dimethyl trisulfide | 12.61 | 3658-80-8 | 0.01 | Bitter | C2H6S3 | 959 | 971-S | #N/A | 2.19±0.23 | 6.66±1.62 | #N/A | 1.43±0.21 | 5.6±0.65 | 2.03±0.32 | 2.52±0.52 | 4.35±0.68 | ＜1 | 218.67 | 666.47 | ＜1 | 143.22 | 559.51 | 203.38 | 251.65 | 435.5 | Liu et al., 2021a |
| 89 | Indole | 24.47 | 120-72-9 | 140 | Floral, animal-like | C8H7N | 1286 | 1295-S | #N/A | 4.58±0.25 | 9.11±0.34 | #N/A | 4.01±1.33 | 10.58±0.54 | #N/A | 4.66±0.34 | 11.22±0.75 | ＜1 | ＜1 | ＜1 | ＜1 | ＜1 | ＜1 | ＜1 | ＜1 | ＜1 | Zhu, Niu, & Xiao, 2021 |
| 90 | Dimethyl disulfide | 5.53 | 624-92-0 | 12 | Onion, cabbage, putrid | C2H6S2 | 716 | 730-N | #N/A | #N/A | #N/A | #N/A | #N/A | 1.7±0.12 | #N/A | #N/A | #N/A | ＜1 | ＜1 | ＜1 | ＜1 | ＜1 | ＜1 | ＜1 | ＜1 | ＜1 | Zhu, Niu, & Xiao, 2021 |
| 91 | Phenol | 12.93 | 108-95-2 | 1000 | Phenol | C6H6O | 968 | 981-S | 1.78±0.45 | 3.9±1.18 | 2.58±1.64 | 2.16±0.5 | 1.95±1.02 | 3.72±0.14 | 1.79±0.82 | 4.19±0.63 | 5.3±0.1 | ＜1 | ＜1 | ＜1 | ＜1 | ＜1 | ＜1 | ＜1 | ＜1 | ＜1 | Gong et al., 2017 |
| 92 | 2,4-Di-t-butylphenol | 31.33 | 96-76-4 | 0.5 | Chemical | C14H22O | 1501 | 1514-S | 49.16±5.26 | 59.19±17.7 | 76±7.47 | 39.84±17.35 | 39.22±4.73 | 58.31±3.05 | 56.86±5.94 | 62.13±6.35 | 64.85±2.3 | 98.32 | 118.38 | 152 | 79.68 | 78.44 | 116.62 | 113.73 | 124.26 | 129.71 | Fang et al., 2023 |
| 93 | Butylated hydroxytoluene | 31.39 | 128-37-0 | 1000 | Unpleasant odor | C15H24O | 1503 | 1513-S | 27.54±6.59 | #N/A | #N/A | 8.25±2.72 | #N/A | #N/A | #N/A | #N/A | #N/A | ＜1 | ＜1 | ＜1 | ＜1 | ＜1 | ＜1 | ＜1 | ＜1 | ＜1 | Liu et al., 2022 |
| 94 | Cedrol | 34.19 | 77-53-2 | 0.5 | Mild cedar wood-like aroma | C15H26O | 1599 | 1600-S | 6.73±0.12 | 10.11±1 | 6.01±2.05 | 7.23±1.12 | 7.56±0.95 | 8.47±1.27 | 12.32±9.38 | 8.87±0.96 | 11.12±1.32 | 13.46 | 20.22 | 12.03 | 14.46 | 15.12 | 16.94 | 24.65 | 17.75 | 22.23 | Zhu et al., 2018 |
| 95 | Carvacrol | 24.61 | 499-75-2 | 2290 | Herbal | C10H14O | 1291 | 1299-S | #N/A | 3.27±1.4 | #N/A | #N/A | #N/A | 3.69±0.27 | #N/A | 11.3±5.07 | 5.22±0.37 | ＜1 | ＜1 | ＜1 | ＜1 | ＜1 | ＜1 | ＜1 | ＜1 | ＜1 | Qi et al., 2020 |
| 96 | Thymol | 24.62 | 89-83-8 | 1700 | Herbal, medicinal, camphor, woody, spicy | C10H14O | 1291 | 1291-S | #N/A | #N/A | 3±1.35 | #N/A | #N/A | 6.4±0.68 | #N/A | 4.33±1.75 | 9.49±3.54 | ＜1 | ＜1 | ＜1 | ＜1 | ＜1 | ＜1 | ＜1 | ＜1 | ＜1 | Guo, Schwab, Ho, Song, & Wan, 2022 |
| 97 | 2-Ethylphenol | 19.86 | 90-00-6 | 300 | Sweet, cucumber | C8H10O | 1157 | 1140-S | #N/A | #N/A | #N/A | #N/A | #N/A | 3.11±1.26 | #N/A | #N/A | #N/A | ＜1 | ＜1 | ＜1 | ＜1 | ＜1 | ＜1 | ＜1 | ＜1 | ＜1 | Xu et al., 2021 |
| 98 | 3-Methylphenol | 15.66 | 108-39-4 | 0.8 | Pungent | C7H8O | 1042 | 1075-S | #N/A | #N/A | #N/A | #N/A | #N/A | #N/A | #N/A | 1.68±0.16 | 1.81±0.17 | ＜1 | ＜1 | ＜1 | ＜1 | ＜1 | ＜1 | ＜1 | 2.1 | ＜1 | Xiao et al., 2018 |
| 99 | 1-octen-3-ol | 12.93 | 3391-86-4 | 1 | Earthy, green, oily, vegetative-like, fungal | C8H16O | 968 | 980-S | 3.82±0.32 | 10.9±2 | 4.75±0.06 | 10.76±3.1 | 3.26±0.27 | 13.71±1.77 | 11.33±0.77 | 15.14±0.64 | 13.17±1.69 | 3.82 | 10.9 | 4.75 | 10.76 | 3.26 | 13.71 | 11.33 | 15.14 | 13.17 | Guo, Ho, Schwab, & Wan, 2021a |
| 100 | 2-Ethyl-1-hexanol | 14.73 | 104-76-7 | 270 | Sweet, floral | C8H18O | 1016 | 1030-S | 25.45±13.79 | 103.91±18.32 | 115±5.01 | 61.38±6.71 | 81.27±4.9 | 150.82±8.68 | 49.32±13.2 | 137.85±7.56 | 161.02±5.17 | ＜1 | ＜1 | ＜1 | ＜1 | ＜1 | ＜1 | ＜1 | ＜1 | ＜1 | Yang et al., 2022 |
| 101 | 1-Octanol | 16.33 | 111-87-5 | 0.022 | Green, citrus, fatty, coconut-like | C8H18O | 1059 | 1070-S | 2.57±0.27 | 7.1±0.92 | 4.66±0.38 | 5.6±1.16 | 3.53±0.4 | 8.74±0.23 | 6.42±0.39 | 11±0.46 | 9.42±0.33 | 116.74 | 322.59 | 212.03 | 254.5 | 160.67 | 397.46 | 291.78 | 499.9 | 428.02 | Guo, Ho, Schwab, & Wan, 2021a |
| 102 | Linalool oxide I | 16.46 | 5989-33-3 | 0.5 | Sweet, floral, creamy | C10H18O2 | 1063 | 1074-S | 55.73±29.06 | #N/A | 106.38±4.33 | 223.06±119.74 | 58.73±2.61 | #N/A | #N/A | 202.26±2.73 | 188.43±13.48 | 111.46 | ＜1 | 212.75 | 446.13 | 117.45 | ＜1 | ＜1 | 404.52 | 376.86 | Xiao et al., 2018 |
| 103 | Linalool oxide II | 17.06 | 34995-77-2 | 0.19 | Sweet, floral, creamy | C10H18O2 | 1079 | 1086-S | 59.33±5.84 | 310.23±82.18 | 149.53±5.75 | 177.38±32.32 | 87.53±6.11 | 381.76±111.99 | 232.84±71.52 | 368.08±147.53 | 322.05±100.39 | 312.27 | 1632.76 | 786.99 | 933.57 | 460.66 | 2009.24 | 1225.45 | 1937.29 | 1695.02 | Xiao et al., 2018 |
| 104 | linalool | 17.46 | 78-70-6 | 0.22 | Floral, sweet, grape-like, woody | C10H18O | 1090 | 1099-S | 99.1±10.32 | 153.42±23.9 | 202.33±10.13 | 144.03±32.54 | 80.77±7.87 | 273.83±8.05 | 158.06±5.97 | 190.66±6.62 | 239.28±4.54 | 450.46 | 697.36 | 919.68 | 654.68 | 367.14 | 1244.69 | 718.44 | 866.63 | 1087.62 | Guo, Schwab, Ho, Song, & Wan, 2022 |
| 105 | Benzeneethanol | 17.93 | 1960/12/8 | 390 | Honey, spice, rose, lilac | C8H10O | 1103 | 1116-S | 12.04±1 | 16.91±0.44 | 14.21±0.43 | 13.29±2.36 | 12.32±1.65 | 21.43±1.69 | 11.45±0.46 | 20.2±2.47 | 20.86±1.39 | ＜1 | ＜1 | ＜1 | ＜1 | ＜1 | ＜1 | ＜1 | ＜1 | ＜1 | Guo, Ho, Schwab, & Wan, 2021a |
| 106 | Linalool oxide III | 19.99 | 39028-58-5 | 3000 | Floral, honey-like | C10H18O2 | 1160 | 1173-S | 94.56±5.63 | 166.6±12.67 | 126.26±1.37 | 126.49±32.01 | 98.38±6.71 | 201.15±10.83 | 89.19±50.99 | 204.83±18.56 | 208.29±2.12 | ＜1 | ＜1 | ＜1 | ＜1 | ＜1 | ＜1 | ＜1 | ＜1 | ＜1 | Guo, Ho, Schwab, & Wan, 2021a |
| 107 | 4-Terpineol | 20.33 | 562-74-3 | 4370 | Spicy, woody, earthy, citrus | C10H18O | 1169 | 1177-S | 3.95±0.09 | 14.21±0.2 | 8.5±0.39 | 9.82±3.68 | 5.66±0.42 | 15.07±0.64 | 11.09±0.92 | #N/A | 13.25±0.5 | ＜1 | ＜1 | ＜1 | ＜1 | ＜1 | ＜1 | ＜1 | ＜1 | ＜1 | Guo, Schwab, Ho, Song, & Wan, 2022 |
| 108 | α-Terpineol | 20.79 | 98-55-5 | 330 | Pleasant, floral | C10H18O | 1182 | 1189-S | 62.89±5.24 | 140.15±11.09 | 123.31±2.84 | 114.17±22.67 | 88.43±5.93 | 177.57±14.69 | 127.45±3.5 | 179.15±8.11 | 180.38±6.21 | ＜1 | ＜1 | ＜1 | ＜1 | ＜1 | ＜1 | ＜1 | ＜1 | ＜1 | Guo, Ho, Schwab, & Wan, 2021a |
| 109 | Nerol | 22.06 | 106-25-2 | 49 | Fresh, citrus, floral, green, sweet, lemon-like | C10H18O | 1218 | 1228-S | 9.86±3.16 | 14.23±0.6 | 13.1±0.39 | 15.71±5.6 | 11.62±3.8 | 14.95±7.95 | 10.49±6.74 | 19±2.45 | 26.59±3.11 | ＜1 | ＜1 | ＜1 | ＜1 | ＜1 | ＜1 | ＜1 | ＜1 | ＜1 | Guo, Ho, Schwab, & Wan, 2021a |
| 110 | 1-Dodecanol | 30.13 | 112-53-8 | 16 | Sweet, fatty | C12H26O | 1461 | 1474-S | 156.29±7.86 | 222.17±27.15 | 356.53±22.74 | 59±19.29 | 421.17±19.45 | 351.7±35.45 | 28.09±5.19 | 244.8±7.64 | 386.06±5.56 | 9.77 | 13.89 | 22.28 | 3.69 | 26.32 | 21.98 | 1.76 | 15.3 | 24.13 | Zhou et al., 2023 |
| 111 | Nerolidol | 32.86 | 142-50-7 | 0.25 | Malic, rose, woody | C15H26O | 1553 | 1544-S | 1.21±0.02 | #N/A | #N/A | #N/A | #N/A | #N/A | #N/A | #N/A | #N/A | 4.85 | ＜1 | ＜1 | ＜1 | ＜1 | ＜1 | ＜1 | ＜1 | ＜1 | Guo, Ho, Schwab, & Wan, 2021a |
| 112 | benzyl alcohol | 14.94 | 100-51-6 | 0.1 | Floral, rose-like, phenolic, balsamic | C7H8O | 1023 | 1036-S | #N/A | 4.66±2.6 | 1.41±0.52 | 2.33±0.16 | 1.92±0.68 | 3.33±0.99 | #N/A | 2.22±0.13 | 3.07±0.44 | ＜1 | 46.58 | 14.07 | 23.32 | 19.2 | 33.32 | ＜1 | 22.23 | 30.7 | Yang et al., 2023 |
| 113 | Levomenthol | 20.14 | 2216-51-5 | 0.2 | Peppermint, cooling, minty | C10H20O | 1164 | 1175-S | #N/A | 2.22±0.18 | 1.75±0.14 | 2.34±0.18 | 1.81±0.94 | 2.25±0.15 | #N/A | 4.07±0.54 | 3.93±0.07 | ＜1 | 11.09 | 8.74 | 11.68 | 9.03 | 11.23 | ＜1 | 20.37 | 19.64 | Guo, Ho, Schwab, & Wan, 2021a |
| 114 | α,α-4-Trimethylbenzenemethanol | 20.61 | 1197-01-9 | 5100 | Citrus, Must | C10H14O | 1177 | 1183-S | #N/A | 2.39±0.19 | 1.61±0.05 | 2.24±1.68 | 1.57±0.34 | 3.02±0.15 | 1.73±0.11 | 2.51±0.12 | 2.97±0.32 | ＜1 | ＜1 | ＜1 | ＜1 | ＜1 | ＜1 | ＜1 | ＜1 | ＜1 | Guo, Schwab, Ho, Song, & Wan, 2022 |
| 115 | (E)-Nerolidol | 32.87 | 40716-66-3 | 250 | Floral, green, citrus, woody, waxy | C15H26O | 1553 | 1564-S | #N/A | 2.23±0.05 | 4.03±0.33 | 1.96±0.64 | 2.28±0.72 | 3.6±0.95 | 2.14±0.28 | #N/A | 5.01±0.23 | ＜1 | ＜1 | ＜1 | ＜1 | ＜1 | ＜1 | ＜1 | ＜1 | ＜1 | Guo, Schwab, Ho, Song, & Wan, 2022 |
| 116 | 1-Heptanol | 12.59 | 111-70-6 | 425 | Leafy green, with vegetative and fruity odor | C7H16O | 959 | 970-S | #N/A | #N/A | #N/A | #N/A | #N/A | #N/A | 0.97±0.02 | 1.5±0.05 | #N/A | ＜1 | ＜1 | ＜1 | ＜1 | ＜1 | ＜1 | ＜1 | ＜1 | ＜1 | Guo, Ho, Schwab, & Wan, 2021a |
| 117 | Eucalyptol | 14.93 | 470-82-6 | 0.069 | camphor scent, refreshing herbal taste | C10H18O | 1022 | 1032-S | #N/A | #N/A | #N/A | #N/A | #N/A | #N/A | 1.64±0.51 | 1.84±0.18 | #N/A | ＜1 | ＜1 | ＜1 | ＜1 | ＜1 | ＜1 | 23.71 | 26.63 | ＜1 | Wang et al., 2022b |
| 118 | 1-Ethyl-1H-pyrrole | 7.33 | 617-92-5 | 10000 | Burnt, roasted | C6H9N | 801 | 821-S | 2.29±0.27 | 1.89±0.18 | 4.82±0.7 | 1.46±0.2 | 2.04±0.48 | 4.12±0.8 | 2.51±0.76 | 2.72±0.99 | 3.15±0.91 | ＜1 | ＜1 | ＜1 | ＜1 | ＜1 | ＜1 | ＜1 | ＜1 | ＜1 | Guo, Ho, Schwab, & Wan, 2021a |
| 119 | 2-Amylfuran | 13.39 | 3777-69-3 | 5.8 | Fruity, green, earthy beany with vegetable like | C9H14O | 980 | 993-S | 28.21±2.63 | 27.46±12.46 | 31.82±1.7 | 24.09±8.16 | 26.58±10 | 33.49±2.52 | 29.29±1.97 | 37.65±1.32 | 43.14±1.65 | 4.86 | 4.74 | 5.49 | 4.15 | 4.58 | 5.77 | 5.05 | 6.49 | 7.44 | Guo, Schwab, Ho, Song, & Wan, 2022 |
| 120 | tea pyrrole | 15.59 | 2167-14-8 | 65000 | Burnt, roasted, smoky | C7H9NO | 1040 | 1046-S | 23.75±0.55 | #N/A | #N/A | 51.74±13.08 | 34.33±4.53 | #N/A | 50.05±1.08 | 97.04±0.68 | 99.3±0.82 | ＜1 | ＜1 | ＜1 | ＜1 | ＜1 | ＜1 | ＜1 | ＜1 | ＜1 | Guo, Ho, Schwab, & Wan, 2021a |
| 121 | Dibenzofuran | 31.59 | 132-64-9 | 3.3 | woody | C12H8O | 1509 | 1515-S | 5.68±0.54 | 7.96±0.61 | 8.09±0.5 | 5.5±0.88 | 7.49±2.03 | 9.24±1.33 | 6.46±0.3 | 8.42±0.37 | 9.65±0.45 | 1.72 | 2.41 | 2.45 | 1.67 | 2.27 | 2.8 | 1.96 | 2.55 | 2.92 | Wen et al., 2023 |
| 122 | 2-Ethylpyridine | 10.34 | 100-71-0 | 13.01 | Sweet, cucumber | C7H9N | 897 | 906-S | #N/A | 2.65±0.34 | 2.64±0.42 | 1.56±0.37 | 1.49±0.08 | 5.86±0.62 | 1.29±0.22 | 3.9±0.59 | 5.9±0.23 | ＜1 | ＜1 | ＜1 | ＜1 | ＜1 | ＜1 | ＜1 | ＜1 | ＜1 | Wen et al., 2023 |
| 123 | 2,3-Dihydrobenzofuran | 21.68 | 496-16-2 | 2 | Chemical tar-like, phenolic, smoky, stryrene-like | C8H8O | 1207 | 1224-S | #N/A | #N/A | 4.69±1.38 | #N/A | 2.51±0.77 | 5.24±1.3 | #N/A | 3.39±0.04 | 4.7±0.08 | ＜1 | ＜1 | 2.35 | ＜1 | 1.25 | 2.62 | ＜1 | 1.7 | 2.35 | Guo, Schwab, Ho, Song, & Wan, 2022 |
| 124 | 2,3,5,6-tetramethylpyrazine | 16.93 | 1124-11-4 | 8.6 | Cocoa, Coffee, Green, Mocha, Roast | C8H12N2 | 1076 | 1088-S | #N/A | #N/A | #N/A | #N/A | #N/A | #N/A | #N/A | 4.5±0.44 | 3.39±1.6 | ＜1 | ＜1 | ＜1 | ＜1 | ＜1 | ＜1 | ＜1 | ＜1 | ＜1 | Jiang et al., 2023 |

a CAS: the published chemical abstracts service (CAS) of compounds in NIST 17 library. b: The data of concentrations is “mean ± standard” deviation of the volatile compound content; “#N/A” means the missing concentration and ROAV. c: reference are as follows.

**References**

Deng, X., Huang, G., Tu, Q., Zhou, H., Li, Y., Shi, H., et al. (2021). Evolution analysis of flavor-active compounds during artificial fermentation of Pu-erh tea. *Food Chemistry*, 357: 129783. http://doi.org/ 10.1016/j.foodchem.2021.129783.

Fang, X., Liu, Y., Xiao, J., Ma, C., & Huang, Y. (2023). GC–MS and LC-MS/MS metabolomics revealed dynamic changes of volatile and non-volatile compounds during withering process of black tea. *Food Chemistry*, 410: 135396. http://doi.org/ 10.1016/j.foodchem.2023.135396.

Flaig, M., Qi, S. C., Wei, G., Yang, X., & Schieberle, P. (2020). Characterisation of the key aroma compounds in a Longjing green tea infusion (Camellia sinensis) by the sensomics approach and their quantitative changes during processing of the tea leaves. *European Food Research and Technology*, 246(12): 2411-2425. http://doi.org/10.1007/s00217-020-03584-y.

Gong, X., Han, Y., Zhu, J., Hong, L., Zhu, D., Liu, J., et al. (2017). Identification of the aroma-active compounds in Longjing tea characterized by odor activity value, gas chromatography- olfactometry, and aroma recombination. *International journal of food properties*, 20: S1107-S1121. http://doi.org/10.1080/10942912.2017.1336719.

Guo, X., Ho, C.-T., Schwab, W., & Wan, X. (2021a). Aroma profiles of green tea made with fresh tea leaves plucked in summer. *Food Chemistry*, 363: 130328. http://doi.org/ 10.1016/j.foodchem.2021.130328.

Guo, X., Ho, C. T., Schwab, W., & Wan, X. (2021b). Effect of the roasting degree on flavor quality of large-leaf yellow tea. *Food Chemistry*, 347: 129016. http://doi.org/10.1016/j.foodchem.2021.129016.

Guo, X., Ho, C.-T., Wan, X., Zhu, H., Liu, Q., & Wen, Z. (2021c). Changes of volatile compounds and odor profiles in Wuyi rock tea during processing. *Food Chemistry*, 341: 128230. http://doi.org/10.1016/j.foodchem.2020.128230.

Guo, X., Schwab, W., Ho, C. T., Song, C., & Wan, X. (2022). Characterization of the aroma profiles of oolong tea made from three tea cultivars by both GC–MS and GC-IMS. *Food Chemistry*, 376: 131933.http://doi.org/10.1016/j.foodchem.2021.131933.

Huang, W., Zhang, C., Gu, Z., Li, C., Fang, Z., Zeng, Z., et al. (2022). Effect of microbial fermentation on the sensory characteristics and chemical compositions of Chinese sweet tea (Lithocarpus litseifolius (Hance) Chun). *Food Bioscience*, 46: 101567. http://doi.org/ 10.1016/j.fbio.2022.101567.

Jiang, B., Yang, L., Luo, X., Huang, R., Jiao, W., Zhong, X., et al. (2023). Aroma Formation and Dynamic Changes during Sichuan Black Tea Processing by GC–MS-Based Metabolomics. *Fermentation*, 9: 686. <http://doi.org/10.3390/fermentation9070686>.

Jiang, R. G., Huang, Y., Jin, Y. L., Huang, F. F., Liu, Z. H., Huang, J. A., et al. (2021a). Analysis of Characteristic Aroma Components of Different Grades of Yellow Tea. *The Food Science*, 42(16):10, (in Chinese with English abstract). http://doi.org/10.7506/spkx1002-6630-20200709-137.

Jiang, R. G., Huang, Y., Jin, Y. L., Li, Y. D., Huang, J. A., & Li, Q. (2021b). Study of Aroma Compounds and Their Source in Fu Brick Tea. *Modern Food Science and Technology*, 40(09):101-111. (in Chinese with English abstract). https://doi.org/ 10.3969/j.issn.1673-1689.2021.09.013.

Jin, Y. L., Huang, T., Jiang, R. G., Huang, F. F., Liu, Z. H., Huang, J. A., et al. (2021). Characteristic volatile components of different types of fermented brick tea. *Food and Fermentation Industries*， 47(3): 188-196, (in Chinese with English abstract). http://doi.org/10.13995/j.cnki.11-1802/ts.025075.

Li, Q., Li, B., Zhang, C., Zhou, X., Liu, W., Mi, Y., et al. (2024). Insights into key aroma of vine tea (Ampelopsis grossedentata) for grade evaluation integrating relative odor activity value, gas chromatography-olfactometry and chemometrics approaches. *Food Control*, 155: 110048. http://doi.org/ 10.1016/j.foodcont.2023.110048.

Liu, C., Wang, C., Zheng, T., Zhao, M., Gong, W., Wang, Q., et al. (2022). Characterization of Key Odor-Active Compounds in Sun-Dried Black Tea by Sensory and Instrumental-Directed Flavor Analysis. *Foods*, 11(12). http://doi.org/10.3390/foods11121740.

Liu, H., Xu, Y., Wen, J., An, K., wu, J., Yu, Y., et al. (2021a). A comparative study of aromatic characterization of Yingde Black Tea infusions in different steeping temperatures. *LWT - Food Science and Technology*, 143: 110860.http://doi.org/ 10.1016/j.lwt.2021.110860.

Liu, H., Xu, Y., Wu, J., Wen, J., Yu, Y., An, K., et al. (2021b). GC-IMS and olfactometry analysis on the tea aroma of Yingde black teas harvested in different seasons. *Food Research International*, 150: 110784. http://doi.org/10.1016/j.foodres.2021.110784.

Ma, L., Gao, M., Hu, J., Tong, W., Du, L., Yu, A., et al. (2022). Characterization of the key active aroma compounds in Pu-erh tea using gas chromatography–time of flight/mass spectrometry–olfactometry combined with five different evaluation methods. *European Food Research and Technology*, 248(1): 45-56. http://doi.org/10.1007/s00217-021-03847-2.

Ma, L., Sun, Y., Wang, X., Zhang, H., Zhang, L., Yin, Y., et al. (2023). The characteristic of the key aroma‐active components in white tea using GC‐TOF‐MS and GC‐olfactometry combined with sensory‐directed flavor analysis. *Journal of the Science of Food and Agriculture*, 103. http://doi.org/10.1002/jsfa.12798.

Ni, H., Jiang, Q., Lin, Q., Ma, Q., Wang, L., Weng, S., et al. (2021). Enzymatic hydrolysis and auto-isomerization during β-glucosidase treatment improve the aroma of instant white tea infusion. *Food Chemistry*, 342: 128565. http://doi.org/10.1016/j.foodchem.2020.128565.

Pang, X., Yu, W., Cao, C., Yuan, X., Qiu, J., Kong, F., et al. (2019). Comparison of Potent Odorants in Raw and Ripened Pu-Erh Tea Infusions Based on Odor Activity Value Calculation and Multivariate Analysis: Understanding the Role of Pile Fermentation. *Journal of Agricultural and Food Chemistry*, 67(47): 13139-13149. http://doi.org/10.1021/acs.jafc.9b05321.

Qi, H., Ding, S., Pan, Z., Li, X., & Fu, F. (2020). Characteristic Volatile Fingerprints and Odor Activity Values in Different Citrus-Tea by HS-GC-IMS and HS-SPME-GC-MS. *Molecules*, 25(24). http://doi.org/10.3390/molecules25246027.

Rigling, M., Liu, Z., Hofele, M., Prozmann, J., Zhang, C., Ni, L., et al. (2021). Aroma and catechin profile and in vitro antioxidant activity of green tea infusion as affected by submerged fermentation with Wolfiporia cocos (Fu Ling). *Food Chemistry*, 361: 130065. http://10.1016/j.foodchem.2021.130065.

Sun, Z., Lin, Y., Yang, H., Zhao, R., Zhu, J., & Wang, F. (2024). Characterization of honey-like characteristic aroma compounds in Zunyi black tea and their molecular mechanisms of interaction with olfactory receptors using molecular docking. *LWT-Food Science and Technology*, 191: 115640.http://doi.org/10.1016/j.lwt.2023.115640.

Wang, C., Li, J., Zhang, Y., He, Z., Zhang, Y., Zhang, X., et al. (2022a). Effects of electrostatic spray drying on the sensory qualities, aroma profile and microstructural features of instant Pu-erh tea. *Food Chemistry*, 373: 131546.http://doi.org/10.1016/j.foodchem.2021.131546.

Wang, Z., Su, D., Ren, H., Lv, Q., Ren, L., Li, Y., et al. (2022c). Effect of different drying methods after fermentation on the aroma of Pu-erh tea (ripe tea). *LWT-Food Science and Technology*, 171: 114129.http:/10.1016/j.lwt.2022.114129.

Wen, S., Jiang, R., An, R., Ouyang, J., Liu, C., Wang, Z., et al. (2023). Effects of pile-fermentation on the aroma quality of dark tea from a single large-leaf tea variety by GC × GC-QTOFMS and electronic nose. *Food Research International*, 174: 113643. http://doi.org/10.1016/j.foodres.2023.113643.

Xiao, Z., Cao, X., Zhu, J., Chen, F., & Niu, Y. (2022). Characterization of the key aroma compounds in three world-famous black teas. *European Food Research and Technology*, 248(9): 2237-2252. http://doi.org/10.1007/s00217-022-04039-2.

Xiao, Z. B., Wang, H. L., Niu, Y. W., Zhu, J. C., & Ma, L. (2018) Analysis of aroma components in four Chinese congou black teas by odor active values and aroma extract dilution analysis coupled with partial least squares regression. *Food Science*, 39(10): 242-249. (in Chinese with English abstract). http://doi.org/10.7506/spkx1002-6630-201810037.

Xu, S., Zeng, X., Wu, H., Shen, S., Yang, X., Deng, W.-W., et al. (2021). Characterizing volatile metabolites in raw Pu’er tea stored in wet-hot or dry-cold environments by performing metabolomic analysis and using the molecular sensory science approach. *Food Chemistry*, 350: 129186. http://doi.org/10.1016/j.foodchem.2021.129186.

Yang, P., Song, H., Lin, Y., Guo, T., Wang, L., Granvogl, M., et al. (2021). Differences of characteristic aroma compounds in Rougui tea leaves with different roasting temperatures analyzed by switchable GC-O-MS and GC × GC-O-MS and sensory evaluation. *Food Function*, 12(11): 4797-4807. http://doi.org/10.1039/d1fo00165e.

Yang, P., Wang, H., Cao, Q., Song, H., Xu, Y., & Lin, Y. (2023). Aroma-active compounds related to Maillard reaction during roasting in Wuyi Rock tea. *Journal of Food Composition and Analysis*, 115: 104954. http://doi.org/10.1016/j.jfca.2022.104954.

Yang, Y., Zhu, H., Chen, J., Xie, J., Shen, S., Deng, Y., et al. (2022). Characterization of the key aroma compounds in black teas with different aroma types by using gas chromatography electronic nose, gas chromatography-ion mobility spectrometry, and odor activity value analysis. *LWT-Food Science and Technology*, 163: 113492. http://doi.org/10.1016/j.lwt.2022.113492.

Yin, X., Xiao, Y., Wang, K., Wu, W., Huang, J., Liu, S., et al. (2023). Effect of shaking manners on floral aroma quality and identification of key floral-aroma-active compounds in Hunan black tea. *Food Research International*, 174: 113515. http://doi.org/10.1016/j.foodres.2023.113515.

Zhou, J., He, C., Qin, M., Luo, Q., Jiang, X., Zhu, J., et al. (2023). Characterizing and Decoding the Effects of Different Fermentation Levels on Key Aroma Substances of Congou Black Tea by Sensomics. *Journal of Agricultural and Food Chemistry*, 71(40): 14706-14719. http://doi.org/10.1021/acs.jafc.3c02813.

Zhu, J., Niu, Y., & Xiao, Z. (2021). Characterization of the key aroma compounds in Laoshan green teas by application of odour activity value (OAV), gas chromatography-mass spectrometry-olfactometry (GC-MS-O) and comprehensive two-dimensional gas chromatography mass spectrometry (GC×GC-qMS). *Food Chemistry*, 339: 128136. http:// 10.1016/j.foodchem.2020.128136.

Zhu, Y., Lv, H.-P., Shao, C.-Y., Kang, S., Zhang, Y., Guo, L., et al. (2018). Identification of key odorants responsible for chestnut-like aroma quality of green teas. *Food Research International*, 108: 74-82. http://doi.org/ 10.1016/j.foodres.2018.03.026.

Zhu, Y., Yang, T., Shi, J., Yu, F. L., Dai, W. D., Tang, J. F., et al. (2015). Analysis of Aroma Components in Xihu Longjing Tea by Comprehensive Two-Dimensional Gas Chromatography Time-of-Flight Mass Spectrometry. *Scientia Agricultura Sinica*, 48:4120-4146. http://doi.org/10.3864/j.issn.0578-1752.2015.20.013.
